# Analysis of Differentially Expressed Genes in the Dentate Gyrus and Anterior Cingulate Cortex in a Mouse Model of Depression

**DOI:** 10.1155/2021/5013565

**Published:** 2021-02-11

**Authors:** Yicong Wei, Keming Qi, Yi Yu, Wei Lu, Wei Xu, Chengzi Yang, Yu Lin

**Affiliations:** College of Pharmacy, Fujian University of Traditional Chinese Medicine, Fuzhou 350122, China

## Abstract

Major depressive disorder (MDD) is a prevalent, chronic, and relapse-prone psychiatric disease. However, the intermediate molecules resulting from stress and neurological impairment in different brain regions are still unclear. To clarify the pathological changes in the dentate gyrus (DG) and anterior cingulate cortex (ACC) regions of the MDD brain, which are the most closely related to the disease, we investigated the published microarray profile dataset GSE84183 to identify unpredictable chronic mild stress- (UCMS-) induced differentially expressed genes (DEGs) in the DG and ACC regions. Based on the DEG data, functional annotation, protein-protein interaction, and transcription factor (TF) analyses were performed. In this study, 1071 DEGs (679 upregulated and 392 downregulated) and 410 DEGs (222 upregulated and 188 downregulated) were identified in DG and ACC, respectively. The pathways and GO terms enriched by the DEGs in the DG, such as cell adhesion, proteolysis, ion transport, transmembrane transport, chemical synaptic transmission, immune system processes, response to lipopolysaccharide, and nervous system development, may reveal the molecular mechanism of MDD. However, the DEGs in the ACC involved metabolic processes, proteolysis, visual learning, DNA methylation, innate immune responses, cell migration, and circadian rhythm. Sixteen hub genes in the DG (Fn1, Col1a1, Anxa1, Penk, Ptgs2, Cdh1, Timp1, Vim, Rpl30, Rps21, Dntt, Ptk2b, Jun, Avp, Slit1, and Sema5a) were identified. Eight hub genes in the ACC (Prkcg, Grin1, Syngap1, Rrp9, Grwd1, Pik3r1, Hnrnpc, and Prpf40a) were identified. In addition, eleven TFs (Chd2, Zmiz1, Myb, Etv4, Rela, Tcf4, Tcf12, Chd1, Mef2a, Ubtf, and Mxi1) were predicted to regulate more than two of these hub genes. The expression levels of ten randomly selected hub genes that were specifically differentially expressed in the MDD-like animal model were verified in the corresponding regions in the human brain. These hub genes and TFs may be regarded as potential targets for future MDD treatment strategies, thus aiding in the development of new therapeutic approaches to MDD.

## 1. Introduction

Major depressive disorder (MDD) is a chronic mental illness affecting individuals worldwide with a high morbidity rate. It often leads to a reduction in the patient's quality of life or even death. Humans may suffer from depression at any stage, from childhood to old age [1]. The World Health Organization predicts that depression will become the main cause of human disability and one of the main disease burdens. At present, although some antidepressant drugs have been successfully used in the clinic, they are effective in only 30%-40% of patients with depression and often have various side effects [2]. These antidepressants are based on different hypotheses regarding the etiology of depression, such as monoamine dysfunction, neurogenesis, the corticotrophin-releasing factor (CRF) receptor, ketamine, and the N-methyl-D-aspartate (NMDA) receptor. These hypotheses explain the causes of depression to different degrees and provide different targets for the development of new antidepressant drugs. The drugs developed based on these hypotheses have different targets and modes of action but are still not fully effective in treating depression [1–3]. Due to the complexity of the disease, new antidepressant drug development has had a high failure rate in recent decades. Therefore, it is necessary to conduct an in-depth study on the pathogenesis of the disease and find new therapeutic targets. However, the intermediate molecules resulting from stress and neurological impairment in different brain regions are still unclear.

MDD is associated with dysfunction in multiple brain regions, including the cortex, midline, amygdala, nucleus accumbens, and hippocampus [4]. Early research emphasized that MDD is closely related to lesions in the hippocampal CA3 area [5]. However, the dentate gyrus (DG) in the hippocampus reportedly plays a major role in the antidepressant effect of the selective serotonin reuptake inhibitor (SSRI) class [6]. The DG has a fundamental role in emotional regulation and behavior [7, 8]. Recent studies have indicated that long-term depression can lead to reduced DG volumes in animals and clinical subjects [9, 10]. The abovementioned studies have demonstrated that the DG plays a key role in MDD. In addition, the anterior cingulate cortex (ACC) is considered to be an information processing center for emotion, social interaction, and cognition [11–13]. Studies have shown that the ACC is closely related to depression in adolescents. ACC functional changes may be due to genetic changes [14], environmental poverty [15], or exposure to stress [16] or abuse [17]. Although the DG and ACC are considered two key areas of the brain involved in MDD, the molecular mechanisms and key genes underlying their pathogenicity are still unclear and need to be further studied [18, 19].

Genetic analyses of dermal cells and blood have led to the identification of numerous genes related to MDD genetic susceptibility [20–23]. However, these data may not reflect whether the stress environment affects the expression of these molecules in the brain or their role in the pathological changes in different brain regions in MDD. Therefore, it is necessary to analyze the gene expression in brain regions related to depression, especially the DG and ACC regions [24–26]. High-throughput sequencing technology has become an effective method to explore pathogenesis and identify the key genes underlying the pathogenicity of various diseases [27–29]. In this study, we downloaded the microarray profile dataset GSE84183 from the Gene Expression Omnibus (GEO) database and identified unpredictable chronic mild stress- (UCMS-) induced differentially expressed genes (DEGs) in the DG and ACC regions. Gene Ontology (GO) and pathway enrichment analyses of the DEGs were conducted, and protein-protein interaction networks were then constructed and analyzed. The hub genes were identified, and the functions of the modules with the hub genes were analyzed. Then, the transcription factors potentially regulating these hub genes were screened. Finally, the published Human Protein Atlas (HPA) database was used to verify the expression of hub genes in the corresponding regions in the human brain. Receiver operating characteristic (ROC) curve analysis was performed to analyze the specificity of the differential expression of hub genes in mice with UCMS-induced depression. Our research may provide new clues for exploring the pathological mechanism of MDD and finding drug targets for MDD treatment.

## 2. Materials and Methods

### 2.1. Gene Expression Profiles

The gene expression profile dataset GSE84183 used in this study was downloaded from the GEO database (http://www.ncbi.nlm.nih.gov/geo/), and all data were all based on the GPL13912 (Agilent-028005 SurePrint G3 Mouse GE 8x60K Microarray) platform. This platform provided gene expression profile data of 16 DG and 16 ACC tissue samples (8 UCMS-exposed + 8 controls) and UCMS-exposed mice subjected to unpredictable chronic mild stress procedures for nine weeks [30]. All data were normalized by quantile normalization. The ggplot2 R package was used to display the background correction and normalization of the data [31].

### 2.2. Identification of UCMS-Induced DEGs in the DG and ACC

In this study, we used the empirical Bayes *t*-test (eBayes) to identify UCMS-induced DEGs in the DG and ACC [32]. Among them, DEGs with a fold change > 1.5 (∣log_2_ (fold change) | >0.585) and fold change > 1.2 (∣log_2_ (fold change) | >0.263) in the DG and ACC were used for comparative studies, which were performed using the limma R package for data processing and analysis [32]. A heat map of common DEGs in both the DG and ACC was produced using the “pheatmap” package (version 1.0.10) of R [33]. PCA of UCMS-induced DEGs in the DG and ACC was conducted, and the results showed that the distribution between the control group (8 samples) and the UCMS group (8 samples) was significantly different (Figure S2). In addition, PCA of DEGs in the DG and ACC showed that the overall distribution of samples was significantly different between the groups (Figure S3). According to the comparative analysis results of DEGs in the DG and ACC, those with a fold change > 1.5 in the DG and a fold change > 1.2 in the ACC were used for subsequent series analysis.

### 2.3. Functional and Pathway Enrichment Analyses of DEGs in the DG and ACC

To investigate the biological functions of DEGs in the DG and ACC, the online software DAVID 6.8 (https://david.ncifcrf.gov/summary.jsp) was used for Gene Ontology (GO) analysis. DEGs were subjected to functional enrichment analysis of biological processes (BPs), molecular functions (MFs), and cellular components (CCs). The online database GenomeNet Database Resources (https://www.genome.jp) was applied for the Kyoto Encyclopedia of Genes and Genomes (KEGG) pathway analysis. *P* values < 0.05 were considered statistically significant. GO and KEGG analyses were used to perform a comprehensive functional analysis of DEGs in the DG and ACC [34].

### 2.4. Comprehensive Analysis of PPI Networks and Modules

To analyze the interactions between the proteins encoded by DEGs in the DG and ACC, the STRING (https://string-db.org/) database was used to predict the interactions between the proteins encoded by DEGs [35]. The active interaction sources included Textmining, Experiments, Databases, Coexpression, Neighborhood, Gene Fusion, and Cooccurrence, with a confidence score > 0.4 being defined as significant. Then, the PPI network was constructed by Cytoscape software (version 3.5.1) [36]. The topological properties of the PPI network, including the node degree and betweenness centrality, were determined. The significance of a gene in the network was evaluated by measuring its “betweenness centrality” and “degree,” and the gene nodes with the top scores for both betweenness centrality and degree were identified as hub genes. Furthermore, module analysis was performed by using the PEWCC plugin (version 1.0) to explore the clustering modules, which included the hub genes in the vast PPI network (join parameter > 0.5, overlap threshold > 0.8) [37]. Finally, the ClueGO plugin (version 2.5.6) was used to analyze the biological functions of the clustering modules, and *P* < 0.05 was considered statistically significant [32, 38].

### 2.5. Construction of the Hub Gene-TF Regulatory Network

To further study the transcriptional regulation mechanism of the hub genes, the online software programs NetworkAnalyst (https://www.networkanalyst.ca/) [39] and TRRUST (https://www.grnpedia.org/trrust/) [40] were used to predict transcription factors (TFs) of the hub genes. Among them, NetworkAnalyst predicts the transcription factors of target genes based on ENCODE ChIP-seq data. Only peak intensity signals < 500 and predicted regulatory potential scores < 1 were used (using the BETA Minus algorithm). The TRRUST database was derived from 11,237 PubMed articles. Transcription factors that can simultaneously regulate the transcriptional expression of more than two hub genes were selected for the construction of the hub gene-TF regulatory network, and Cytoscape software (version 3.5.1) was used to construct the network [36].

### 2.6. Validation of Hub Genes

The Human Protein Atlas (HPA) (https://www.proteinatlas.org/) was used to validate the expression of the hub genes in the corresponding regions of the human brain [41]. Receiver operating characteristic (ROC) curve analysis was performed to analyze the specificity of the differential expression of hub genes in UCMS-exposed mice using the R package “pROC.” The area under the curve (AUC) value was used to distinguish UCMS-exposed mice from control mice [42].

## 3. Results

### 3.1. Identification of UCMS-Induced DEGs in the DG and ACC

**Figure 1:**
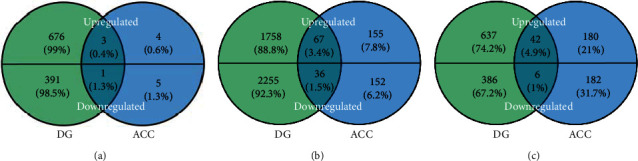
Venn diagram based on the overlapping UCMS-induced DEGs between the DG and ACC. (a) DEGs with a fold change > 1.5 in both the DG and ACC; (b) DEGs with a fold change > 1.2 in both the DG and ACC; (c) DEGs with a fold change > 1.5 in the DG and with a fold change > 1.2 in the ACC.

The boxplot indicated that the GSE84183 data exhibited good normalization (Figure S1). The gene expression profiles of the DG and ACC samples from UCMS-exposed and control mice were used for comparative analysis (Tables S1 and S2). A total of 1071 DEGs (679 upregulated and 392 downregulated) and 13 DEGs (7 upregulated and 6 downregulated) were identified in the DG and ACC, respectively, all having a fold change > 1.5. Only 4 DEGs (3 upregulated and 1 downregulated) were identified in both the DG and ACC. There were 4116 DEGs (1825 upregulated and 2291 downregulated) and 410 DEGs (222 upregulated and 188 downregulated) identified in the DG and ACC, respectively, all with a fold change > 1.2. There were only 103 DEGs in common between the DG and ACC, among which 67 genes were upregulated and 36 genes were downregulated (Figure 1). Hierarchical clustering analysis showed that these 103 DEGs were clustered between the DG and ACC, indicating that these 103 DEGs have different modes of expression between the DG and ACC (Figure S4). To investigate a certain number of DEGs with high fold changes in the DG and ACC, we selected those with a fold change > 1.5 in the DG and those with a fold change > 1.2 in the ACC for subsequent analysis. There were only 48 DEGs in common between the DG and ACC. Among them, 42 genes were upregulated (4.9%) and 6 genes were downregulated (1%).

### 3.2. Functional and Pathway Enrichment Analyses of DEGs in the DG and ACC

Gene Ontology (GO) and pathway enrichment analyses showed that the DEGs that were upregulated in the DG were mainly involved in cell adhesion, proteolysis, ion transport, transmembrane transport, chemical synaptic transmission, immune system processes, and responses to lipopolysaccharide. Their molecular functions included calcium ion binding, calmodulin binding, transporter activity, ion channel activity, protease binding, and heparin binding. The main pathways were mainly associated with neuroactive ligand-receptor interactions, extracellular matrix-receptor interactions, and complement and coagulation cascades. DEGs downregulated in the DG were mainly involved in biological processes such as multicellular organism development, nervous system development, cell differentiation, responses to drugs, and negative regulation of neuronal apoptotic processes. Molecular functions were mainly associated with protein and calcium ion binding. The main pathways involved were the calcium signaling, thyroid hormone synthesis, and hematopoietic cell lineage pathways (Tables S3 and S4).

The upregulated DEGs in the ACC were mainly involved in biological processes such as metabolic processes, proteolysis, visual learning, long-term memory, and DNA methylation. The molecular functions were mainly associated with serine-type peptidase and endopeptidase activity. In addition, toxoplasmosis-related pathways were involved. The downregulated DEGs in the ACC were mainly involved in biological processes such as negative regulation of transcription, positive regulation of gene expression, innate immune responses, cell migration, and circadian rhythm. The molecular functions were mainly associated with protein binding, hydrolase activity, and sequence-specific DNA binding. The main pathways involved were proteoglycans in cancer and signaling pathways regulating the pluripotency of stem cells (Tables S3 and S4).

### 3.3. Comprehensive Analysis of PPI Networks and Modules

**Figure 2:**
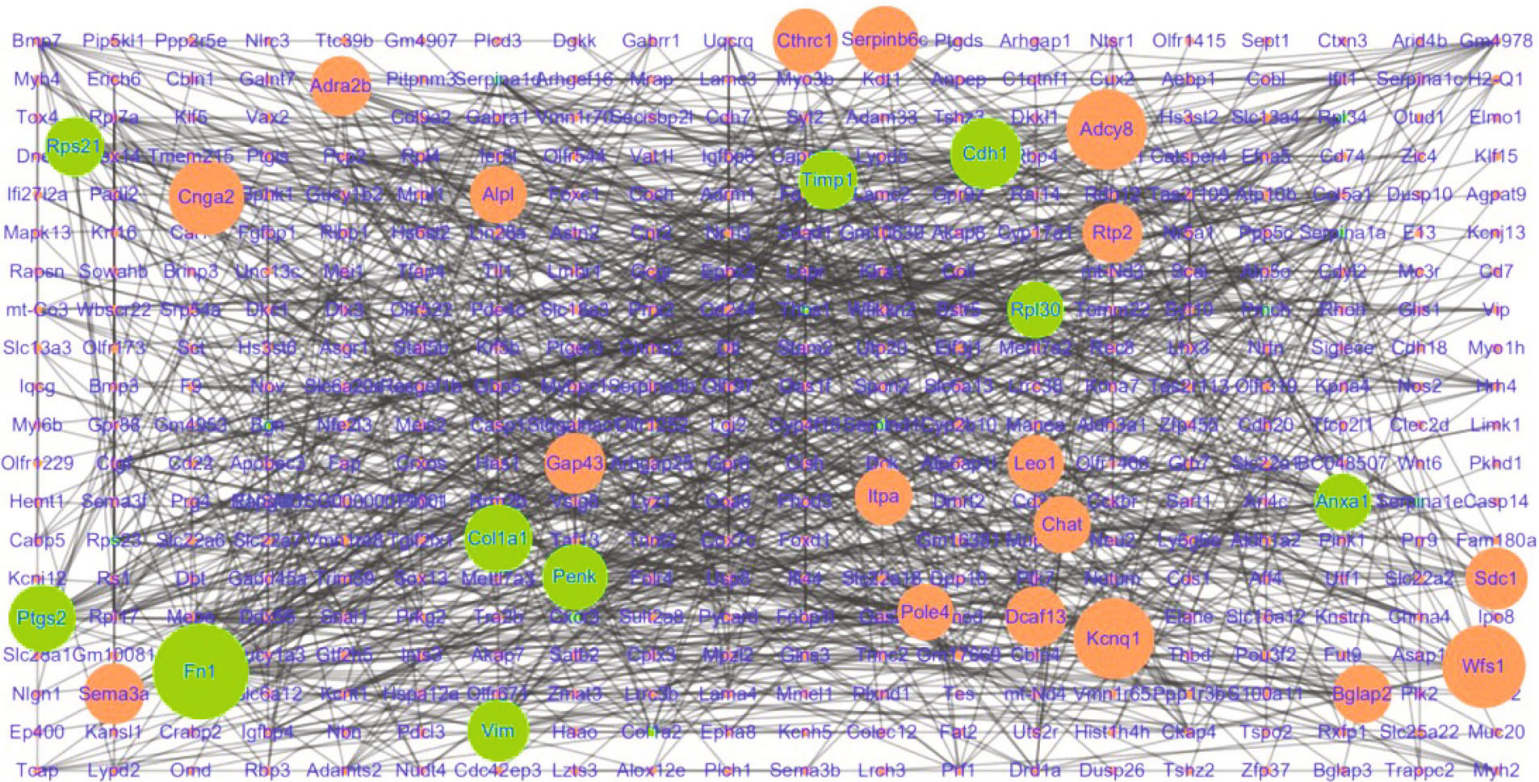
Protein-protein interaction network of upregulated genes in the DG. The green nodes are hub genes (betweenness centrality: 0.0315-0.2065, degree: range 16-44).

**Figure 3:**
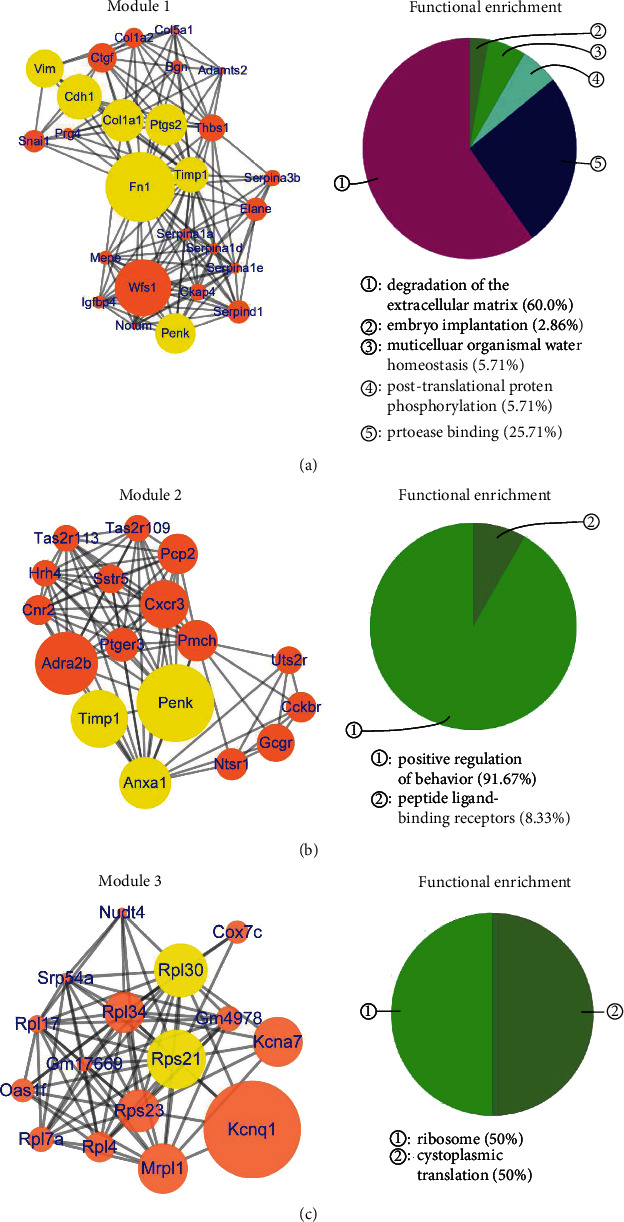
Modules and functional enrichment analysis of the protein-protein interaction (PPI) network of upregulated genes in the DG. (a) Module 1 and functional enrichment analysis of the protein-protein interaction (PPI) network. (Left panel) Module 1 in the protein-protein interaction (PPI) network. The yellow nodes are hub genes. (Right panel) Functional enrichment of module 1. (b) Module 2 and functional enrichment analysis of the protein-protein interaction (PPI) network. (Left panel) Module 2 in the protein-protein interaction (PPI) network. The yellow nodes are hub genes. (Right panel) Functional enrichment of module 2. (c) Module 3 and functional enrichment analysis of the protein-protein interaction (PPI) network. (Left panel) Module 3 in the protein-protein interaction (PPI) network. The yellow nodes are hub genes. (Right panel) Functional enrichment of module 3.

The PPI network of upregulated genes in the DG consisted of 400 nodes and 990 edges (Figure 2) (Table S5), with *R*‐squared = 0.245 and correlation = 0.628 for betweenness centrality and *R*‐squared = 0.888 and correlation = 0.861 for the node degree (Figure S5). Ten hub genes had the top scores for betweenness centrality (range 0.0315 to 0.2065) and the node degree (range 16 to 44), including Fn1, Col1a1, Anxa1, Penk, Ptgs2, Cdh1, Timp1, Vim, Rpl30, and Rps21 (green node in Figure 2). After cluster analysis, the ten hub genes were divided into three modules, with Fn1, Col1a1, Penk, Ptgs2, Cdh1, Timp1, and Vim being clustered in module 1, which mainly involved biological processes such as degradation of the extracellular matrix, protease binding, and posttranslational protein phosphorylation (Figure 3(a)). Anxa1, Timp1, and Penk were clustered in module 2, which mainly involved functions such as positive regulation of behavior and peptide ligand-binding receptors (Figure 3(b)). Rpl30 and Rps21 were clustered in module 3, which mainly involved ribosomal and cytoplasmic translation (Figure 3(c)).

**Figure 4:**
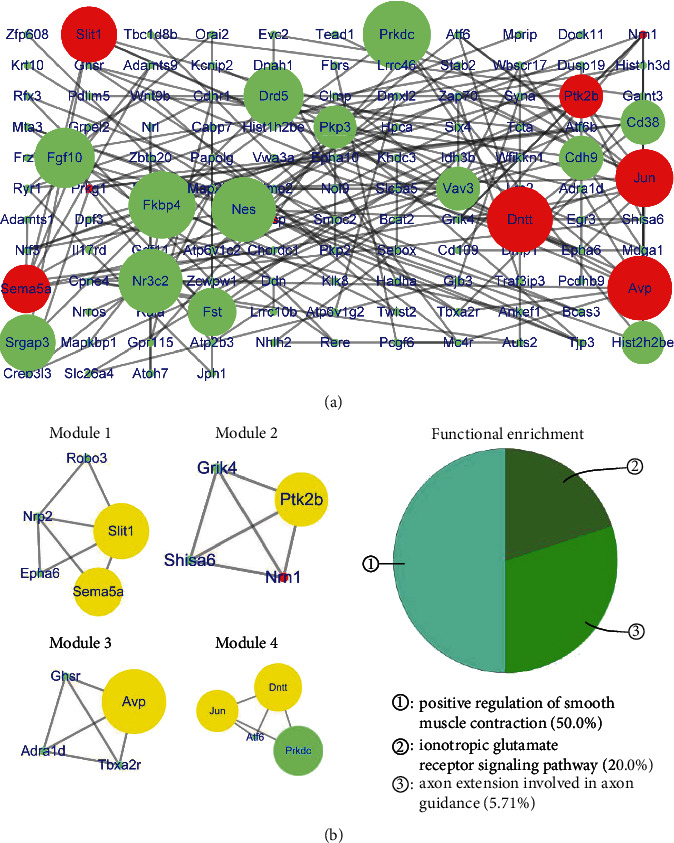
Protein-protein interaction network and modules of downregulated genes in the DG. (a) Protein-protein interaction network of downregulated genes in the DG. The red nodes are hub genes (betweenness centrality: 0.118-0.374, degree: range 5-9). (b) Modules and functional enrichment analysis of the protein-protein interaction (PPI) network. (Left panel) Modules 1-4 in the protein-protein interaction (PPI) network. The yellow nodes are hub genes. (Right panel) Functional enrichment of modules 1-4.

The PPI network of downregulated genes in the DG consisted of 125 nodes and 159 edges (Figure 4(a)) (Table S5), with *R*‐squared = 0.228 and correlation = 0.435 for the betweenness centrality and *R*‐squared = 0.738 and correlation = 0.650 for the node degree (Figure S6). Six hub genes had top scores for betweenness centrality (range 0.118 to 0.374) and the node degree (range 5 to 9), including Dntt, Ptk2b, Jun, Avp, Slit1, and Sema5a (red node in Figure 4(a)). After cluster analysis, the six hub genes were divided into four modules. Slit1 and Sema5a were clustered into module 1. Ptk2b was included in module 2. Avp was included in module 3. Jun and Dntt were clustered into module 4. The functional enrichment analysis of these four modules showed that the functions were associated with positive regulation of smooth muscle contraction, axon extension in axon guidance, and the ionotropic glutamate receptor signaling pathway (Figure 4(b)).

**Figure 5:**
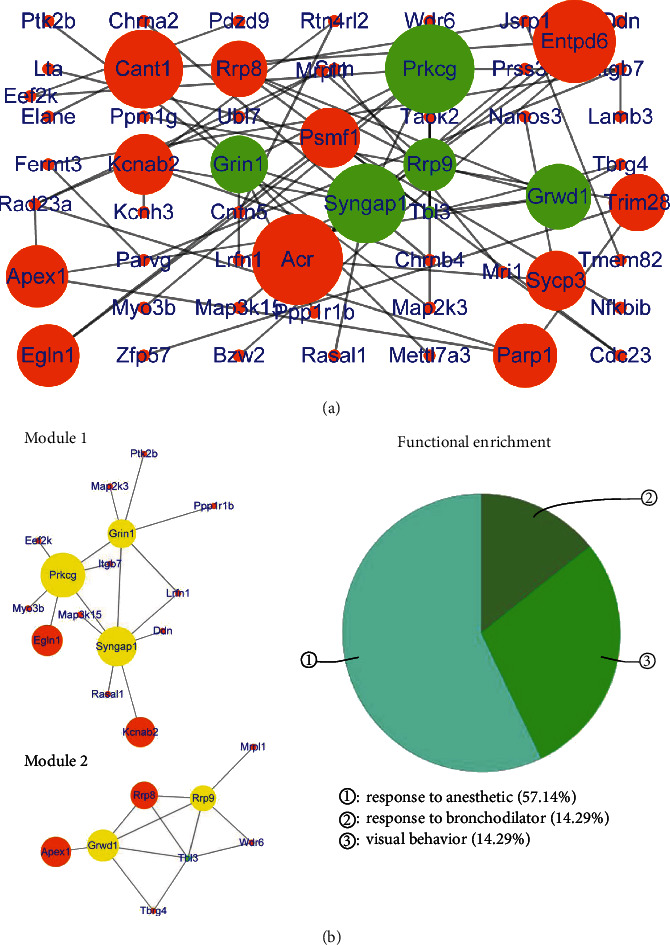
Protein-protein interaction network and modules of upregulated genes in the ACC. (a) Protein-protein interaction network of upregulated genes in the ACC. The green nodes are hub genes (betweenness centrality: 0.2-1.0, degree: range 5-7). (b) Modules and functional enrichment analysis of the protein-protein interaction (PPI) network. (Left panel) Modules 1-2 in the protein-protein interaction (PPI) network. The yellow nodes are hub genes. (Right panel) Functional enrichment of modules 1-2.

The PPI network of upregulated genes in the ACC consisted of 56 nodes and 64 edges (Figure 5(a)) (Table S5), with *R*‐squared = 0.215 and correlation = 0.387 for the betweenness centrality and *R*‐squared = 0.914 and correlation = 0.913 for the node degree (Figure S7). Five hub genes had top scores for betweenness centrality (range 0.2 to 1.0) and the node degree (range 5 to 7), including Prkcg, Grin1, Syngap1, Rrp9, and Grwd1 (green node in Figure 5(a)). After cluster analysis, the five hub genes were divided into two modules, with Prkcg, Grin1, and Syngap1 being clustered in module 1. Rrp9 and Grwd1 were clustered in module 2, and functional enrichment analysis of these two modules showed that the functions were linked to factors such as responses to anesthetics, visual behavior, and responses to bronchodilators (Figure 5(b)).

**Figure 6:**
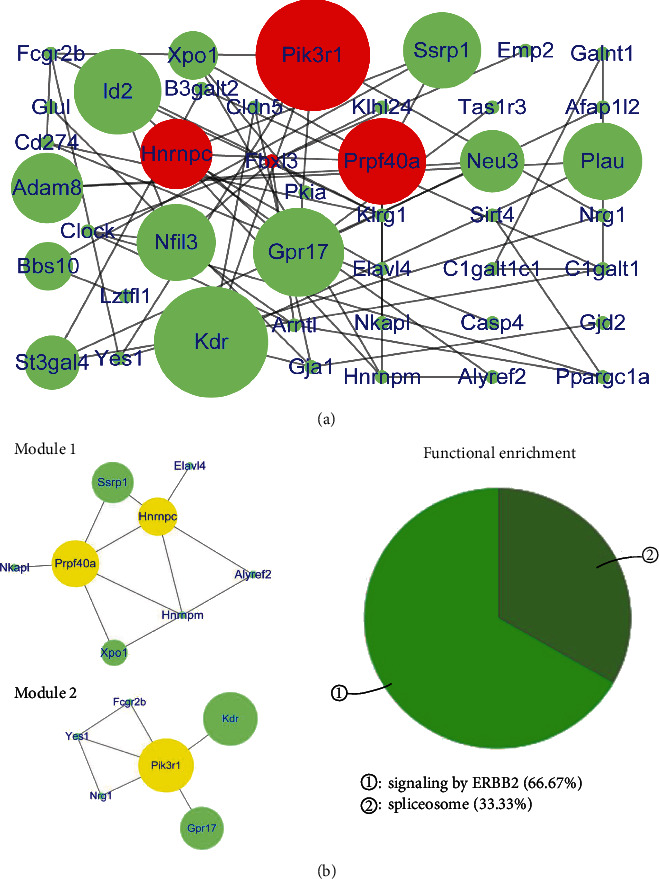
Protein-protein interaction network and modules of downregulated genes in the ACC. (a) Protein-protein interaction network of downregulated genes in the ACC. The red nodes are hub genes (betweenness centrality: 0.2-0.5624, degree = 5). (b) Modules and functional enrichment analysis of the protein-protein interaction (PPI) network. (Left panel) Modules 1-2 in the protein-protein interaction (PPI) network. The yellow nodes are hub genes. (Right panel) Functional enrichment of modules 1-2.

The PPI network of downregulated genes in the ACC consisted of 43 nodes and 56 edges (Figure 6(a)) (Table S5), with *R*‐squared = 0.002 and correlation = 0.095 for the betweenness centrality and *R*‐squared = 0.630 and correlation = 0.834 for the node degree (Figure S8). Three hub genes had top scores for betweenness centrality (range 0.2 to 0.5624) and the node degree (degree = 5), including Hnrnpc, Prpf40a, and Pik3r1 (red node in Figure 6(a)). After cluster analysis, the three hub genes were divided into two modules; Hnrnpc and Prpf40a were clustered in module 1, and Pik3r1 was included in module 2. Functional enrichment analysis of these two modules showed that the functions involved signaling by Erb-b2 receptor tyrosine kinase 2 (ERBB2) and spliceosomes (Figure 6(b)).

### 3.4. Construction of the Hub Gene-TF Regulatory Network

**Figure 7:**
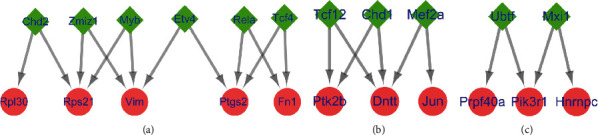
Hub gene-transcription factor (TF) regulatory network. The red nodes denote the hub genes, and the green diamonds denote the transcription factors. (a) Target gene-TF regulatory network of upregulated hub genes in the DG. (b) Target gene-TF regulatory network of downregulated hub genes in the DG. (c) Target gene-TF regulatory network of downregulated hub genes in the ACC.

The transcription factors Chd2, Zmiz1, Myb, Etv4, Rela, and Tcf4 were predicted to regulate more than two of the hub genes upregulated in the DG, including Fn1, Ptgs2, Vim, Rpl30, and Rps21 (Figure 7(a)). The transcription factors Tcf12, Chd1, and Mef2a were predicted to regulate more than two of the hub genes downregulated in the DG, including Dntt, Ptk2b, and Jun (Figure 7(b)); no transcription factor genes were predicted to regulate more than two of the hub genes upregulated in the ACC. The transcription factors Ubtf and Mxi1 were predicted to control more than two of the hub genes downregulated in the ACC, including Hnrnpc, Prpf40a, and Pik3r1 (Figure 7(c)).

### 3.5. Validation of Hub Genes

**Figure 8:**
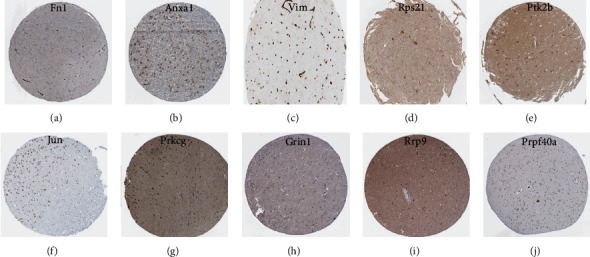
Validation of the hub genes by immunohistochemistry data from the HPA database (hub genes: (a) Fn1, (b) Anxa1, (c) Vim, (d) Rps21, (e) Ptk2b, (f) Jun, (g) Prkcg, (h) Grin1, (i) Rrp9, and (j) Prpf40a).

Human Protein Atlas analysis indicated that the hub genes Fn1, Anxa1, Vim, and Rps21 (upregulated in the DG); Ptk2b, Jun, and Prkcg (downregulated in the DG); Grin1 and Rrp9 (upregulated in the ACC); and Prpf40a (downregulated in the ACC) were expressed in the corresponding regions of the human brain at moderate or high levels (Figure 8).

**Figure 9:**
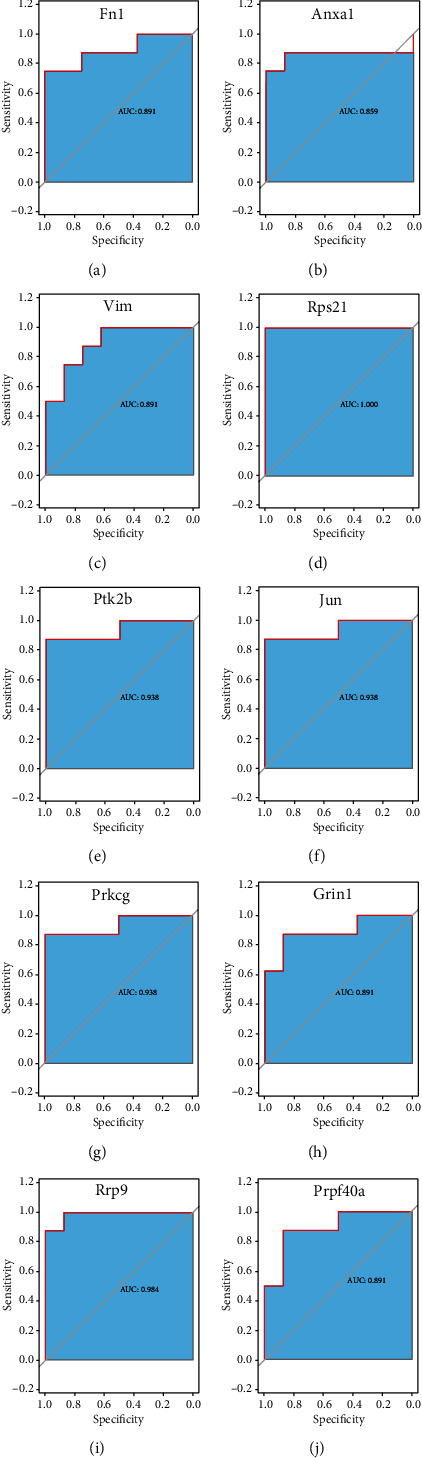
Receiver operating characteristic (ROC) curves and area under the curve (AUC) statistics to evaluate the specificity of differential expression of the hub genes in the UCMS animal model (hub genes: (a) Fn1, (b) Anxa1, (c) Vim, (d) Rps21, (e) Ptk2b, (f) Jun, (g) Prkcg, (h) Grin1, (i) Rrp9, and (j) Prpf40a).

ROC analysis showed that the differential expression of these hub genes, including Fn1, Anxa1, Vim, Rps21, Ptk2b, Jun, Prkcg, Grin1, Rrp9, and Prpf40a, was specific in MDD-like animals (AUC values were greater than 0.85; Fn1: AUC = 0.891, Anxa1: AUC = 0.859, Vim: AUC = 0.891, Rps21: AUC = 1.00, Ptk2b: AUC = 0.938, Jun: AUC = 0.938, Prkcg: AUC = 0.938, Grin1: AUC = 0.891, Rrp9: AUC = 0.984, and Prpf40a: AUC = 0.891) (Figure 9).

## 4. Discussion

Quantitative analysis of the mRNA expression in DG and ACC samples from UCMS-exposed mice and control mice showed that the number of DEGs in the DG was significantly greater than that in the ACC. The number of DEGs with the same level of fold change in the ACC was substantially less than that in the DG; there were only 13 DEGs in the ACC and up to 1071 DEGs with a fold change > 1.5. These results demonstrated that the DG is far more affected than the ACC under the same degree of environmental pressure. To investigate a certain number of DEGs with high fold changes in the DG and ACC, which may be associated with the main pathological changes in UCMS-exposed mice, the DEGs with a fold change > 1.5 in the DG and a fold change > 1.2 in the ACC were selected for subsequent functional analysis. In the functional enrichment analysis, more than a dozen genes upregulated in both the DG and ACC were shown to be involved in the proteolysis biological process. Several studies have indicated that proteolysis modulates synaptic plasticity, which may lead to hippocampal long-term depression (LTD) [43, 44] and to the development of fear memory in the prefrontal cortex [45, 46]. It was also reported that stress could increase LTD and decrease plasticity in the hippocampus [47]. Therefore, proteolysis may be a significant pathological change induced by UCMS in both the DG and ACC. These genes involved in proteolysis may be responsible for the progression of depression. The DG is also affected by other biological processes that regulate neural structure and function, such as cell adhesion, chemical synaptic transmission, and neuroactive ligand-receptor interaction. Some studies have shown that cell adhesion plays a vital role in depression-related behavior [48–50]. It is well documented that MDD is linked to excitation inhibition imbalance due to disrupted synaptic transmission [51]. A multidata source-based prioritization analysis showed that neuroactive ligand-receptor interactions are the core pathways related to MDD [52]. In addition, MDD also involves biological processes that may cause neuronal damage, including the inflammatory response to lipopolysaccharide, calcium ion binding, calmodulin binding, and complement cascades. Consistent with the results of this study, many studies have reported that depression is closely related to neuroinflammation [53, 54]. Excitotoxicity involving aberrant calcium ion binding is a main neuropathological process in various neurodegenerative disorders [55]. Calmodulin activity reportedly regulates group I metabotropic glutamate receptor-mediated signal transduction and synaptic depression [56]. The complement system plays an essential role in synaptic plasticity and cognitive functions [57]. However, the ACC mainly involves biological processes such as metabolic processes, visual learning, long-term memory, and DNA methylation. These actions are thus far linked to only nerve function changes and are not known to be involved in biological processes related to nerve cell damage. Among these processes, consistent with our reports, metabolic disturbances in the prefrontal cortex are known to be involved in various types of depression [58–60]. Visual learning was linked to the development of MDD [61, 62]. Neural anomalies have been shown to characterize depressed individuals during the suppression of long-term memories [63]. DNA methylation is regarded as a key epigenetic mechanism in MDD [64]. Dozens of downregulated genes in the DG were enriched in the biological processes of neuronal repair. These included cell differentiation, multicellular organism development, and nervous system development, which may play essential roles in the advancement of MDD [65]. Some downregulated genes in the DG were shown to involve negative regulation of the neuronal apoptotic process, which is responsible for the progression of MDD [66]. Additionally, some genes downregulated in the DG were shown to be involved in responses to drugs, which has not yet been associated with MDD. However, the ACC mainly involves biological processes such as innate immune responses, cell migration, regulation of stem cell pluripotency, and circadian rhythm function. Among them, innate immune systems alter the pathophysiology of depression [67]. Circadian rhythm disturbances are significant for the development of depression [68]. There is less information on the roles of cell migration and stem cell pluripotency in depression, which may involve neuroplasticity. The downregulation of stem cell pluripotency may underlie the progression of MDD [19].

Comprehensive analysis of the PPI network and modules showed that the hub genes and their corresponding modules in the DG and ACC were different. The modules with upregulated hub genes in the DG mainly involved degradation of the extracellular matrix and positive regulation of behavior. Hippocampal extracellular matrix alterations were shown to be involved in the progression of depression [69]. The aberrant regulation of behavior may directly lead to depressive-like behavior [70, 71]. The modules with upregulated hub genes in the ACC mainly involved responses to anesthetics and visual behavior. Visual learning was previously associated with the development of MDD [61, 62]. There are few reports on the role of responses to anesthetics in depression being associated with the progression of MDD. The modules with downregulated hub genes in the DG mainly involved the positive regulation of smooth muscle contraction and axon extension in axon guidance-related genes. The downregulation of genes associated with the positive regulation of smooth muscle contraction directly affects behavioral control [70, 71]. The downregulation of genes related to axon extension in axon guidance affects neuroplasticity. Many studies have shown that the inhibition of neural plasticity is associated with the pathogenesis of MDD [19]. The modules with downregulated hub genes in the ACC mainly involved signaling by Erb-b2 receptor tyrosine kinase 2 (ERBB2). Furthermore, mGluR1-dependent long-term depression in rodent midbrain dopamine neurons is reportedly regulated by Neuregulin 1/ErbB signaling [72]. Therefore, these modules may be involved in the pathogenesis of MDD.

In the PPI network, upregulated genes in the DG, such as Fn1, Col1a1, Anxa1, Penk, Ptgs2, Cdh1, Timp1, Vim, Rpl30, and Rps21, were identified as hub genes showing the highest node degree and betweenness values. Fn1 is a biomarker of the inflammatory response and is also regarded as an indicator of Alzheimer's disease progression [73, 74]. It has been reported that Col1a1 is an indicator of neurotoxicity [75]. Anxa1 plays a crucial role in chronic corticosterone-induced depression-like behaviors and impairment in hippocampal-dependent memory [76]. Penk knockout mice are resistant to chronic mild stress effects, suggesting that Penk enhances the reactivity to chronic stress [77]. Ptgs2 has been reported to be a vital biomarker of neuroinflammation in Alzheimer's disease [77]. Cdh1 reportedly inhibits the proliferation and activation of oligodendrocyte precursor cells after mechanical stretch injury [78]. Timp1 overexpression damages hippocampal long-term potentiation (LTP). The dysregulation of Timp1 expression may be the basis of abnormal cognitive abilities [79]. The upregulated expression of Vim is an indicator of astrocyte activation and reactive glial hyperplasia in response to injury, ischemia, or neurodegeneration [80]. Therefore, the upregulated expression of these genes in the DG may be responsible for the development of MDD, and these genes may be new therapeutic targets in MDD. In addition, Rpl30 and Rps21 are both ribosomal proteins involved in rRNA processing [81]. These two genes were the upregulated hub genes in the DG that were related to MDD and have not been related to neurological function in the brain. Downregulated genes in the DG, such as Dntt, Ptk2b, Jun, Avp, Slit1, and Sema5a, were identified as hub genes. It has been reported that Ptk2b mediates amyloid-*β*-induced synaptic dysfunction and loss, but its role in MDD has not been reported [82]. It was documented that inhibition of Jun kinase ameliorates depressive-like behaviors and reduces the activation of proinflammatory cytokines induced by central lipopolysaccharide (LPS) infusion [83]. Avp is well known to be involved in the progression of MDD [84, 85]. Therefore, UCMS exposure can activate the expression of genes in the DG that are involved in the pathogenesis of MDD but can also inhibit the expression of genes in the DG that can reduce nerve damage. When UCMS exposure cannot reduce the activities of these genes due to genetic mutations, these genes may lead to the progression of MDD. In addition, Dntt, Slit1, and Sema5a were identified as downregulated hub genes in the DG that were related to MDD, which has not been documented to be associated with neurological function in the brain. Upregulated genes in the ACC, such as Prkcg, Grin1, Syngap1, Rrp9, and Grwd1, were identified as hub genes. Genetic variants in Prkcg may play essential roles in the development of MDD [86, 87]. Grin1 receptor deletion within CRF neurons was shown to enhance fear memory [88]. Increased Grin1 mRNA expression was previously associated with reduced depression-like behavior in a mouse model of neglect [89]. Syngap1 plays critical roles in synaptic development, structure, function, and plasticity in association with neurodevelopmental disorders [90]. Therefore, the increased expression of these genes in the ACC may reduce the occurrence of depressive disorders induced by UCMS. When UCMS exposure cannot increase the activities of these genes due to genetic variants, these genes may lead to the development of MDD. In addition, Rrp9 and Grwd1 were the two upregulated hub genes in the ACC that were related to MDD, which has not been documented to be associated with neurological function in the brain. Downregulated genes in the ACC, such as Pik3r1, Hnrnpc, and Prpf40a, were identified as hub genes. Knockdown of Pik3r1 reportedly inhibits the activity of splenic macrophages associated with hypersplenism [91]. Therefore, Pik3r1 may be associated with neuroinflammation involved in MDD. In addition, Hnrnpc and Prpf40a were the two downregulated hub genes in the ACC related to MDD, which has not been documented to be associated with neurological function in the brain.

In the hub gene-TF regulatory network analysis, which was performed to identify transcription factors that have an important influence on the pathological mechanism of MDD, transcription factors capable of simultaneously regulating the transcriptional expression of more than two hub genes were identified. Identification of these genes will have a significant effect on finding possible new therapeutic targets in MDD. The transcription factor Chd2 was predicted to upregulate the expression of Rpl30 and Rps21 and was previously demonstrated to be necessary for neural circuit development and long-term memory [92]. Zmiz1 was shown to play a key role in neural development in a syndromic neurodevelopmental disorder [93]. Myb was associated with Parkinson's disease [94]. Etv4 was shown to be involved in vesicular glutamate transporter 3 expression and neurite outgrowth of dorsal root ganglion neurons induced by BNDF [95]. Downregulation of Rela in the hippocampus was shown to be involved in preventing major oxidative damage in a chronic model of unpredictable mild stress induced by BDNF [96]. Therefore, the upregulation of Rela in the DG may be involved in the pathogenesis of MDD. The expression of Tcf4 may be significant in the pathomechanism of recurrent depressive disorder [97]. Enhanced expression of Tcf12 in the rat hippocampus was shown to be associated with cognitive function, synaptic plasticity, and pathology [98]. Chd1 depletion robustly enhanced TDP-43-mediated neurodegeneration and promoted the formation of stress granules [99]. Mef2a was shown to play a key role in the differentiation and maturation of rat neural stem cells into neurons [100]. Reducing Mef2a activity was shown to be involved in Parkinson's disease features in model animals [101]. Ubtf was reportedly associated with neurodegeneration in childhood [102]. Mxi1 is known to be essential for neurogenesis in *Xenopus* [103].

## 5. Conclusions

In conclusion, DEG and GO ontology enrichment analyses of the DG and ACC may reveal the molecular mechanism of MDD. Hub genes in the DG, including Fn1, Col1a1, Anxa1, Penk, Ptgs2, Cdh1, Timp1, Vim, Rpl30, Rps21, Dntt, Ptk2b, Jun, Avp, Slit1, and Sema5a, were identified. Hub genes in the ACC, such as Prkcg, Grin1, Syngap1, Rrp9, Grwd1, Pik3r1, Hnrnpc, and Prpf40a, were identified. The transcription factor genes Chd2, Zmiz1, Myb, Etv4, Rela, Tcf4, Tcf12, Chd1, Mef2a, Ubtf, and Mxi1 may regulate more than two hub genes in the DG and ACC. Our findings provide clues for further exploring molecular mechanisms and developing new therapeutic approaches to MDD.

## Data Availability

The datasets supporting the conclusions of this article are available in the Gene Expression Omnibus (GEO) (https://www.ncbi.nlm.nih.gov/geo/) repository under accession number GSE84183 (https://www.ncbi.nlm.nih.gov/geo/query/acc.cgi?acc=GSE84183).

## Abbreviations

MDD: Major depressive disorder
DG: Dentate gyrus
ACC: Anterior cingulate cortex
DEGs: Differentially expressed genes
HPA: Human Protein Atlas
ROC: Receiver operating characteristic
PPI: Protein-protein interaction
CRF: Corticotrophin-releasing factor
NMDA: N-Methyl-D-aspartate
SSRI: Selective serotonin reuptake inhibitor
GEO: Gene Expression Omnibus
UCMS: Unpredictable chronic mild stress
GO: Gene Ontology
BP: Biological process
MF: Molecular function
CC: Cellular component
KEGG: Kyoto Encyclopedia of Genes and Genomes
LTD: Long-term depression
ERBB2: Erb-b2 receptor tyrosine kinase 2
LTP: Long-term potentiation
LPS: Lipopolysaccharide.

## Supplementary Materials

Supplementary Materials Figure S1: box plots of the gene expression data after normalization. Horizontal axis represents the sample symbol, and the vertical axis represents the gene expression values. The black line in the box plot represents the median value of gene expression. (A1–A16 = DG samples; B1–B16 = ACC samples). Figure S2: principal component analysis (PCA): (A) the overall distribution of UCMS-induced differentially expressed genes in DG; (B) the overall distribution of UCMS-induced differentially expressed genes in ACC. Figure S3: principal component analysis (PCA) of differentially expressed genes in DG and ACC. Figure S4: heat map of the common UCMS-induced DEGs in DG and ACC. Figure S5: regression diagrams for upregulated genes in DG: (A) betweenness centrality; (B) node degree. Figure S6: regression diagrams for downregulated genes in DG: (A) betweenness centrality; (B) node degree. Figure S7: regression diagrams for upregulated genes in ACC: (A) betweenness centrality; (B) node degree. Figure S8: regression diagrams for downregulated genes in ACC: (A) betweenness centrality; (B) node degree. Table S1: the statistical metrics for key differentially expressed genes (DEGs with fold change > 1.5) in DG. Table S2: the statistical metrics for key differentially expressed genes (DEGs with fold change > 1.2) in ACC. Table S3: the enriched GO terms of the key differentially expressed genes in DG and ACC. Table S4: the enriched KEGG pathways of the key differentially expressed genes in DG and ACC. Table S5: topology table for the key differentially expressed genes in DG and ACC.
