# Supplementary material for: Analysis of Differentially Expressed Genes in the Dentate Gyrus and Anterior Cingulate Cortex in a Mouse Model of Depression
Source: Biomed Res Int. 2021 Feb 11;2021:5013565. doi: 10.1155/2021/5013565 (PMC7892236; doi:10.1155/2021/5013565)
Supplement: Supplementary Materials — Figure S1: box plots of the gene expression data after normalization. Horizontal axis represents the sample symbol, and the vertical axis represents the gene expression values. The black line in the box plot represents the median value of gene expression. (A1–A16 = DG samples; B1–B16 = ACC samples). Figure S2: principal component analysis (PCA): (A) the overall distribution of UCMS-induced differentially expressed genes in DG; (B) the overall distribution of UCMS-induced differentially expressed genes in ACC. Figure S3: principal component analysis (PCA) of differentially expressed genes in DG and ACC. Figure S4: heat map of the common UCMS-induced DEGs in DG and ACC. Figure S5: regression diagrams for upregulated genes in DG: (A) betweenness centrality; (B) node degree. Figure S6: regression diagrams for downregulated genes in DG: (A) betweenness centrality; (B) node degree. Figure S7: regression diagrams for upregulated genes in ACC: (A) betweenness centrality; (B) node degree. Figure S8: regression diagrams for downregulated genes in ACC: (A) betweenness centrality; (B) node degree. Table S1: the statistical metrics for key differentially expressed genes (DEGs with fold change > 1.5) in DG. Table S2: the statistical metrics for key differentially expressed genes (DEGs with fold change > 1.2) in ACC. Table S3: the enriched GO terms of the key differentially expressed genes in DG and ACC. Table S4: the enriched KEGG pathways of the key differentially expressed genes in DG and ACC. Table S5: topology table for the key differentially expressed genes in DG and ACC. [file 5013565.f1.docx]

**Supplementary**

**
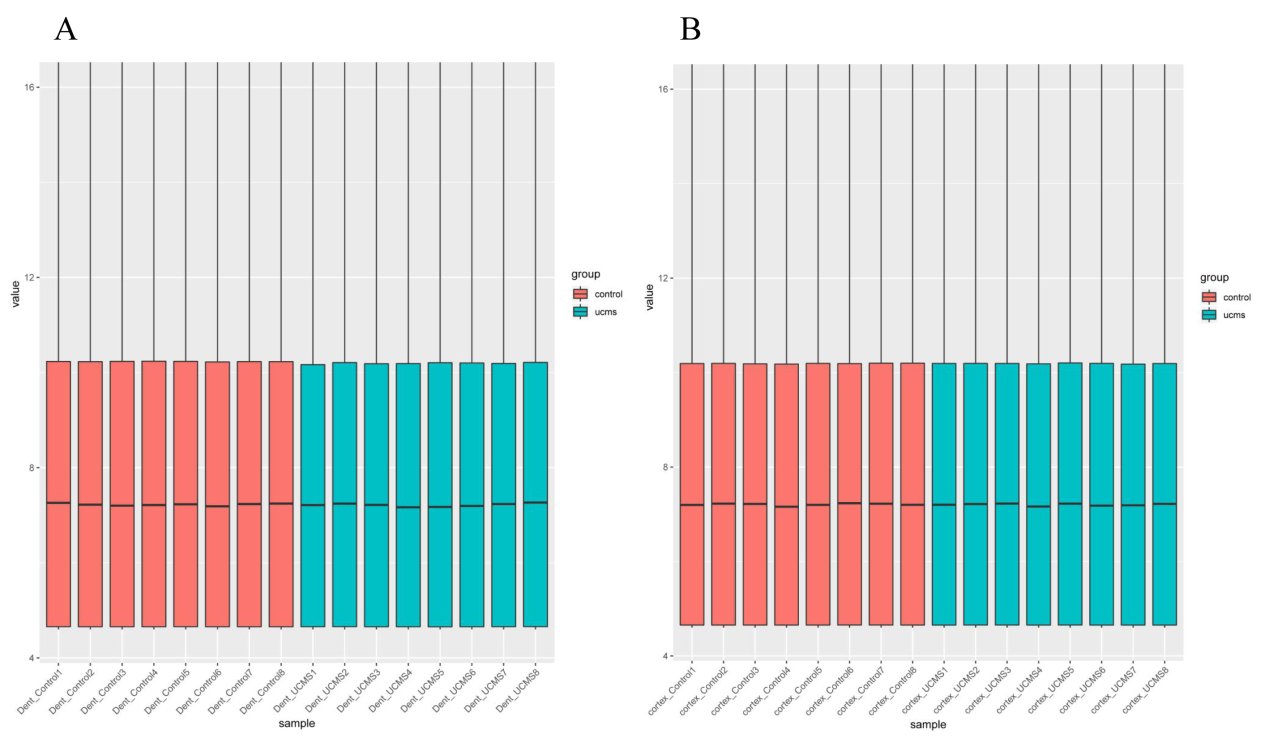
**

**Figure S1.** Box plots of the gene expression data after normalization. Horizontal axis represents the sample symbol and the vertical axis represents the gene expression values. The black line in the box plot represents the median value of gene expression. (A1–A16 =DG samples; B1–B16 = ACC samples)

**A:**


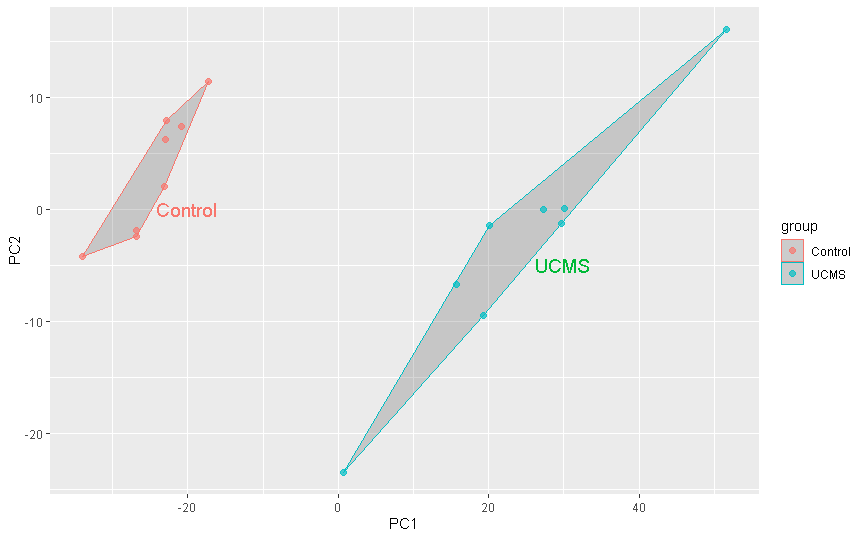


**B:**


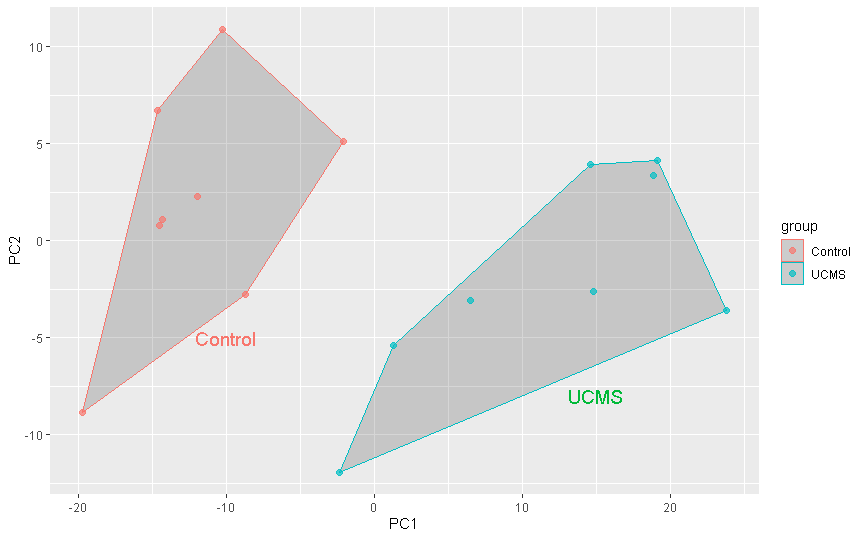


**Figure S2.**Principal component analysis (PCA), (A): the overall distribution of UCMS-induced differentially expressed genes in DG; (B): the overall distribution of UCMS-induced differentially expressed genes in ACC.


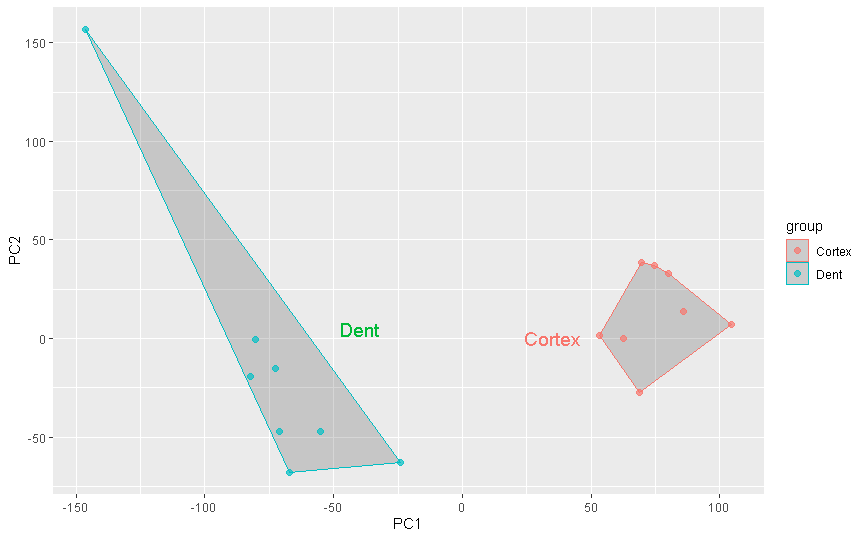


**Figure S3.**Principal component analysis (PCA) of differentially expressed genes in DG and ACC.


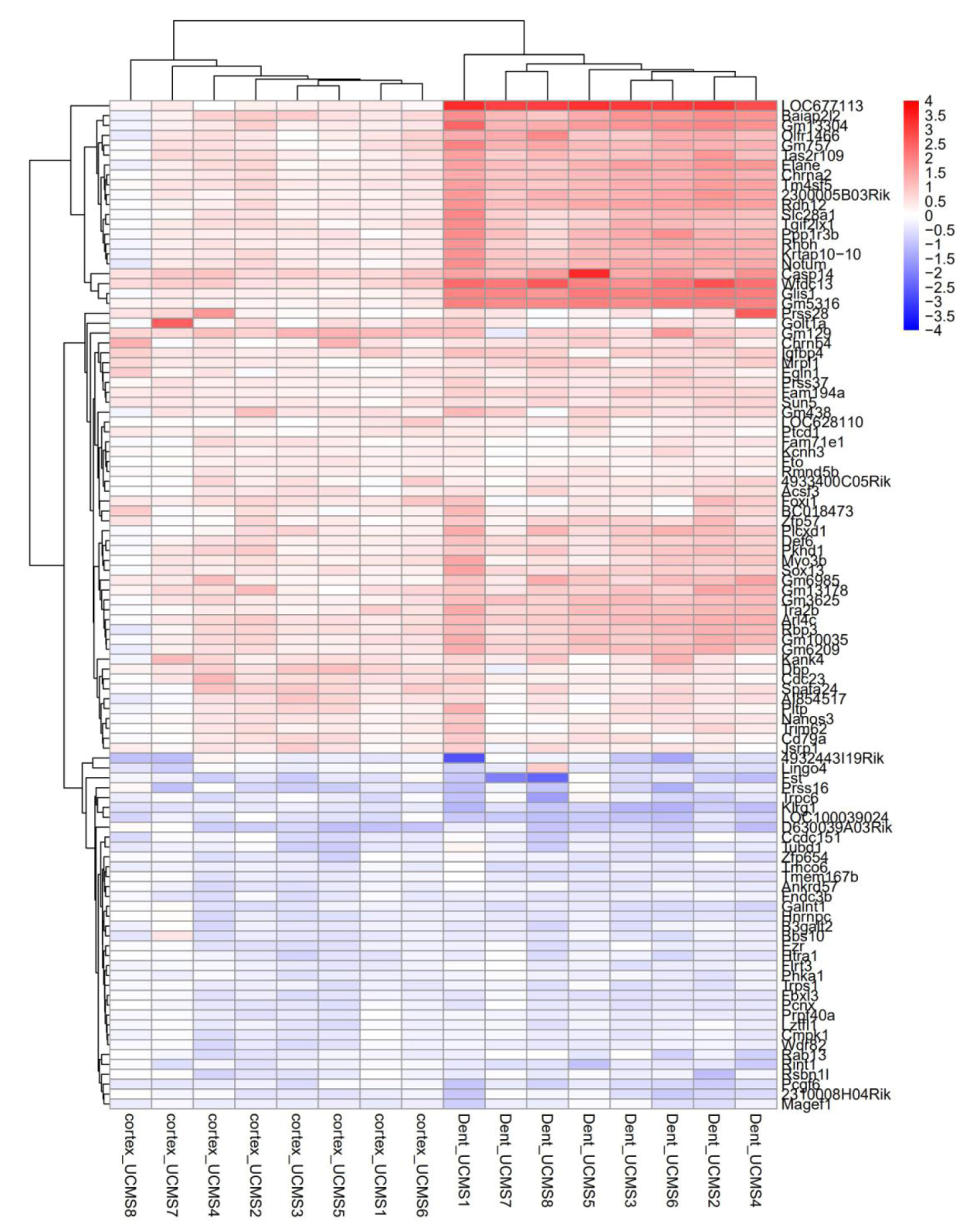


**Figure S4.** Heat map of the common UCMS-induced DEGs in DG and ACC.


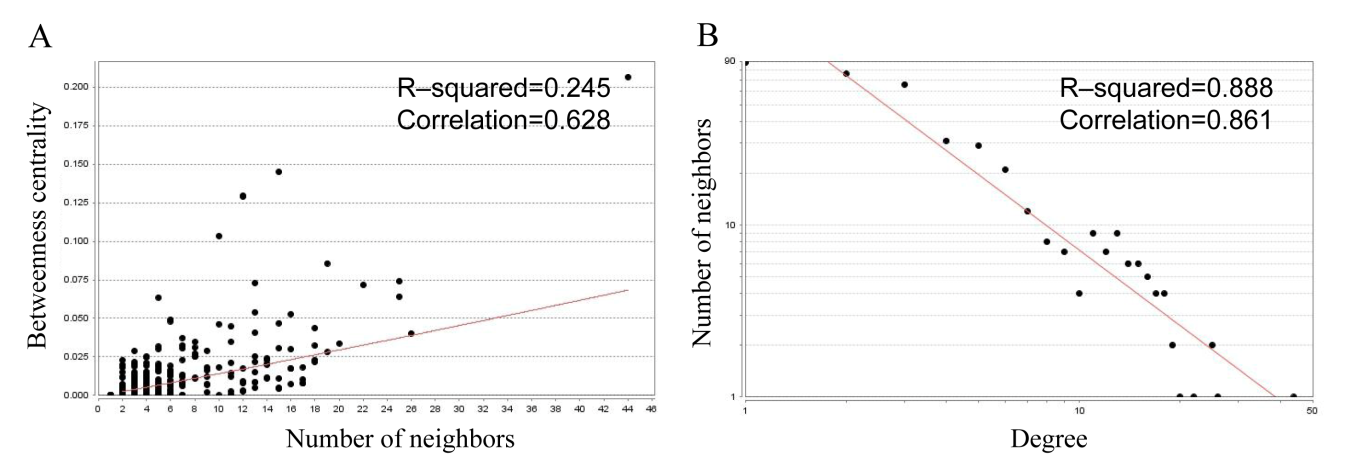


**Figure S5.** Regression diagrams for up-regulated genes in DG. (A) Betweenness centrality; (B) Node degree;


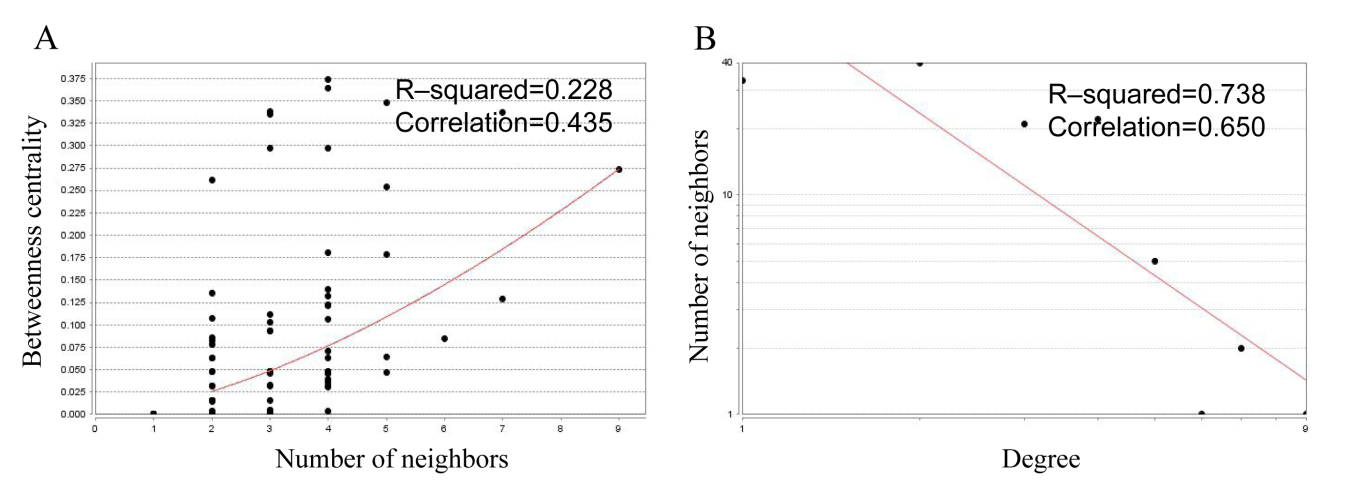


**Figure S6.** Regression diagrams for down-regulated genes in DG. (A) Betweenness centrality; (B) Node degree;


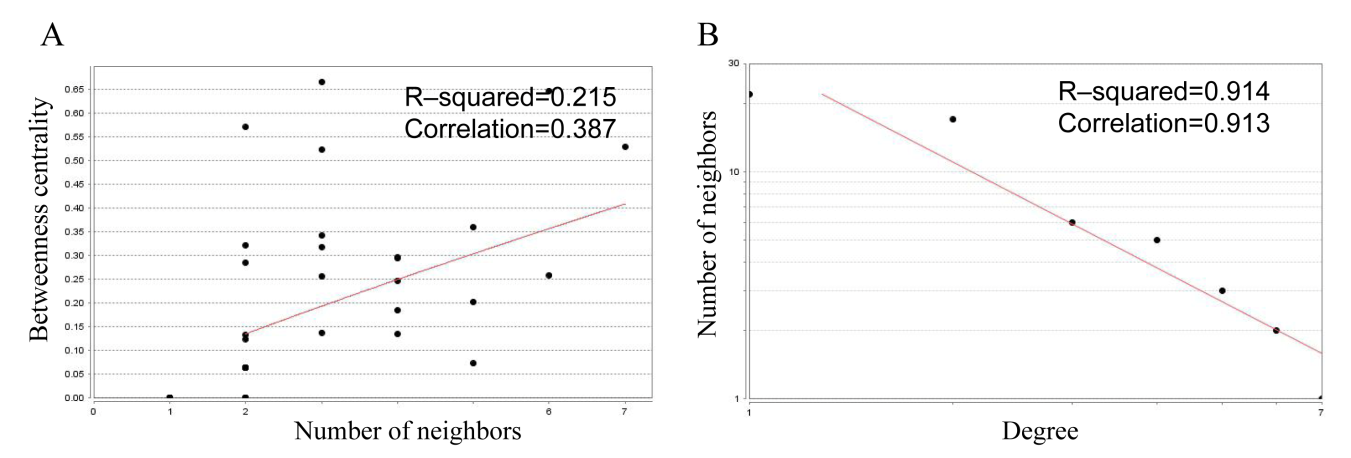


**Figure S7.** Regression diagrams for up-regulated genes in ACC. (A) Betweenness centrality; (B) Node degree;


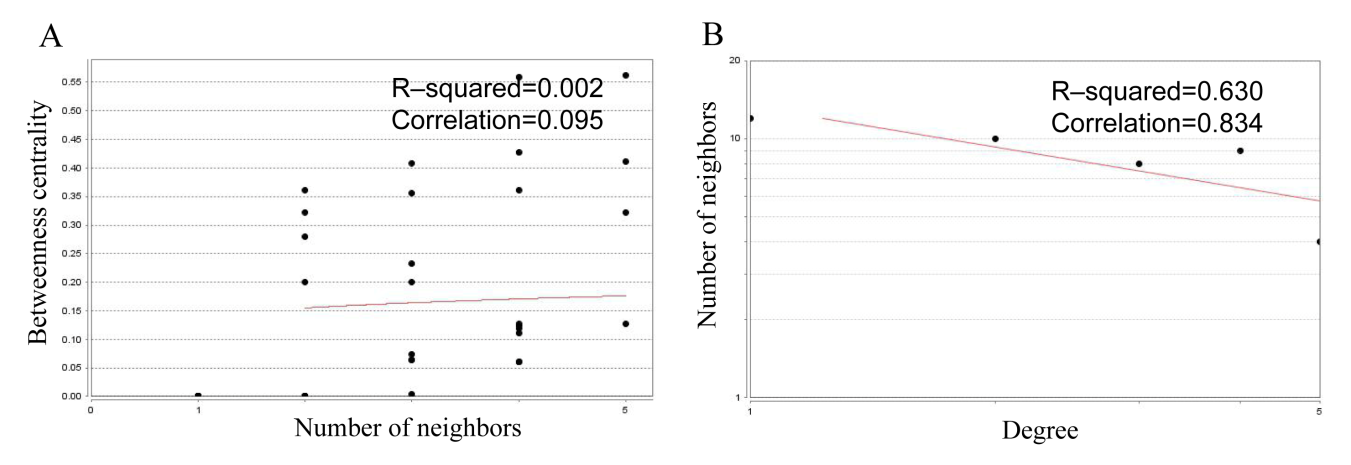


**Figure S8.** Regression diagrams for down-regulated genes in ACC. (A) Betweenness centrality; (B) Node degree;

**Table S1. The statistical metrics for key differentially expressed genes (DEGs with Fold Change> 1.5 ) in DG**

| **NO** | **Gene** | **logFC** | **AveExpr** | **t** | **P.Value** | **adj.P.Val** | **B** |
| --- | --- | --- | --- | --- | --- | --- | --- |
| 1 | Gm8618 | 3.43513 | 10.8199 | 16.88319 | 2.99E-11 | 2.53E-08 | 16.10836 |
| 2 | LOC677113 | 3.090816 | 9.038464 | 19.42751 | 3.88E-12 | 4.50E-09 | 18.0035 |
| 3 | Aff4 | 2.535322 | 6.651112 | 10.63729 | 1.96E-08 | 2.82E-06 | 9.771169 |
| 4 | Gpr88 | 2.453237 | 7.429453 | 3.701152 | 0.002096 | 0.01351 | -1.96665 |
| 5 | Vax2 | 2.263439 | 6.67879 | 12.30502 | 2.66E-09 | 6.18E-07 | 11.75915 |
| 6 | Hcn1 | 2.25284 | 7.085908 | 8.639171 | 3.01E-07 | 1.93E-05 | 7.009849 |
| 7 | Wfdc13 | 2.246848 | 8.327347 | 8.038418 | 7.48E-07 | 3.73E-05 | 6.085984 |
| 8 | Gm6003 | 2.086367 | 7.428475 | 27.23731 | 2.64E-14 | 4.31E-10 | 22.31248 |
| 9 | Ephx3 | 1.9945 | 6.644913 | 14.42517 | 2.86E-10 | 1.22E-07 | 13.94912 |
| 10 | Glis1 | 1.979596 | 8.827554 | 18.68809 | 6.84E-12 | 7.18E-09 | 17.48386 |
| 11 | Ctxn3 | 1.974239 | 6.065595 | 4.54059 | 0.000379 | 0.003668 | -0.25248 |
| 12 | Wnt6 | 1.974225 | 6.049134 | 3.909561 | 0.001365 | 0.009789 | -1.53942 |
| 13 | Gm5316 | 1.971381 | 8.70424 | 16.34318 | 4.78E-11 | 3.22E-08 | 15.66439 |
| 14 | Gm6987 | 1.969291 | 7.200704 | 13.7986 | 5.36E-10 | 1.87E-07 | 13.33691 |
| 15 | Usp8 | 1.958544 | 7.499895 | 15.55257 | 9.75E-11 | 5.37E-08 | 14.98466 |
| 16 | Foxc2 | 1.932103 | 5.605441 | 4.049712 | 0.001025 | 0.00785 | -1.25225 |
| 17 | Hs3st6 | 1.913404 | 6.248127 | 4.173714 | 0.000796 | 0.006467 | -0.99858 |
| 18 | Syt10 | 1.898254 | 12.71549 | 8.593868 | 3.22E-07 | 1.99E-05 | 6.941787 |
| 19 | Omd | 1.887694 | 5.695138 | 3.676041 | 0.002207 | 0.014119 | -2.01808 |
| 20 | Vmn1r65 | 1.829821 | 8.658843 | 8.361231 | 4.56E-07 | 2.59E-05 | 6.588192 |
| 21 | Spef2 | 1.818163 | 12.18308 | 9.695029 | 6.75E-08 | 6.88E-06 | 8.525465 |
| 22 | Cyp17a1 | 1.812646 | 9.958427 | 22.73725 | 3.84E-13 | 1.01E-09 | 20.06505 |
| 23 | B020031M17Rik | 1.791191 | 9.490817 | 5.868335 | 2.94E-05 | 0.000556 | 2.342401 |
| 24 | Insrr | 1.776181 | 5.514303 | 4.318258 | 0.000593 | 0.005153 | -0.7037 |
| 25 | Colec12 | 1.732703 | 5.831213 | 4.374252 | 0.00053 | 0.00471 | -0.58976 |
| 26 | Slc6a12 | 1.713655 | 6.4601 | 2.927158 | 0.010302 | 0.046641 | -3.53165 |
| 27 | Leo1 | 1.713506 | 8.145917 | 23.66263 | 2.13E-13 | 9.39E-10 | 20.57391 |
| 28 | Hist1h4h | 1.711885 | 5.955035 | 11.07013 | 1.14E-08 | 1.90E-06 | 10.31243 |
| 29 | Slc13a4 | 1.702796 | 10.39591 | 3.428852 | 0.003675 | 0.020927 | -2.52313 |
| 30 | Gm13304 | 1.701963 | 11.53377 | 13.18014 | 1.02E-09 | 2.96E-07 | 12.70474 |
| 31 | Rpl26-ps2 | 1.701398 | 9.285267 | 23.21267 | 2.83E-13 | 1.01E-09 | 20.32981 |
| 32 | Casp14 | 1.683721 | 5.505777 | 6.845267 | 5.21E-06 | 0.000157 | 4.108309 |
| 33 | Slc22a7 | 1.680101 | 6.238415 | 3.765679 | 0.001835 | 0.012176 | -1.83441 |
| 34 | Gm15032 | 1.657621 | 9.981705 | 22.9538 | 3.34E-13 | 1.01E-09 | 20.18654 |
| 35 | Gm5670 | 1.657575 | 11.56195 | 15.26704 | 1.27E-10 | 6.67E-08 | 14.73003 |
| 36 | Gm9372 | 1.636468 | 5.908899 | 14.35953 | 3.05E-10 | 1.24E-07 | 13.88627 |
| 37 | Crabp2 | 1.635697 | 9.64092 | 3.982835 | 0.001175 | 0.008716 | -1.38924 |
| 38 | Mpzl2 | 1.619606 | 6.515039 | 3.420795 | 0.003736 | 0.021205 | -2.53954 |
| 39 | Rapsn | 1.602304 | 12.8554 | 19.76374 | 3.02E-12 | 3.70E-09 | 18.23205 |
| 40 | Gm5481 | 1.5957 | 11.93354 | 21.2804 | 1.02E-12 | 1.87E-09 | 19.20712 |
| 41 | LOC546711 | 1.592463 | 8.530466 | 16.90228 | 2.94E-11 | 2.53E-08 | 16.12377 |
| 42 | Sphk1 | 1.591614 | 7.95315 | 3.47363 | 0.003351 | 0.019499 | -2.43188 |
| 43 | Dapl1 | 1.59094 | 5.457685 | 3.113748 | 0.007032 | 0.034784 | -3.16031 |
| 44 | Aebp1 | 1.568385 | 11.13556 | 3.772125 | 0.001811 | 0.012055 | -1.82119 |
| 45 | Serpind1 | 1.563643 | 7.707049 | 3.343466 | 0.004382 | 0.023967 | -2.69674 |
| 46 | Gm5590 | 1.559421 | 5.487451 | 9.516148 | 8.62E-08 | 8.21E-06 | 8.277941 |
| 47 | Lamc2 | 1.528031 | 8.987039 | 3.583649 | 0.00267 | 0.016399 | -2.20718 |
| 48 | Gm6919 | 1.5247 | 11.35635 | 13.77572 | 5.48E-10 | 1.87E-07 | 13.31403 |
| 49 | H2-Eb1 | 1.522277 | 7.551754 | 3.65942 | 0.002284 | 0.014498 | -2.05212 |
| 50 | Capn11 | 1.514864 | 8.458381 | 9.406946 | 1.00E-07 | 9.00E-06 | 8.125027 |
| 51 | Baiap2l2 | 1.511102 | 9.093143 | 9.820911 | 5.69E-08 | 6.24E-06 | 8.697479 |
| 52 | Rps21 | 1.492626 | 11.43852 | 9.167351 | 1.40E-07 | 1.12E-05 | 7.78464 |
| 53 | Nos2 | 1.491365 | 6.085116 | 11.6305 | 5.80E-09 | 1.14E-06 | 10.98617 |
| 54 | Gm10081 | 1.490366 | 5.489934 | 5.41035 | 6.93E-05 | 0.001023 | 1.470323 |
| 55 | Dnase2a | 1.4878 | 11.59295 | 6.930235 | 4.51E-06 | 0.000142 | 4.255632 |
| 56 | Cyp2b10 | 1.487296 | 5.383717 | 3.731586 | 0.001968 | 0.012854 | -1.90428 |
| 57 | Kcnj13 | 1.483796 | 6.785819 | 3.138203 | 0.006687 | 0.033457 | -3.11126 |
| 58 | Gm5215 | 1.477252 | 9.421904 | 8.366681 | 4.53E-07 | 2.58E-05 | 6.596556 |
| 59 | Cbln1 | 1.470988 | 6.726413 | 4.774207 | 0.000238 | 0.002589 | 0.217627 |
| 60 | Slc22a6 | 1.462404 | 8.697698 | 3.709314 | 0.002061 | 0.013326 | -1.94992 |
| 61 | Bglap2 | 1.458103 | 6.011532 | 2.960355 | 0.009627 | 0.044332 | -3.466 |
| 62 | Myh2 | 1.456107 | 5.497764 | 4.330486 | 0.000579 | 0.005065 | -0.6788 |
| 63 | Ccdc147 | 1.443484 | 5.689309 | 4.93471 | 0.000174 | 0.002059 | 0.537757 |
| 64 | Tshz2 | 1.439453 | 8.932705 | 2.912403 | 0.010617 | 0.047761 | -3.56076 |
| 65 | Chat | 1.429603 | 5.632987 | 7.539484 | 1.65E-06 | 6.65E-05 | 5.282639 |
| 66 | Rdh12 | 1.428817 | 12.18102 | 12.36226 | 2.50E-09 | 5.92E-07 | 11.82293 |
| 67 | Thbd | 1.421856 | 7.482912 | 4.215572 | 0.000731 | 0.006081 | -0.91309 |
| 68 | Olfr1368 | 1.417717 | 8.794889 | 12.3476 | 2.54E-09 | 5.96E-07 | 11.80662 |
| 69 | Fmod | 1.405707 | 7.582504 | 2.86914 | 0.011594 | 0.051179 | -3.64589 |
| 70 | Lmtk2 | 1.405643 | 8.033998 | 15.39476 | 1.13E-10 | 6.07E-08 | 14.84454 |
| 71 | LOC386400 | 1.402264 | 5.743265 | 12.56346 | 2.00E-09 | 5.00E-07 | 12.04491 |
| 72 | Vmn1r70 | 1.398112 | 6.360028 | 15.0068 | 1.63E-10 | 8.14E-08 | 14.49352 |
| 73 | Ubr2 | 1.39437 | 6.208685 | 13.62428 | 6.41E-10 | 2.02E-07 | 13.16161 |
| 74 | Aldh1a2 | 1.390238 | 9.241322 | 3.059937 | 0.007852 | 0.037845 | -3.26796 |
| 75 | Elane | 1.378396 | 7.876118 | 11.49429 | 6.82E-09 | 1.27E-06 | 10.82511 |
| 76 | Catsper4 | 1.377006 | 5.491199 | 3.45632 | 0.003472 | 0.020018 | -2.46717 |
| 77 | Foxd2 | 1.36562 | 5.530454 | 3.523679 | 0.003022 | 0.018034 | -2.32974 |
| 78 | Secisbp2l | 1.361359 | 6.683791 | 9.552147 | 8.20E-08 | 7.93E-06 | 8.328048 |
| 79 | Lalba | 1.359452 | 6.861961 | 5.325247 | 8.15E-05 | 0.001156 | 1.305376 |
| 80 | Rpl34-ps1 | 1.352467 | 12.97894 | 14.8662 | 1.86E-10 | 8.72E-08 | 14.36395 |
| 81 | Spatc1 | 1.352247 | 5.365346 | 3.732357 | 0.001965 | 0.012849 | -1.9027 |
| 82 | Gm757 | 1.337191 | 5.820014 | 9.899461 | 5.12E-08 | 5.79E-06 | 8.80392 |
| 83 | Ctgf | 1.334765 | 10.3597 | 4.009823 | 0.001112 | 0.008357 | -1.33394 |
| 84 | 2300005B03Rik | 1.334054 | 7.91245 | 13.35995 | 8.44E-10 | 2.55E-07 | 12.89151 |
| 85 | Bmp3 | 1.329957 | 5.798956 | 3.5721 | 0.002735 | 0.016699 | -2.2308 |
| 86 | Mettl7a2 | 1.328042 | 13.6655 | 5.671727 | 4.24E-05 | 0.000721 | 1.971329 |
| 87 | BC049265 | 1.312936 | 5.511814 | 9.074386 | 1.60E-07 | 1.22E-05 | 7.65073 |
| 88 | Atp6ap1l | 1.312692 | 8.478162 | 3.155792 | 0.00645 | 0.032536 | -3.07593 |
| 89 | Gm4832 | 1.309012 | 12.90016 | 21.68423 | 7.73E-13 | 1.70E-09 | 19.45227 |
| 90 | Gm6272 | 1.305975 | 12.56542 | 10.88772 | 1.43E-08 | 2.20E-06 | 10.08661 |
| 91 | Notum | 1.305698 | 10.36708 | 12.51793 | 2.10E-09 | 5.20E-07 | 11.99497 |
| 92 | Grb7 | 1.303795 | 7.685991 | 3.301618 | 0.004777 | 0.025632 | -2.78161 |
| 93 | Lypd2 | 1.302795 | 5.42334 | 4.591967 | 0.000342 | 0.003392 | -0.14871 |
| 94 | Trp53bp2 | 1.297241 | 5.416652 | 11.03473 | 1.19E-08 | 1.94E-06 | 10.26887 |
| 95 | Tm4sf5 | 1.295961 | 7.77373 | 10.78667 | 1.62E-08 | 2.43E-06 | 9.960094 |
| 96 | Ppp1r3b | 1.293104 | 9.890864 | 7.880182 | 9.57E-07 | 4.52E-05 | 5.834815 |
| 97 | Rtp2 | 1.291572 | 5.462763 | 4.766185 | 0.000242 | 0.002623 | 0.201562 |
| 98 | Aldh3a1 | 1.288055 | 8.381512 | 5.477448 | 6.10E-05 | 0.000932 | 1.599752 |
| 99 | Pmch | 1.278169 | 5.499843 | 4.210664 | 0.000738 | 0.006123 | -0.92311 |
| 100 | Cobl | 1.276866 | 9.071499 | 3.240389 | 0.00542 | 0.028277 | -2.90547 |
| 101 | Asgr1 | 1.275329 | 5.624277 | 3.156102 | 0.006446 | 0.032522 | -3.0753 |
| 102 | Iqcg | 1.264799 | 7.406649 | 6.279091 | 1.40E-05 | 0.000323 | 3.100935 |
| 103 | Cyp2f2 | 1.264761 | 5.288468 | 3.281161 | 0.004983 | 0.026546 | -2.82303 |
| 104 | Fgfbp1 | 1.264614 | 5.773853 | 2.98507 | 0.009153 | 0.042684 | -3.417 |
| 105 | Olfr1466 | 1.262584 | 9.234935 | 4.364818 | 0.00054 | 0.004787 | -0.60895 |
| 106 | Prm1 | 1.262362 | 5.849397 | 6.27155 | 1.42E-05 | 0.000326 | 3.087218 |
| 107 | Olfr1415 | 1.261396 | 5.762602 | 5.68847 | 4.11E-05 | 0.000706 | 2.003126 |
| 108 | Cbln4 | 1.257746 | 7.412603 | 3.457623 | 0.003463 | 0.019979 | -2.46452 |
| 109 | Tnnt2 | 1.256474 | 8.055327 | 4.949307 | 0.000169 | 0.00202 | 0.566745 |
| 110 | Ckap4 | 1.239614 | 6.904862 | 3.022496 | 0.008478 | 0.040201 | -3.34261 |
| 111 | Gm5801 | 1.221807 | 7.886915 | 11.6209 | 5.87E-09 | 1.14E-06 | 10.97488 |
| 112 | Gm14816 | 1.216996 | 10.16508 | 6.565178 | 8.44E-06 | 0.000222 | 3.61554 |
| 113 | Chrna2 | 1.216177 | 6.897716 | 13.8349 | 5.16E-10 | 1.87E-07 | 13.37314 |
| 114 | Cplx3 | 1.215996 | 7.756599 | 4.248191 | 0.000684 | 0.005765 | -0.84652 |
| 115 | Ddx55 | 1.212771 | 7.156072 | 14.66866 | 2.25E-10 | 1.01E-07 | 14.17974 |
| 116 | Cd7 | 1.207532 | 5.643974 | 4.038884 | 0.001048 | 0.007975 | -1.27442 |
| 117 | Krtap10-10 | 1.204212 | 8.381931 | 11.99396 | 3.80E-09 | 8.37E-07 | 11.40767 |
| 118 | Mc3r | 1.198162 | 5.392097 | 4.681388 | 0.000286 | 0.002983 | 0.031398 |
| 119 | Rbp4 | 1.197228 | 11.15648 | 4.009685 | 0.001112 | 0.008357 | -1.33423 |
| 120 | Rhoh | 1.196422 | 8.122405 | 9.168603 | 1.40E-07 | 1.12E-05 | 7.786436 |
| 121 | Wfs1 | 1.186641 | 13.21165 | 3.394938 | 0.003941 | 0.022096 | -2.59216 |
| 122 | Anxa1 | 1.176682 | 6.706172 | 3.208127 | 0.005792 | 0.029872 | -2.97057 |
| 123 | Dtl | 1.176007 | 6.314818 | 3.683638 | 0.002173 | 0.013922 | -2.00252 |
| 124 | Tas2r109 | 1.1753 | 6.717691 | 6.415785 | 1.10E-05 | 0.000271 | 3.348234 |
| 125 | Olfr1350 | 1.17214 | 5.225844 | 14.97274 | 1.68E-10 | 8.23E-08 | 14.46225 |
| 126 | Arhgap1 | 1.171479 | 10.82971 | 9.843812 | 5.52E-08 | 6.08E-06 | 8.728583 |
| 127 | C2 | 1.171408 | 8.200062 | 3.391035 | 0.003973 | 0.022229 | -2.60009 |
| 128 | Slfn9 | 1.166914 | 7.055768 | 6.046825 | 2.12E-05 | 0.000441 | 2.674832 |
| 129 | Pinx1 | 1.166249 | 10.38271 | 6.763384 | 5.99E-06 | 0.000173 | 3.965383 |
| 130 | Rpl4 | 1.164887 | 13.99203 | 15.09725 | 1.49E-10 | 7.65E-08 | 14.57621 |
| 131 | Ptgds | 1.164589 | 16.74045 | 3.894599 | 0.001408 | 0.010009 | -1.5701 |
| 132 | Foxd1 | 1.161446 | 5.374022 | 3.040209 | 0.008176 | 0.039072 | -3.30732 |
| 133 | Gm11213 | 1.160189 | 7.539562 | 9.025181 | 1.72E-07 | 1.30E-05 | 7.579434 |
| 134 | Myo1h | 1.159022 | 10.69708 | 4.629249 | 0.000318 | 0.00322 | -0.07354 |
| 135 | Lin28a | 1.151346 | 5.53166 | 8.348024 | 4.66E-07 | 2.63E-05 | 6.567911 |
| 136 | Col1a1 | 1.149949 | 8.508392 | 2.53659 | 0.022642 | 0.086273 | -4.28615 |
| 137 | Cd74 | 1.148688 | 6.718829 | 2.502436 | 0.02423 | 0.09083 | -4.35024 |
| 138 | Gm4593 | 1.146297 | 7.788521 | 7.607243 | 1.48E-06 | 6.13E-05 | 5.393707 |
| 139 | Mmel1 | 1.140564 | 6.743436 | 3.311447 | 0.004682 | 0.025245 | -2.76169 |
| 140 | Tox4 | 1.139139 | 10.59944 | 11.483 | 6.91E-09 | 1.28E-06 | 10.81169 |
| 141 | Rpl12-ps1 | 1.138292 | 12.50252 | 9.599863 | 7.68E-08 | 7.52E-06 | 8.394234 |
| 142 | Tnnc2 | 1.134849 | 5.485834 | 3.38271 | 0.004042 | 0.022511 | -2.61702 |
| 143 | Prss53 | 1.130652 | 10.47429 | 7.843954 | 1.01E-06 | 4.72E-05 | 5.776841 |
| 144 | D0H4S114 | 1.12951 | 10.69832 | 5.091123 | 0.000128 | 0.00162 | 0.847217 |
| 145 | Col9a2 | 1.1283 | 9.312536 | 3.771341 | 0.001814 | 0.01207 | -1.8228 |
| 146 | Clec10a | 1.126326 | 5.740832 | 4.425691 | 0.000478 | 0.004356 | -0.48526 |
| 147 | LOC100505291 | 1.125876 | 10.80474 | 14.28691 | 3.27E-10 | 1.29E-07 | 13.81639 |
| 148 | 1700019O17Rik | 1.125738 | 9.932287 | 10.01153 | 4.41E-08 | 5.16E-06 | 8.954605 |
| 149 | Cd244 | 1.125736 | 8.16128 | 6.495148 | 9.54E-06 | 0.000245 | 3.490624 |
| 150 | Lyz1 | 1.120164 | 7.673453 | 4.253683 | 0.000676 | 0.005714 | -0.83531 |
| 151 | Gm11449 | 1.114742 | 9.43791 | 7.202271 | 2.86E-06 | 0.000101 | 4.720542 |
| 152 | Klhdc7a | 1.114265 | 10.77248 | 13.77774 | 5.47E-10 | 1.87E-07 | 13.31606 |
| 153 | Satb2 | 1.111986 | 6.035673 | 2.980312 | 0.009243 | 0.04301 | -3.42644 |
| 154 | Drd1a | 1.111728 | 6.740871 | 4.502908 | 0.000409 | 0.003872 | -0.32872 |
| 155 | Baiap2l1 | 1.11047 | 10.05463 | 3.438457 | 0.003603 | 0.020607 | -2.50357 |
| 156 | 1700022A21Rik | 1.110437 | 7.430517 | 8.625597 | 3.07E-07 | 1.95E-05 | 6.989483 |
| 157 | Rec8 | 1.109758 | 7.42959 | 11.04637 | 1.17E-08 | 1.94E-06 | 10.2832 |
| 158 | COX3 | 1.107093 | 17.05672 | 20.00014 | 2.54E-12 | 3.29E-09 | 18.38996 |
| 159 | Igfbp6 | 1.105625 | 12.51354 | 3.96468 | 0.001219 | 0.008976 | -1.42644 |
| 160 | Prosapip1 | 1.104885 | 11.68765 | 3.488869 | 0.003247 | 0.019031 | -2.40079 |
| 161 | 2900073C17Rik | 1.10379 | 5.9846 | 3.38005 | 0.004064 | 0.022601 | -2.62243 |
| 162 | Il13ra2 | 1.100105 | 6.779264 | 5.991515 | 2.35E-05 | 0.000474 | 2.572277 |
| 163 | 9230110C19Rik | 1.098846 | 12.44784 | 4.470542 | 0.000437 | 0.004075 | -0.39429 |
| 164 | Slc16a3 | 1.098299 | 8.565753 | 3.608114 | 0.002539 | 0.015757 | -2.15714 |
| 165 | Timp1 | 1.09644 | 5.347708 | 2.803653 | 0.013243 | 0.05666 | -3.77404 |
| 166 | Camk2n1 | 1.093414 | 12.39085 | 8.999673 | 1.78E-07 | 1.33E-05 | 7.542359 |
| 167 | Utf1 | 1.089802 | 6.725358 | 8.278824 | 5.17E-07 | 2.86E-05 | 6.461277 |
| 168 | Bglap-rs1 | 1.081038 | 6.101276 | 3.074388 | 0.007623 | 0.037029 | -3.23909 |
| 169 | Gm7634 | 1.076364 | 13.2208 | 11.37466 | 7.87E-09 | 1.42E-06 | 10.68225 |
| 170 | Siglece | 1.075511 | 8.897492 | 8.492963 | 3.75E-07 | 2.24E-05 | 6.789263 |
| 171 | Casp1 | 1.07498 | 8.464336 | 3.760448 | 0.001855 | 0.012276 | -1.84513 |
| 172 | 1810043H04Rik | 1.074312 | 8.293341 | 8.482798 | 3.80E-07 | 2.27E-05 | 6.773826 |
| 173 | Cdh26 | 1.073041 | 9.362756 | 16.1676 | 5.59E-11 | 3.52E-08 | 15.51655 |
| 174 | Pole4 | 1.072856 | 7.975619 | 5.336049 | 7.99E-05 | 0.001138 | 1.32636 |
| 175 | Hs6st2 | 1.072392 | 9.597007 | 4.077813 | 0.000968 | 0.007532 | -1.19472 |
| 176 | Rasgef1c | 1.072356 | 9.301668 | 3.010381 | 0.008691 | 0.041012 | -3.36671 |
| 177 | Tgif2lx1 | 1.072055 | 6.032881 | 7.192623 | 2.90E-06 | 0.000102 | 4.704229 |
| 178 | Slc28a1 | 1.07046 | 7.960936 | 7.238989 | 2.69E-06 | 9.68E-05 | 4.782506 |
| 179 | D7Ertd595e | 1.067988 | 6.799575 | 8.19061 | 5.92E-07 | 3.16E-05 | 6.324445 |
| 180 | Nlrc3 | 1.065264 | 8.040661 | 12.7993 | 1.54E-09 | 3.99E-07 | 12.30085 |
| 181 | LOC100503168 | 1.063037 | 12.41698 | 17.00964 | 2.69E-11 | 2.47E-08 | 16.21005 |
| 182 | Arl4c | 1.060759 | 11.3608 | 12.90116 | 1.38E-09 | 3.70E-07 | 12.41 |
| 183 | Gm5106 | 1.057276 | 6.783042 | 6.101693 | 1.92E-05 | 0.000409 | 2.776155 |
| 184 | Oaf | 1.056627 | 8.318176 | 8.613713 | 3.13E-07 | 1.95E-05 | 6.971634 |
| 185 | Dgkk | 1.055663 | 5.563424 | 2.839135 | 0.012323 | 0.053611 | -3.70472 |
| 186 | Mup19 | 1.054831 | 5.236749 | 5.283401 | 8.83E-05 | 0.001232 | 1.223951 |
| 187 | Sema3f | 1.05441 | 9.319209 | 2.675005 | 0.017169 | 0.069762 | -4.02299 |
| 188 | Sema3a | 1.054404 | 5.617423 | 5.274317 | 8.99E-05 | 0.001249 | 1.206249 |
| 189 | 1700025F22Rik | 1.051779 | 7.184669 | 2.634464 | 0.018624 | 0.074291 | -4.10061 |
| 190 | LOC100046950 | 1.050837 | 5.866867 | 5.284148 | 8.82E-05 | 0.001232 | 1.225406 |
| 191 | Tas2r113 | 1.049048 | 7.712887 | 3.993636 | 0.001149 | 0.00858 | -1.36711 |
| 192 | Dmrt2 | 1.048632 | 5.542382 | 3.601088 | 0.002576 | 0.015933 | -2.17151 |
| 193 | BC048507 | 1.046748 | 11.06574 | 20.10688 | 2.35E-12 | 3.23E-09 | 18.46051 |
| 194 | Rs1 | 1.045375 | 8.695766 | 6.604533 | 7.88E-06 | 0.000212 | 3.68544 |
| 195 | 1810019J16Rik | 1.045283 | 5.504086 | 2.98244 | 0.009202 | 0.042869 | -3.42222 |
| 196 | 4930522L14Rik | 1.043211 | 5.500113 | 7.236419 | 2.70E-06 | 9.68E-05 | 4.778175 |
| 197 | Gm2692 | 1.041811 | 6.046907 | 8.610043 | 3.15E-07 | 1.96E-05 | 6.966117 |
| 198 | Cd3e | 1.036554 | 8.188043 | 16.84146 | 3.10E-11 | 2.53E-08 | 16.07462 |
| 199 | Ppp5c | 1.036292 | 9.348566 | 12.00435 | 3.75E-09 | 8.36E-07 | 11.41954 |
| 200 | Slc13a3 | 1.035702 | 10.38504 | 3.461449 | 0.003436 | 0.01986 | -2.45672 |
| 201 | Zar1l | 1.035513 | 5.252502 | 4.214441 | 0.000733 | 0.006087 | -0.9154 |
| 202 | Zic4 | 1.034199 | 5.676511 | 4.1416 | 0.00085 | 0.006793 | -1.06423 |
| 203 | Arl9 | 1.024114 | 6.340055 | 14.4081 | 2.90E-10 | 1.22E-07 | 13.9328 |
| 204 | 4933402P03Rik | 1.023953 | 7.394529 | 7.534293 | 1.66E-06 | 6.68E-05 | 5.274103 |
| 205 | 2010007E15Rik | 1.019436 | 5.464163 | 5.271046 | 9.04E-05 | 0.001254 | 1.199872 |
| 206 | Gm11961 | 1.017161 | 8.673462 | 7.566032 | 1.58E-06 | 6.44E-05 | 5.326229 |
| 207 | Tra2b | 1.014793 | 5.143477 | 13.28589 | 9.13E-10 | 2.72E-07 | 12.81488 |
| 208 | Klra1 | 1.013258 | 7.925313 | 3.050483 | 0.008006 | 0.03845 | -3.28683 |
| 209 | Gm3625 | 1.012365 | 6.306573 | 9.924793 | 4.95E-08 | 5.65E-06 | 8.8381 |
| 210 | Folr4 | 1.012268 | 5.143694 | 25.78567 | 5.95E-14 | 4.31E-10 | 21.64682 |
| 211 | Gm6209 | 1.01221 | 6.771323 | 10.92596 | 1.36E-08 | 2.16E-06 | 10.13422 |
| 212 | Ptgis | 1.004525 | 7.756218 | 2.194158 | 0.044187 | 0.143781 | -4.91092 |
| 213 | Ror2 | 1.003863 | 5.724367 | 4.090861 | 0.000942 | 0.007384 | -1.16802 |
| 214 | Rps24-ps2 | 1.00254 | 12.92773 | 13.68461 | 6.02E-10 | 1.95E-07 | 13.22254 |
| 215 | Rxfp1 | 1.001629 | 5.564156 | 3.448891 | 0.003526 | 0.020263 | -2.48231 |
| 216 | Nov | 1.000374 | 9.691319 | 2.166554 | 0.046578 | 0.149359 | -4.95937 |
| 217 | Syt2 | 0.999969 | 5.877397 | 2.559789 | 0.02162 | 0.083216 | -4.24241 |
| 218 | Gm10035 | 0.988825 | 7.982972 | 9.744902 | 6.30E-08 | 6.55E-06 | 8.593827 |
| 219 | Olfr1252 | 0.988513 | 5.524345 | 6.749591 | 6.14E-06 | 0.000176 | 3.941216 |
| 220 | D2Ertd750e | 0.98766 | 7.42068 | 16.23012 | 5.29E-11 | 3.43E-08 | 15.56939 |
| 221 | Tmem40 | 0.986487 | 7.488564 | 3.043287 | 0.008125 | 0.038897 | -3.30118 |
| 222 | Serpina1c | 0.981051 | 8.436346 | 2.522321 | 0.023293 | 0.088113 | -4.31297 |
| 223 | Slc22a18 | 0.975904 | 6.59801 | 2.832003 | 0.012503 | 0.054157 | -3.71867 |
| 224 | Meis2 | 0.975681 | 10.23683 | 2.751834 | 0.014707 | 0.061561 | -3.87478 |
| 225 | Gm6985 | 0.975664 | 5.127567 | 8.24878 | 5.41E-07 | 2.95E-05 | 6.414788 |
| 226 | Neu2 | 0.97529 | 7.089685 | 5.102167 | 0.000125 | 0.001595 | 0.868967 |
| 227 | Slc6a13 | 0.974557 | 9.870039 | 2.212389 | 0.04267 | 0.140005 | -4.87876 |
| 228 | Mapk13 | 0.968386 | 6.287137 | 3.44553 | 0.003551 | 0.020388 | -2.48916 |
| 229 | Sytl2 | 0.965381 | 8.858669 | 4.014707 | 0.001101 | 0.008288 | -1.32394 |
| 230 | Gm4978 | 0.96481 | 10.97039 | 11.75931 | 4.99E-09 | 1.03E-06 | 11.13691 |
| 231 | Plch1 | 0.964806 | 7.864918 | 3.724113 | 0.001999 | 0.013007 | -1.9196 |
| 232 | Manea | 0.964662 | 7.419063 | 9.686793 | 6.82E-08 | 6.90E-06 | 8.514148 |
| 233 | Cnr2 | 0.958998 | 8.400005 | 5.11275 | 0.000123 | 0.001572 | 0.889799 |
| 234 | Sstr5 | 0.958978 | 11.80685 | 11.52912 | 6.54E-09 | 1.24E-06 | 10.86647 |
| 235 | Gm13430 | 0.95853 | 10.07431 | 9.622046 | 7.45E-08 | 7.35E-06 | 8.424916 |
| 236 | Car7 | 0.958492 | 9.236703 | 10.52487 | 2.26E-08 | 3.11E-06 | 9.627496 |
| 237 | Slc22a2 | 0.95849 | 5.229413 | 2.995104 | 0.008967 | 0.042036 | -3.39708 |
| 238 | Rnf152 | 0.954495 | 6.808883 | 2.308863 | 0.035423 | 0.121395 | -4.70633 |
| 239 | Olfr1347 | 0.954188 | 5.236974 | 7.674009 | 1.33E-06 | 5.66E-05 | 5.502538 |
| 240 | Tmem215 | 0.953537 | 11.28987 | 7.633803 | 1.41E-06 | 5.95E-05 | 5.437073 |
| 241 | Fap | 0.949482 | 6.876983 | 3.187962 | 0.006037 | 0.030863 | -3.01121 |
| 242 | Rbp3 | 0.949285 | 10.79974 | 9.453898 | 9.39E-08 | 8.66E-06 | 8.190942 |
| 243 | Tcap | 0.948449 | 8.503391 | 3.047875 | 0.008049 | 0.038605 | -3.29203 |
| 244 | Plcxd1 | 0.948436 | 8.307824 | 6.565364 | 8.44E-06 | 0.000222 | 3.615871 |
| 245 | Gm16381 | 0.946774 | 11.28412 | 14.57463 | 2.47E-10 | 1.09E-07 | 14.09115 |
| 246 | Vmn1r48 | 0.945791 | 5.259303 | 6.286265 | 1.38E-05 | 0.00032 | 3.113979 |
| 247 | Nupr1 | 0.943988 | 9.316084 | 2.594734 | 0.020165 | 0.07882 | -4.17625 |
| 248 | Lmnb2 | 0.943074 | 7.865499 | 13.09802 | 1.12E-09 | 3.07E-07 | 12.61861 |
| 249 | Samd5 | 0.942323 | 9.242323 | 4.306306 | 0.000608 | 0.005246 | -0.72804 |
| 250 | Crxos1 | 0.93718 | 5.296262 | 9.126306 | 1.49E-07 | 1.16E-05 | 7.725645 |
| 251 | Gabrr1 | 0.930325 | 5.766669 | 6.62211 | 7.65E-06 | 0.000208 | 3.716589 |
| 252 | Gm13194 | 0.925127 | 10.75465 | 5.766062 | 3.55E-05 | 0.000637 | 2.150008 |
| 253 | H2-Q1 | 0.921249 | 5.10529 | 2.760871 | 0.01444 | 0.060805 | -3.85725 |
| 254 | Gm13178 | 0.920579 | 5.122362 | 8.521904 | 3.59E-07 | 2.15E-05 | 6.833142 |
| 255 | Tsks | 0.916354 | 6.86309 | 3.839562 | 0.001576 | 0.01086 | -1.68294 |
| 256 | Serpina1a | 0.915828 | 7.075657 | 2.483651 | 0.025148 | 0.09343 | -4.38534 |
| 257 | Gm8479 | 0.915625 | 8.597553 | 4.85024 | 0.000205 | 0.002328 | 0.369588 |
| 258 | Pink1 | 0.914939 | 12.53598 | 19.26682 | 4.38E-12 | 4.83E-09 | 17.89259 |
| 259 | Tmem54 | 0.913453 | 5.610222 | 8.346808 | 4.67E-07 | 2.63E-05 | 6.566044 |
| 260 | Obp1a | 0.911781 | 5.388864 | 6.553373 | 8.62E-06 | 0.000226 | 3.594532 |
| 261 | Trim59 | 0.91062 | 7.614193 | 7.289008 | 2.48E-06 | 9.07E-05 | 4.866617 |
| 262 | Sept1 | 0.90863 | 5.663366 | 5.697054 | 4.04E-05 | 0.000699 | 2.019414 |
| 263 | Gm7166 | 0.907799 | 5.098845 | 5.153547 | 0.000113 | 0.001479 | 0.969984 |
| 264 | Tsga8 | 0.906236 | 6.754478 | 7.38154 | 2.13E-06 | 8.17E-05 | 5.021308 |
| 265 | 4932422M17Rik | 0.905921 | 8.921735 | 13.38389 | 8.23E-10 | 2.52E-07 | 12.91619 |
| 266 | Midn | 0.905691 | 11.16613 | 7.575472 | 1.55E-06 | 6.37E-05 | 5.341707 |
| 267 | AF067063 | 0.905672 | 7.420679 | 5.558072 | 5.24E-05 | 0.000838 | 1.754533 |
| 268 | Dkkl1 | 0.904791 | 9.568171 | 3.231599 | 0.005519 | 0.028702 | -2.92322 |
| 269 | Gm5341 | 0.90404 | 10.16139 | 11.63144 | 5.80E-09 | 1.14E-06 | 10.98727 |
| 270 | Nrtn | 0.901973 | 10.25861 | 5.573871 | 5.09E-05 | 0.00082 | 1.784769 |
| 271 | Enox1 | 0.900297 | 5.176783 | 8.937637 | 1.95E-07 | 1.39E-05 | 7.451861 |
| 272 | Ylpm1 | 0.899884 | 6.474987 | 8.709952 | 2.72E-07 | 1.77E-05 | 7.115671 |
| 273 | Col1a2 | 0.899688 | 11.06638 | 2.381253 | 0.030761 | 0.10882 | -4.57467 |
| 274 | Sart1 | 0.899328 | 10.74073 | 10.05925 | 4.14E-08 | 5.00E-06 | 9.018344 |
| 275 | Mup2 | 0.896979 | 5.117688 | 6.130103 | 1.83E-05 | 0.000393 | 2.828457 |
| 276 | Ptgfrn | 0.89504 | 6.012267 | 4.611052 | 0.000329 | 0.0033 | -0.11022 |
| 277 | Plxnd1 | 0.894665 | 9.45951 | 2.865707 | 0.011675 | 0.051442 | -3.65263 |
| 278 | Rpl7a | 0.894414 | 13.23573 | 16.46827 | 4.28E-11 | 3.05E-08 | 15.76865 |
| 279 | Trpv6 | 0.89306 | 7.576922 | 4.92627 | 0.000177 | 0.002084 | 0.520986 |
| 280 | Gm6570 | 0.892461 | 11.67933 | 20.9526 | 1.28E-12 | 2.17E-09 | 19.0038 |
| 281 | Cux2 | 0.889342 | 8.143894 | 3.980951 | 0.001179 | 0.008734 | -1.3931 |
| 282 | Vrk2 | 0.889109 | 7.512087 | 2.743996 | 0.014941 | 0.062366 | -3.88997 |
| 283 | Bgn | 0.88818 | 5.768553 | 4.32118 | 0.00059 | 0.005136 | -0.69775 |
| 284 | Map6d1 | 0.886918 | 11.01409 | 11.89661 | 4.25E-09 | 9.10E-07 | 11.29594 |
| 285 | Pxt1 | 0.882596 | 5.132625 | 6.53543 | 8.89E-06 | 0.000231 | 3.562561 |
| 286 | Gm6537 | 0.881684 | 5.180244 | 3.410958 | 0.003813 | 0.021538 | -2.55956 |
| 287 | COX2 | 0.881488 | 17.55276 | 9.318811 | 1.13E-07 | 9.92E-06 | 8.000601 |
| 288 | Atp10b | 0.880243 | 5.860601 | 2.338271 | 0.033454 | 0.11622 | -4.65307 |
| 289 | Tshz3 | 0.879587 | 10.79193 | 3.48986 | 0.00324 | 0.019017 | -2.39877 |
| 290 | Pcp2 | 0.87916 | 7.402679 | 4.011984 | 0.001107 | 0.008329 | -1.32952 |
| 291 | Cited4 | 0.878933 | 8.061146 | 2.39234 | 0.030101 | 0.107032 | -4.55434 |
| 292 | Rai14 | 0.878014 | 8.759619 | 3.456491 | 0.003471 | 0.020016 | -2.46682 |
| 293 | Gm11223 | 0.876943 | 13.72071 | 4.776029 | 0.000237 | 0.002583 | 0.221275 |
| 294 | Rlbp1 | 0.876145 | 7.543937 | 6.024222 | 2.21E-05 | 0.000455 | 2.632971 |
| 295 | Lce1a2 | 0.875926 | 8.103447 | 8.276078 | 5.19E-07 | 2.86E-05 | 6.457033 |
| 296 | Lhx3 | 0.875342 | 5.670712 | 2.234289 | 0.040913 | 0.135653 | -4.83994 |
| 297 | Mei1 | 0.874521 | 6.410038 | 3.538099 | 0.002933 | 0.017634 | -2.30028 |
| 298 | 2810007J24Rik | 0.872696 | 6.262162 | 8.557832 | 3.40E-07 | 2.08E-05 | 6.887465 |
| 299 | Cyp2t4 | 0.867998 | 5.354613 | 3.257657 | 0.00523 | 0.027576 | -2.87058 |
| 300 | Cym | 0.867457 | 6.370076 | 11.00669 | 1.23E-08 | 2.00E-06 | 10.23426 |
| 301 | Fam5c | 0.862204 | 8.717669 | 3.762616 | 0.001847 | 0.012237 | -1.84069 |
| 302 | Ifit1 | 0.861515 | 5.737366 | 3.931744 | 0.001305 | 0.009455 | -1.49395 |
| 303 | Efna5 | 0.860612 | 9.461577 | 3.030666 | 0.008338 | 0.03963 | -3.32634 |
| 304 | Gadd45a | 0.858938 | 7.772333 | 4.686056 | 0.000284 | 0.002962 | 0.040783 |
| 305 | Stam2 | 0.856067 | 6.969225 | 9.440277 | 9.57E-08 | 8.79E-06 | 8.171846 |
| 306 | Tcfcp2l1 | 0.855449 | 5.853062 | 4.38766 | 0.000516 | 0.004608 | -0.56251 |
| 307 | Tmsb10 | 0.852352 | 14.30199 | 3.506756 | 0.003129 | 0.018514 | -2.36429 |
| 308 | ND3 | 0.851854 | 15.7323 | 9.278569 | 1.20E-07 | 1.02E-05 | 7.943486 |
| 309 | Krtap8-2 | 0.849873 | 6.430805 | 6.707354 | 6.60E-06 | 0.000185 | 3.867045 |
| 310 | Prg4 | 0.849342 | 5.056189 | 3.810372 | 0.001674 | 0.011379 | -1.74279 |
| 311 | Taf13 | 0.848381 | 9.20973 | 6.132173 | 1.82E-05 | 0.000393 | 2.832265 |
| 312 | 4933440N22Rik | 0.846895 | 6.028567 | 7.574159 | 1.56E-06 | 6.37E-05 | 5.339554 |
| 313 | Fhod3 | 0.8457 | 9.805211 | 3.221848 | 0.00563 | 0.029177 | -2.9429 |
| 314 | LOC100046692 | 0.844516 | 10.39731 | 9.915685 | 5.01E-08 | 5.69E-06 | 8.825819 |
| 315 | Olfr319 | 0.842329 | 5.951682 | 8.988746 | 1.81E-07 | 1.33E-05 | 7.526452 |
| 316 | Limk1 | 0.840971 | 11.81062 | 10.05622 | 4.15E-08 | 5.00E-06 | 9.014299 |
| 317 | Fam18b | 0.839023 | 9.266722 | 10.95688 | 1.31E-08 | 2.09E-06 | 10.17261 |
| 318 | Slc4a10 | 0.83896 | 8.896405 | 7.461868 | 1.87E-06 | 7.36E-05 | 5.154644 |
| 319 | Gm6323 | 0.838543 | 9.378101 | 9.243945 | 1.26E-07 | 1.05E-05 | 7.894191 |
| 320 | Fut9 | 0.836396 | 8.393948 | 6.425733 | 1.08E-05 | 0.000267 | 3.366131 |
| 321 | Cckbr | 0.836112 | 7.496424 | 3.375234 | 0.004105 | 0.022752 | -2.63222 |
| 322 | Ovch2 | 0.835263 | 5.658766 | 6.356869 | 1.22E-05 | 0.000295 | 3.241964 |
| 323 | Prph | 0.835055 | 7.652858 | 3.36807 | 0.004166 | 0.023032 | -2.64678 |
| 324 | Adra2b | 0.835007 | 5.350423 | 3.156236 | 0.006444 | 0.032521 | -3.07503 |
| 325 | Klf15 | 0.834403 | 8.218684 | 5.363625 | 7.58E-05 | 0.001092 | 1.379868 |
| 326 | Klf5 | 0.832176 | 8.441449 | 2.973695 | 0.009368 | 0.043449 | -3.43957 |
| 327 | Olfr549 | 0.831666 | 5.760723 | 4.390818 | 0.000513 | 0.004586 | -0.55609 |
| 328 | Arhgef16 | 0.830864 | 5.226413 | 3.801891 | 0.001703 | 0.011516 | -1.76017 |
| 329 | Unc13c | 0.830234 | 9.65242 | 3.39414 | 0.003948 | 0.022126 | -2.59378 |
| 330 | 1700084J12Rik | 0.82985 | 5.065217 | 7.428569 | 1.97E-06 | 7.70E-05 | 5.099478 |
| 331 | Als2cr4 | 0.829084 | 7.083355 | 10.65026 | 1.92E-08 | 2.80E-06 | 9.78767 |
| 332 | Dcaf13 | 0.827221 | 8.195068 | 4.141853 | 0.000849 | 0.006792 | -1.06371 |
| 333 | Atp5o | 0.825717 | 13.33406 | 9.458169 | 9.34E-08 | 8.65E-06 | 8.196925 |
| 334 | Uqcrq | 0.825527 | 14.46063 | 25.45844 | 7.20E-14 | 4.31E-10 | 21.48947 |
| 335 | Abhd12b | 0.825146 | 5.098563 | 7.432584 | 1.96E-06 | 7.66E-05 | 5.106138 |
| 336 | Coch | 0.824699 | 6.157141 | 2.636373 | 0.018553 | 0.074123 | -4.09696 |
| 337 | Gpr97 | 0.822279 | 5.054509 | 3.911615 | 0.00136 | 0.009754 | -1.53521 |
| 338 | Kcnq1 | 0.821046 | 5.439655 | 3.859159 | 0.001514 | 0.010514 | -1.64276 |
| 339 | Trappc2 | 0.820027 | 7.002066 | 7.94931 | 8.59E-07 | 4.12E-05 | 5.944951 |
| 340 | BC080695 | 0.819396 | 5.797065 | 5.819689 | 3.22E-05 | 0.000592 | 2.251062 |
| 341 | Cox7c | 0.8188 | 10.83815 | 17.93171 | 1.25E-11 | 1.20E-08 | 16.92676 |
| 342 | Ano4 | 0.818703 | 7.654802 | 4.622868 | 0.000322 | 0.003249 | -0.0864 |
| 343 | Rpl17 | 0.817665 | 13.45656 | 22.63365 | 4.11E-13 | 1.01E-09 | 20.0064 |
| 344 | Srpx2 | 0.817176 | 7.310019 | 5.327068 | 8.12E-05 | 0.001154 | 1.308914 |
| 345 | Plcd3 | 0.817096 | 9.824229 | 5.850829 | 3.04E-05 | 0.000568 | 2.309568 |
| 346 | Plscr2 | 0.815195 | 5.716828 | 4.432209 | 0.000472 | 0.004308 | -0.47203 |
| 347 | Gm3696 | 0.812812 | 5.922582 | 4.192649 | 0.000766 | 0.006292 | -0.9599 |
| 348 | Penk | 0.808944 | 11.76128 | 2.41102 | 0.029018 | 0.10419 | -4.51999 |
| 349 | Fam180a | 0.807664 | 5.06129 | 2.521561 | 0.023328 | 0.088216 | -4.31439 |
| 350 | Chrna4 | 0.806143 | 5.839018 | 2.636849 | 0.018535 | 0.074084 | -4.09605 |
| 351 | LOC270362 | 0.80576 | 13.63694 | 8.945596 | 1.93E-07 | 1.38E-05 | 7.463498 |
| 352 | Garnl3 | 0.805165 | 11.61883 | 3.198743 | 0.005905 | 0.030362 | -2.98949 |
| 353 | Sox13 | 0.803769 | 7.707904 | 7.016919 | 3.89E-06 | 0.000128 | 4.404892 |
| 354 | Arid4b | 0.802297 | 6.647405 | 4.471407 | 0.000436 | 0.00407 | -0.39253 |
| 355 | Prrx2 | 0.801267 | 7.269516 | 2.786208 | 0.013719 | 0.058381 | -3.80802 |
| 356 | S100a11 | 0.801006 | 7.648891 | 3.530244 | 0.002981 | 0.017844 | -2.31633 |
| 357 | Scai | 0.800451 | 6.286442 | 2.315968 | 0.034938 | 0.120049 | -4.6935 |
| 358 | Hmg1l1 | 0.800029 | 12.22463 | 13.79547 | 5.37E-10 | 1.87E-07 | 13.33378 |
| 359 | Gm749 | 0.799744 | 5.507248 | 4.422419 | 0.000481 | 0.004376 | -0.4919 |
| 360 | Ier5l | 0.797162 | 12.07644 | 5.469077 | 6.20E-05 | 0.000945 | 1.583636 |
| 361 | Oas1f | 0.796405 | 5.550071 | 4.728697 | 0.000261 | 0.002784 | 0.126414 |
| 362 | Clec2d | 0.795848 | 6.351465 | 3.7094 | 0.002061 | 0.013326 | -1.94975 |
| 363 | A430088P11Rik | 0.795681 | 6.059142 | 4.999757 | 0.000153 | 0.001866 | 0.666765 |
| 364 | Rpl21-ps1 | 0.794749 | 12.17823 | 25.31705 | 7.82E-14 | 4.31E-10 | 21.42061 |
| 365 | Gucy1a3 | 0.794388 | 10.46876 | 4.289992 | 0.000628 | 0.005372 | -0.76129 |
| 366 | Svep1 | 0.791089 | 5.646174 | 2.523279 | 0.023248 | 0.088011 | -4.31117 |
| 367 | Slk | 0.790317 | 6.031296 | 8.8365 | 2.26E-07 | 1.55E-05 | 7.303315 |
| 368 | Mrap | 0.78783 | 5.804146 | 2.502783 | 0.024213 | 0.090799 | -4.34959 |
| 369 | Gpr182 | 0.785831 | 7.958626 | 2.398174 | 0.029758 | 0.106175 | -4.54362 |
| 370 | Pkhd1 | 0.784502 | 5.400277 | 4.5157 | 0.000399 | 0.003807 | -0.30283 |
| 371 | Cfhr2 | 0.784366 | 8.18046 | 4.063488 | 0.000996 | 0.007699 | -1.22404 |
| 372 | Dck | 0.783299 | 6.854615 | 7.695073 | 1.28E-06 | 5.52E-05 | 5.536748 |
| 373 | 4930519P11Rik | 0.783095 | 5.136598 | 6.64972 | 7.29E-06 | 0.0002 | 3.765431 |
| 374 | Gm5878 | 0.781615 | 7.439103 | 5.959262 | 2.49E-05 | 0.000494 | 2.512285 |
| 375 | 1600014K23Rik | 0.780954 | 5.133854 | 2.454871 | 0.026618 | 0.09748 | -4.4389 |
| 376 | Ep400 | 0.776112 | 9.935356 | 8.916954 | 2.01E-07 | 1.42E-05 | 7.421585 |
| 377 | Mthfd2 | 0.774426 | 6.654572 | 6.38858 | 1.15E-05 | 0.000282 | 3.299223 |
| 378 | Hspa12a | 0.774038 | 8.598297 | 4.197352 | 0.000758 | 0.006246 | -0.95029 |
| 379 | Dnalc1 | 0.773855 | 5.250602 | 4.179522 | 0.000786 | 0.006407 | -0.98672 |
| 380 | Gm129 | 0.771892 | 6.372355 | 2.894962 | 0.011001 | 0.049028 | -3.59513 |
| 381 | Nlgn1 | 0.771465 | 9.99091 | 9.458506 | 9.33E-08 | 8.65E-06 | 8.197397 |
| 382 | Zglp1 | 0.771426 | 9.334627 | 11.13734 | 1.05E-08 | 1.77E-06 | 10.39482 |
| 383 | Col5a1 | 0.771281 | 6.816439 | 3.705376 | 0.002078 | 0.013408 | -1.95799 |
| 384 | Padi1 | 0.770613 | 6.445234 | 11.94581 | 4.02E-09 | 8.68E-07 | 11.35252 |
| 385 | Ly6g6e | 0.765502 | 9.418776 | 2.136162 | 0.049348 | 0.15624 | -5.01233 |
| 386 | Krt16 | 0.765502 | 6.551881 | 6.910463 | 4.66E-06 | 0.000145 | 4.22144 |
| 387 | Cish | 0.765294 | 8.391039 | 4.422927 | 0.00048 | 0.004373 | -0.49087 |
| 388 | Myh4 | 0.763733 | 5.670398 | 2.227535 | 0.041448 | 0.137049 | -4.85193 |
| 389 | Pip5kl1 | 0.763471 | 9.168865 | 4.385386 | 0.000518 | 0.004625 | -0.56713 |
| 390 | Rrm2b | 0.763465 | 5.125613 | 10.13811 | 3.73E-08 | 4.62E-06 | 9.123136 |
| 391 | Prp2 | 0.763377 | 7.011299 | 5.633162 | 4.55E-05 | 0.000761 | 1.897953 |
| 392 | Gcgr | 0.762816 | 7.969328 | 6.681802 | 6.90E-06 | 0.000191 | 3.822051 |
| 393 | Gm2138 | 0.760873 | 7.248346 | 5.624751 | 4.63E-05 | 0.000768 | 1.881923 |
| 394 | Gabra1 | 0.760257 | 12.42018 | 6.131315 | 1.82E-05 | 0.000393 | 2.830686 |
| 395 | Slc25a22 | 0.760162 | 10.72188 | 6.516821 | 9.19E-06 | 0.000237 | 3.529357 |
| 396 | Cdh1 | 0.758939 | 5.051834 | 2.547571 | 0.022153 | 0.0849 | -4.26547 |
| 397 | Prf1 | 0.758401 | 5.547349 | 3.04305 | 0.008129 | 0.038904 | -3.30166 |
| 398 | Ptk7 | 0.757092 | 8.925463 | 6.73523 | 6.29E-06 | 0.000179 | 3.916025 |
| 399 | Gm590 | 0.754603 | 5.248498 | 3.854319 | 0.001529 | 0.010592 | -1.65268 |
| 400 | Ints3 | 0.752981 | 9.2803 | 8.829099 | 2.28E-07 | 1.56E-05 | 7.292396 |
| 401 | Prpmp5 | 0.752246 | 6.926611 | 14.01409 | 4.30E-10 | 1.63E-07 | 13.55058 |
| 402 | Dlx3 | 0.752071 | 10.71022 | 4.696929 | 0.000278 | 0.002913 | 0.062633 |
| 403 | Hes2 | 0.750963 | 5.118825 | 6.493387 | 9.57E-06 | 0.000245 | 3.487475 |
| 404 | Gm7349 | 0.750393 | 5.310896 | 5.139022 | 0.000117 | 0.00151 | 0.941457 |
| 405 | Gm4907 | 0.74886 | 5.420085 | 2.90315 | 0.010819 | 0.04841 | -3.579 |
| 406 | Prr9 | 0.748846 | 5.014675 | 9.62268 | 7.45E-08 | 7.35E-06 | 8.425791 |
| 407 | Plekha4 | 0.747894 | 7.216831 | 3.305477 | 0.004739 | 0.025476 | -2.77379 |
| 408 | Ovgp1 | 0.747894 | 9.610085 | 3.654217 | 0.002309 | 0.014629 | -2.06278 |
| 409 | Ankrd34c | 0.747399 | 5.280982 | 2.69579 | 0.016466 | 0.067428 | -3.98304 |
| 410 | Gm6320 | 0.746678 | 14.40002 | 7.140696 | 3.17E-06 | 0.000108 | 4.61621 |
| 411 | Fn1 | 0.74329 | 11.61581 | 3.039054 | 0.008196 | 0.03913 | -3.30962 |
| 412 | Adam33 | 0.742017 | 5.898954 | 4.77336 | 0.000239 | 0.002591 | 0.215932 |
| 413 | Rasgef1b | 0.741591 | 10.38886 | 2.541383 | 0.022427 | 0.085677 | -4.27712 |
| 414 | LOC100502641 | 0.741521 | 16.18403 | 8.621248 | 3.09E-07 | 1.95E-05 | 6.982952 |
| 415 | Pcgf5 | 0.738307 | 8.00625 | 6.03821 | 2.16E-05 | 0.000446 | 2.658885 |
| 416 | Dusp26 | 0.737182 | 13.20463 | 3.528302 | 0.002993 | 0.017891 | -2.3203 |
| 417 | Prkg2 | 0.73503 | 8.296419 | 2.266199 | 0.038473 | 0.129241 | -4.78303 |
| 418 | Gm12471 | 0.733941 | 10.74654 | 8.789032 | 2.42E-07 | 1.65E-05 | 7.233163 |
| 419 | 1700081L11Rik | 0.731193 | 6.791087 | 6.090045 | 1.96E-05 | 0.000417 | 2.75468 |
| 420 | Olfr671 | 0.730478 | 5.029772 | 4.137687 | 0.000856 | 0.006843 | -1.07223 |
| 421 | Sts | 0.730471 | 10.58643 | 6.264946 | 1.43E-05 | 0.00033 | 3.075198 |
| 422 | Gm16442 | 0.73029 | 10.17149 | 2.632807 | 0.018686 | 0.074471 | -4.10377 |
| 423 | Pamr1 | 0.729307 | 9.969686 | 3.034244 | 0.008277 | 0.039415 | -3.31921 |
| 424 | Dusp10 | 0.72891 | 5.596836 | 4.146251 | 0.000842 | 0.006751 | -1.05472 |
| 425 | Pycard | 0.728837 | 8.046488 | 9.177131 | 1.38E-07 | 1.12E-05 | 7.798666 |
| 426 | ND4 | 0.728595 | 16.54951 | 15.64482 | 8.96E-11 | 5.31E-08 | 15.06587 |
| 427 | Igfbp4 | 0.727752 | 7.798297 | 5.330436 | 8.07E-05 | 0.001148 | 1.315459 |
| 428 | Nr5a1 | 0.727603 | 5.999291 | 7.234215 | 2.71E-06 | 9.70E-05 | 4.77446 |
| 429 | Kcnt1 | 0.727471 | 9.739312 | 3.109557 | 0.007092 | 0.035008 | -3.16871 |
| 430 | Galnt7 | 0.727033 | 10.32219 | 6.333884 | 1.27E-05 | 0.000303 | 3.200375 |
| 431 | Gm4910 | 0.727031 | 12.64 | 7.009786 | 3.94E-06 | 0.000129 | 4.392649 |
| 432 | Def6 | 0.726612 | 10.3084 | 9.8143 | 5.74E-08 | 6.26E-06 | 8.68849 |
| 433 | Krtap16-4 | 0.725937 | 11.26073 | 7.934113 | 8.80E-07 | 4.20E-05 | 5.920793 |
| 434 | Nr1i3 | 0.725766 | 5.08509 | 6.555923 | 8.58E-06 | 0.000225 | 3.59907 |
| 435 | Wfikkn2 | 0.724191 | 5.273419 | 3.160429 | 0.006389 | 0.032323 | -3.06661 |
| 436 | Zfp455 | 0.723358 | 5.067022 | 4.616481 | 0.000326 | 0.003279 | -0.09927 |
| 437 | Tmem232 | 0.722797 | 6.129444 | 2.835745 | 0.012408 | 0.053831 | -3.71135 |
| 438 | Kif5b | 0.72248 | 5.082888 | 5.909917 | 2.73E-05 | 0.000527 | 2.420228 |
| 439 | Mepe | 0.721586 | 5.073508 | 5.918737 | 2.68E-05 | 0.000522 | 2.436706 |
| 440 | Pex14 | 0.720785 | 8.891287 | 13.14396 | 1.06E-09 | 3.00E-07 | 12.66686 |
| 441 | 1700001K19Rik | 0.72036 | 8.682319 | 8.624481 | 3.08E-07 | 1.95E-05 | 6.987807 |
| 442 | Slc18a3 | 0.719804 | 5.118754 | 5.891859 | 2.82E-05 | 0.00054 | 2.386458 |
| 443 | Gm3993 | 0.717029 | 9.023616 | 9.529745 | 8.46E-08 | 8.14E-06 | 8.296884 |
| 444 | 1500004F05Rik | 0.716967 | 7.955857 | 4.452813 | 0.000452 | 0.004175 | -0.43023 |
| 445 | Cabp5 | 0.716616 | 8.22161 | 3.964709 | 0.001219 | 0.008976 | -1.42638 |
| 446 | Cnga2 | 0.716269 | 7.021814 | 8.125716 | 6.54E-07 | 3.41E-05 | 6.223138 |
| 447 | E130116L18Rik | 0.715407 | 6.713246 | 5.435237 | 6.61E-05 | 0.000989 | 1.518394 |
| 448 | Akap8 | 0.715339 | 9.028653 | 7.184168 | 2.94E-06 | 0.000103 | 4.689923 |
| 449 | Uts2r | 0.714986 | 7.281122 | 7.863403 | 9.83E-07 | 4.61E-05 | 5.807986 |
| 450 | Foxc1 | 0.712256 | 8.1046 | 2.663215 | 0.017581 | 0.07108 | -4.04561 |
| 451 | Necab3 | 0.711615 | 11.94482 | 5.769488 | 3.53E-05 | 0.000635 | 2.156475 |
| 452 | Gm3896 | 0.711148 | 5.248485 | 5.374683 | 7.42E-05 | 0.001075 | 1.4013 |
| 453 | Alpl | 0.711051 | 7.091791 | 4.295924 | 0.000621 | 0.005331 | -0.7492 |
| 454 | Gm3988 | 0.711021 | 13.32538 | 12.21621 | 2.95E-09 | 6.76E-07 | 11.65966 |
| 455 | Epha8 | 0.710827 | 9.083189 | 3.888108 | 0.001427 | 0.01011 | -1.5834 |
| 456 | Amn | 0.710285 | 9.292208 | 2.770706 | 0.014156 | 0.059848 | -3.83816 |
| 457 | Mybpc1 | 0.710253 | 6.152302 | 2.501298 | 0.024284 | 0.09099 | -4.35237 |
| 458 | Utp20 | 0.710103 | 7.391935 | 9.200176 | 1.34E-07 | 1.10E-05 | 7.831674 |
| 459 | Adcy8 | 0.709439 | 8.009733 | 3.651372 | 0.002322 | 0.014681 | -2.0686 |
| 460 | 4933427D06Rik | 0.708424 | 11.15157 | 4.503362 | 0.000409 | 0.003871 | -0.3278 |
| 461 | Fam70b | 0.707499 | 7.478585 | 4.751033 | 0.000249 | 0.002685 | 0.171205 |
| 462 | Alox12e | 0.707486 | 4.97061 | 2.4013 | 0.029576 | 0.105594 | -4.53787 |
| 463 | Gm11937 | 0.707467 | 7.426863 | 8.550281 | 3.44E-07 | 2.09E-05 | 6.876061 |
| 464 | Gm10318 | 0.706979 | 8.705894 | 9.561905 | 8.09E-08 | 7.86E-06 | 8.341604 |
| 465 | Pitpnm3 | 0.706811 | 10.02671 | 4.085443 | 0.000953 | 0.00745 | -1.1791 |
| 466 | Oas2 | 0.70625 | 5.226312 | 3.597603 | 0.002594 | 0.016017 | -2.17864 |
| 467 | Fxyd5 | 0.704767 | 9.143324 | 3.073068 | 0.007644 | 0.037106 | -3.24173 |
| 468 | Thbs1 | 0.704497 | 5.241031 | 2.199085 | 0.043772 | 0.142727 | -4.90224 |
| 469 | Cd22 | 0.70383 | 6.386778 | 11.44451 | 7.24E-09 | 1.33E-06 | 10.76583 |
| 470 | Gm6718 | 0.703769 | 13.56136 | 15.62049 | 9.16E-11 | 5.31E-08 | 15.0445 |
| 471 | Myo3b | 0.703484 | 6.882125 | 4.581442 | 0.00035 | 0.003451 | -0.16995 |
| 472 | Nbn | 0.70243 | 7.813816 | 7.049613 | 3.69E-06 | 0.000123 | 4.460915 |
| 473 | Med23 | 0.702425 | 7.766815 | 10.42497 | 2.57E-08 | 3.51E-06 | 9.498735 |
| 474 | Serpina1e | 0.701035 | 7.03255 | 2.250822 | 0.039632 | 0.13246 | -4.8105 |
| 475 | Ddhd1 | 0.700789 | 8.332831 | 10.31681 | 2.95E-08 | 3.87E-06 | 9.35814 |
| 476 | Sdad1 | 0.700641 | 8.336855 | 5.090273 | 0.000128 | 0.00162 | 0.845543 |
| 477 | Dpp10 | 0.699211 | 10.54225 | 2.618782 | 0.019218 | 0.076015 | -4.13052 |
| 478 | 4930401B11Rik | 0.699108 | 14.14611 | 12.46019 | 2.24E-09 | 5.36E-07 | 11.9314 |
| 479 | Olfr522 | 0.699054 | 6.405934 | 4.704924 | 0.000273 | 0.002878 | 0.078692 |
| 480 | Lmbr1 | 0.698531 | 6.228538 | 4.92762 | 0.000176 | 0.00208 | 0.52367 |
| 481 | Gm6484 | 0.697544 | 6.320577 | 3.942501 | 0.001276 | 0.009295 | -1.4719 |
| 482 | Serpina1d | 0.697365 | 5.453525 | 2.409221 | 0.02912 | 0.104456 | -4.5233 |
| 483 | Gabra3 | 0.696878 | 8.558121 | 2.60862 | 0.019613 | 0.077174 | -4.14986 |
| 484 | Serpina3b | 0.695451 | 5.810464 | 5.234685 | 9.70E-05 | 0.00132 | 1.128901 |
| 485 | Tspo2 | 0.695346 | 6.176697 | 5.883668 | 2.86E-05 | 0.000545 | 2.371128 |
| 486 | Cdh18 | 0.693775 | 6.658224 | 2.284852 | 0.037111 | 0.125925 | -4.74958 |
| 487 | Adamts2 | 0.692103 | 5.880984 | 3.503813 | 0.003148 | 0.018603 | -2.3703 |
| 488 | Zfp57 | 0.691228 | 6.586039 | 6.760283 | 6.02E-06 | 0.000173 | 3.959953 |
| 489 | E330017A01Rik | 0.69069 | 5.036124 | 11.42443 | 7.42E-09 | 1.35E-06 | 10.74184 |
| 490 | Kcnj12 | 0.689757 | 8.740546 | 2.620458 | 0.019154 | 0.075869 | -4.12732 |
| 491 | Vsig8 | 0.689575 | 7.541977 | 2.877918 | 0.011389 | 0.050402 | -3.62865 |
| 492 | March9 | 0.689334 | 11.92788 | 7.739516 | 1.20E-06 | 5.24E-05 | 5.60873 |
| 493 | Spon2 | 0.688112 | 4.989991 | 2.219174 | 0.042119 | 0.138657 | -4.86675 |
| 494 | Gins3 | 0.686471 | 11.89876 | 11.16539 | 1.01E-08 | 1.73E-06 | 10.42906 |
| 495 | Gm12824 | 0.686323 | 5.864347 | 3.99084 | 0.001156 | 0.008618 | -1.37284 |
| 496 | Rapgef1 | 0.686178 | 10.13211 | 6.580541 | 8.22E-06 | 0.00022 | 3.642852 |
| 497 | Tes | 0.684435 | 6.2038 | 3.036112 | 0.008245 | 0.039316 | -3.31549 |
| 498 | E130012A19Rik | 0.684069 | 12.18773 | 2.755192 | 0.014607 | 0.061312 | -3.86827 |
| 499 | Cdh20 | 0.682458 | 7.051732 | 4.133061 | 0.000864 | 0.006885 | -1.08169 |
| 500 | Oog2 | 0.680394 | 4.982009 | 11.80232 | 4.74E-09 | 9.96E-07 | 11.18691 |
| 501 | Hes7 | 0.679961 | 9.482974 | 7.878075 | 9.61E-07 | 4.52E-05 | 5.831448 |
| 502 | 9030619P08Rik | 0.679926 | 5.17991 | 3.822464 | 0.001633 | 0.011168 | -1.71799 |
| 503 | BC028777 | 0.679834 | 5.037781 | 7.950608 | 8.57E-07 | 4.12E-05 | 5.947013 |
| 504 | Fam194a | 0.679702 | 7.382877 | 8.950504 | 1.91E-07 | 1.38E-05 | 7.470669 |
| 505 | Gm13023 | 0.679537 | 7.126091 | 12.82609 | 1.50E-09 | 3.92E-07 | 12.32964 |
| 506 | H1foo | 0.678498 | 4.973527 | 6.100751 | 1.93E-05 | 0.000409 | 2.774419 |
| 507 | Tbx10 | 0.678116 | 5.816131 | 5.646944 | 4.44E-05 | 0.000745 | 1.924196 |
| 508 | Cxcl11 | 0.677809 | 13.72387 | 4.642287 | 0.00031 | 0.003165 | -0.04728 |
| 509 | Fam43a | 0.677442 | 8.284087 | 2.495962 | 0.024542 | 0.091769 | -4.36235 |
| 510 | 5430401F13Rik | 0.676992 | 5.494989 | 4.328512 | 0.000581 | 0.005078 | -0.68282 |
| 511 | Tomm22 | 0.676282 | 10.5865 | 8.054639 | 7.29E-07 | 3.66E-05 | 6.111545 |
| 512 | Gm2137 | 0.676166 | 7.661044 | 7.484101 | 1.80E-06 | 7.18E-05 | 5.191391 |
| 513 | Sdc1 | 0.676056 | 5.321591 | 2.27493 | 0.03783 | 0.127755 | -4.76739 |
| 514 | Ltbr | 0.67564 | 8.691234 | 6.894363 | 4.79E-06 | 0.000149 | 4.193558 |
| 515 | Slc6a20a | 0.675466 | 8.362577 | 2.34337 | 0.033124 | 0.115253 | -4.64381 |
| 516 | Tll1 | 0.675234 | 6.835964 | 5.494072 | 5.91E-05 | 0.000912 | 1.631733 |
| 517 | Hmgb3 | 0.67373 | 10.08761 | 4.418091 | 0.000485 | 0.004402 | -0.50069 |
| 518 | Hemt1 | 0.673001 | 5.007545 | 5.195874 | 0.000105 | 0.001396 | 1.052979 |
| 519 | Apobec3 | 0.672831 | 6.906247 | 12.76544 | 1.60E-09 | 4.05E-07 | 12.26438 |
| 520 | Slc22a1 | 0.671861 | 4.985682 | 3.505262 | 0.003139 | 0.018566 | -2.36734 |
| 521 | Pde4c | 0.670332 | 14.44808 | 5.767275 | 3.55E-05 | 0.000637 | 2.152298 |
| 522 | Olfr629 | 0.670181 | 5.933858 | 5.220093 | 9.98E-05 | 0.001348 | 1.100376 |
| 523 | Slc16a12 | 0.668687 | 5.284639 | 2.917217 | 0.010513 | 0.047423 | -3.55127 |
| 524 | Sema3b | 0.66844 | 8.167908 | 2.728637 | 0.015412 | 0.063941 | -3.91968 |
| 525 | Cthrc1 | 0.66785 | 7.008166 | 4.383978 | 0.00052 | 0.004635 | -0.56999 |
| 526 | Dars2 | 0.666604 | 7.26409 | 10.38659 | 2.70E-08 | 3.62E-06 | 9.448983 |
| 527 | Gbp5 | 0.666584 | 6.142635 | 3.328061 | 0.004524 | 0.024553 | -2.728 |
| 528 | Cdhr2 | 0.666507 | 8.739638 | 5.984216 | 2.38E-05 | 0.000477 | 2.558714 |
| 529 | AW495222 | 0.666491 | 5.750634 | 3.787609 | 0.001754 | 0.011772 | -1.78945 |
| 530 | Stat5b | 0.666026 | 9.67261 | 4.006727 | 0.001119 | 0.008396 | -1.34029 |
| 531 | Ctdsp2 | 0.66548 | 7.682904 | 10.31294 | 2.97E-08 | 3.87E-06 | 9.353094 |
| 532 | Dennd1a | 0.665378 | 9.13105 | 13.70858 | 5.87E-10 | 1.93E-07 | 13.24666 |
| 533 | Cdc42ep3 | 0.664214 | 8.244691 | 2.802702 | 0.013268 | 0.056725 | -3.77589 |
| 534 | 1810063B05Rik | 0.663629 | 10.26162 | 10.55091 | 2.18E-08 | 3.05E-06 | 9.660892 |
| 535 | Nfe2l3 | 0.663222 | 5.087249 | 3.152692 | 0.006491 | 0.032669 | -3.08216 |
| 536 | Muc20 | 0.663076 | 5.562862 | 7.684936 | 1.30E-06 | 5.58E-05 | 5.520292 |
| 537 | Lrrc3b | 0.662879 | 7.712864 | 6.565603 | 8.44E-06 | 0.000222 | 3.616297 |
| 538 | Itpa | 0.662675 | 11.07344 | 16.62189 | 3.75E-11 | 2.85E-08 | 15.89552 |
| 539 | Cdh7 | 0.662096 | 7.067561 | 2.276689 | 0.037701 | 0.127478 | -4.76424 |
| 540 | 5830403L16Rik | 0.661962 | 6.721756 | 14.923 | 1.76E-10 | 8.44E-08 | 14.41645 |
| 541 | Lpo | 0.661879 | 5.655876 | 4.338387 | 0.00057 | 0.005001 | -0.66272 |
| 542 | Snai1 | 0.661517 | 5.069076 | 2.226112 | 0.041561 | 0.137306 | -4.85445 |
| 543 | Gm6813 | 0.66137 | 14.73689 | 20.2523 | 2.11E-12 | 3.10E-09 | 18.55591 |
| 544 | Rabif | 0.66095 | 8.983567 | 4.319815 | 0.000592 | 0.005143 | -0.70053 |
| 545 | Hs3st2 | 0.660698 | 8.485163 | 2.267335 | 0.038389 | 0.12905 | -4.781 |
| 546 | Ttc39b | 0.660474 | 6.168419 | 4.630127 | 0.000317 | 0.003219 | -0.07177 |
| 547 | Ypel2 | 0.659897 | 8.694826 | 5.130114 | 0.000119 | 0.00153 | 0.923949 |
| 548 | Lrrc38 | 0.659373 | 7.251882 | 8.810073 | 2.35E-07 | 1.60E-05 | 7.264293 |
| 549 | Nudt4 | 0.658495 | 11.83317 | 4.996822 | 0.000154 | 0.00187 | 0.660952 |
| 550 | Gm6030 | 0.658047 | 14.79986 | 7.544252 | 1.63E-06 | 6.61E-05 | 5.290474 |
| 551 | Fbxo39 | 0.657903 | 5.395104 | 10.64818 | 1.93E-08 | 2.80E-06 | 9.785019 |
| 552 | Zfp72 | 0.657407 | 7.162308 | 5.667541 | 4.27E-05 | 0.000725 | 1.963375 |
| 553 | Dner | 0.657071 | 11.69937 | 4.718731 | 0.000266 | 0.002831 | 0.106415 |
| 554 | E030019B06Rik | 0.657025 | 5.911364 | 2.420785 | 0.028466 | 0.102695 | -4.50198 |
| 555 | Adam32 | 0.656519 | 5.935472 | 5.560873 | 5.21E-05 | 0.000835 | 1.759896 |
| 556 | Agpat9 | 0.655607 | 8.722262 | 2.997379 | 0.008926 | 0.041874 | -3.39256 |
| 557 | Cyp2r1 | 0.653779 | 6.424535 | 13.26151 | 9.37E-10 | 2.75E-07 | 12.78957 |
| 558 | LOC654469 | 0.653773 | 7.206755 | 4.255558 | 0.000674 | 0.005697 | -0.83149 |
| 559 | Vat1l | 0.653726 | 11.37003 | 2.36971 | 0.031464 | 0.110754 | -4.59579 |
| 560 | Lama4 | 0.653718 | 6.84334 | 2.545046 | 0.022264 | 0.085217 | -4.27022 |
| 561 | Lepr | 0.653433 | 6.101715 | 4.857043 | 0.000202 | 0.002306 | 0.383159 |
| 562 | Coil | 0.65335 | 9.932489 | 8.431713 | 4.11E-07 | 2.40E-05 | 6.69605 |
| 563 | Kcnh5 | 0.652269 | 7.490817 | 4.121334 | 0.000885 | 0.007024 | -1.10567 |
| 564 | Gm10144 | 0.651929 | 7.996599 | 5.492551 | 5.93E-05 | 0.000913 | 1.62881 |
| 565 | Gm7303 | 0.651351 | 6.079357 | 8.15997 | 6.20E-07 | 3.28E-05 | 6.27668 |
| 566 | 4930404H24Rik | 0.651091 | 5.700824 | 3.74675 | 0.001908 | 0.012552 | -1.87321 |
| 567 | Kcna7 | 0.650622 | 7.266276 | 7.704699 | 1.26E-06 | 5.46E-05 | 5.552362 |
| 568 | Ephx2 | 0.650137 | 9.369637 | 5.061841 | 0.000135 | 0.001691 | 0.789481 |
| 569 | Gpr6 | 0.649857 | 6.245735 | 3.615707 | 0.002499 | 0.015556 | -2.1416 |
| 570 | Cds1 | 0.648913 | 9.402227 | 2.621998 | 0.019095 | 0.07569 | -4.12439 |
| 571 | St6galnac1 | 0.648879 | 7.204171 | 3.453938 | 0.003489 | 0.020095 | -2.47203 |
| 572 | Lamc3 | 0.648661 | 7.693237 | 4.053064 | 0.001018 | 0.007815 | -1.24539 |
| 573 | Zswim1 | 0.648148 | 7.834091 | 6.903976 | 4.71E-06 | 0.000147 | 4.21021 |
| 574 | Raet1b | 0.647807 | 6.258165 | 6.202447 | 1.60E-05 | 0.00036 | 2.961146 |
| 575 | Haao | 0.64688 | 5.592531 | 3.875555 | 0.001464 | 0.010265 | -1.60914 |
| 576 | Fat2 | 0.646861 | 5.81852 | 3.0503 | 0.008009 | 0.038453 | -3.28719 |
| 577 | Gm5451 | 0.646751 | 13.60454 | 11.71244 | 5.27E-09 | 1.07E-06 | 11.08224 |
| 578 | Ddit4l | 0.645948 | 8.768106 | 3.13474 | 0.006735 | 0.033628 | -3.11821 |
| 579 | Gm5048 | 0.645124 | 11.75211 | 7.788098 | 1.11E-06 | 5.01E-05 | 5.687115 |
| 580 | Adrm1 | 0.644644 | 11.63718 | 7.779559 | 1.12E-06 | 5.05E-05 | 5.673359 |
| 581 | 2610318N02Rik | 0.644597 | 6.724913 | 6.047502 | 2.12E-05 | 0.000441 | 2.676084 |
| 582 | Ppp2r5e | 0.644407 | 7.723573 | 6.320477 | 1.30E-05 | 0.000309 | 3.176081 |
| 583 | Tcfap4 | 0.643768 | 5.248559 | 4.364211 | 0.000541 | 0.004787 | -0.61018 |
| 584 | Gm4953 | 0.64335 | 13.79154 | 21.30245 | 1.00E-12 | 1.87E-09 | 19.22066 |
| 585 | Olfr1229 | 0.642211 | 7.770894 | 8.093408 | 6.87E-07 | 3.50E-05 | 6.172496 |
| 586 | Serpinb6c | 0.64172 | 10.49182 | 7.353609 | 2.23E-06 | 8.46E-05 | 4.974739 |
| 587 | Hrh4 | 0.641233 | 4.955846 | 4.826269 | 0.000215 | 0.002402 | 0.321739 |
| 588 | Ntsr1 | 0.640968 | 6.570896 | 3.240152 | 0.005422 | 0.028284 | -2.90595 |
| 589 | Rab36 | 0.638687 | 7.439217 | 5.497593 | 5.88E-05 | 0.000908 | 1.638502 |
| 590 | Gm8759 | 0.638024 | 15.67375 | 10.91357 | 1.38E-08 | 2.18E-06 | 10.11881 |
| 591 | Olfr521 | 0.637197 | 5.602832 | 5.864831 | 2.96E-05 | 0.000557 | 2.335833 |
| 592 | Lsp1 | 0.634272 | 8.78646 | 3.675926 | 0.002208 | 0.014119 | -2.01832 |
| 593 | Dkc1 | 0.632205 | 7.404827 | 9.309333 | 1.15E-07 | 1.00E-05 | 7.987166 |
| 594 | Gm14378 | 0.631684 | 11.92638 | 7.764511 | 1.15E-06 | 5.12E-05 | 5.649098 |
| 595 | Gm3561 | 0.631527 | 4.954775 | 6.803127 | 5.60E-06 | 0.000164 | 4.034872 |
| 596 | Kpna4 | 0.63095 | 10.27868 | 5.813443 | 3.26E-05 | 0.000597 | 2.239311 |
| 597 | 1700024P12Rik | 0.630627 | 8.672055 | 6.030904 | 2.19E-05 | 0.00045 | 2.645353 |
| 598 | Radil | 0.630567 | 7.636619 | 7.489344 | 1.79E-06 | 7.13E-05 | 5.200048 |
| 599 | Sct | 0.630485 | 6.096049 | 3.565069 | 0.002775 | 0.01691 | -2.24517 |
| 600 | Zfp692 | 0.630052 | 10.76109 | 7.751376 | 1.17E-06 | 5.16E-05 | 5.627895 |
| 601 | Aym1 | 0.629617 | 6.534259 | 5.089631 | 0.000128 | 0.001621 | 0.844277 |
| 602 | Gm438 | 0.629558 | 4.96789 | 5.541812 | 5.41E-05 | 0.000855 | 1.723383 |
| 603 | Gm10639 | 0.628906 | 5.105503 | 2.933697 | 0.010166 | 0.046232 | -3.51873 |
| 604 | Dbt | 0.628262 | 6.824532 | 6.976446 | 4.17E-06 | 0.000134 | 4.335332 |
| 605 | Gm4858 | 0.627964 | 7.3392 | 5.71911 | 3.88E-05 | 0.000678 | 2.061222 |
| 606 | Zfp37 | 0.627922 | 7.109527 | 8.438378 | 4.06E-07 | 2.39E-05 | 6.706216 |
| 607 | 2310040G24Rik | 0.627915 | 7.447218 | 7.893175 | 9.38E-07 | 4.45E-05 | 5.855564 |
| 608 | Vill | 0.627874 | 4.948857 | 2.610194 | 0.019551 | 0.076973 | -4.14687 |
| 609 | Olfr173 | 0.627312 | 9.49339 | 2.599138 | 0.019988 | 0.078329 | -4.16788 |
| 610 | Gucy1b2 | 0.624608 | 6.014796 | 5.785706 | 3.43E-05 | 0.000622 | 2.18707 |
| 611 | Olfr544 | 0.623826 | 10.88927 | 7.626827 | 1.43E-06 | 5.99E-05 | 5.425692 |
| 612 | Ankrd56 | 0.623376 | 5.540894 | 3.577495 | 0.002704 | 0.01656 | -2.21976 |
| 613 | Krtap10-4 | 0.621078 | 4.948815 | 9.475418 | 9.12E-08 | 8.51E-06 | 8.221069 |
| 614 | Zmat3 | 0.620844 | 11.23815 | 10.86469 | 1.47E-08 | 2.25E-06 | 10.05786 |
| 615 | Lrch3 | 0.620682 | 5.389687 | 3.109992 | 0.007086 | 0.034993 | -3.16784 |
| 616 | Tmem174 | 0.620512 | 6.137609 | 8.058189 | 7.25E-07 | 3.65E-05 | 6.117135 |
| 617 | Gtf2h5 | 0.620262 | 12.57207 | 13.15089 | 1.05E-09 | 3.00E-07 | 12.67412 |
| 618 | Gm4211 | 0.620161 | 5.419722 | 2.603917 | 0.019798 | 0.077764 | -4.1588 |
| 619 | Arhgap25 | 0.619447 | 8.626085 | 3.279718 | 0.004998 | 0.026587 | -2.82595 |
| 620 | LOC100044874 | 0.61941 | 7.283496 | 4.890295 | 0.000189 | 0.002199 | 0.449421 |
| 621 | Gm5797 | 0.618876 | 8.887667 | 8.03138 | 7.56E-07 | 3.75E-05 | 6.074884 |
| 622 | Padi2 | 0.618722 | 9.091103 | 3.847103 | 0.001552 | 0.010724 | -1.66748 |
| 623 | Astn2 | 0.618363 | 6.337294 | 2.930414 | 0.010234 | 0.046474 | -3.52522 |
| 624 | Plk2 | 0.617047 | 10.92995 | 2.960519 | 0.009624 | 0.044332 | -3.46568 |
| 625 | Sytl1 | 0.616922 | 6.410143 | 3.576728 | 0.002709 | 0.016577 | -2.22133 |
| 626 | Has1 | 0.616111 | 7.495937 | 3.235712 | 0.005472 | 0.028497 | -2.91492 |
| 627 | Myl6b | 0.61596 | 11.45304 | 3.806545 | 0.001687 | 0.011441 | -1.75063 |
| 628 | Gm11362 | 0.615905 | 12.13647 | 9.758343 | 6.19E-08 | 6.50E-06 | 8.612204 |
| 629 | Car13 | 0.613596 | 4.938157 | 2.482825 | 0.025189 | 0.093567 | -4.38688 |
| 630 | Pphln1 | 0.61355 | 7.830693 | 7.33601 | 2.29E-06 | 8.62E-05 | 4.945341 |
| 631 | Ccdc129 | 0.613543 | 6.438067 | 2.734777 | 0.015222 | 0.063309 | -3.90781 |
| 632 | Cdyl2 | 0.613241 | 7.489473 | 5.932829 | 2.61E-05 | 0.000513 | 2.463013 |
| 633 | Mrpl1 | 0.612779 | 7.591399 | 5.614898 | 4.71E-05 | 0.000777 | 1.863135 |
| 634 | Elmo1 | 0.612716 | 5.253522 | 2.580544 | 0.020744 | 0.080563 | -4.20316 |
| 635 | Olfr97 | 0.612424 | 4.9472 | 2.183357 | 0.045108 | 0.145897 | -4.92992 |
| 636 | Ptger3 | 0.612175 | 5.054486 | 4.195161 | 0.000762 | 0.006264 | -0.95477 |
| 637 | Smok3b | 0.611743 | 4.991267 | 20.44656 | 1.84E-12 | 2.89E-09 | 18.68204 |
| 638 | Pou3f2 | 0.611701 | 7.134267 | 3.196373 | 0.005933 | 0.030468 | -2.99426 |
| 639 | Vip | 0.608792 | 8.973067 | 3.006358 | 0.008763 | 0.041241 | -3.37472 |
| 640 | BC013529 | 0.607556 | 5.253694 | 3.226422 | 0.005578 | 0.028971 | -2.93367 |
| 641 | Vim | 0.606938 | 11.13847 | 3.244377 | 0.005375 | 0.028119 | -2.89741 |
| 642 | Akap7 | 0.606804 | 10.44108 | 7.323654 | 2.34E-06 | 8.68E-05 | 4.924675 |
| 643 | Tbca | 0.606575 | 11.50388 | 9.843892 | 5.52E-08 | 6.08E-06 | 8.728691 |
| 644 | Wbscr22 | 0.606569 | 10.17924 | 8.408062 | 4.25E-07 | 2.45E-05 | 6.659927 |
| 645 | Ifi27l2a | 0.605596 | 8.524855 | 3.150573 | 0.006519 | 0.032759 | -3.08641 |
| 646 | Slc45a3 | 0.605348 | 6.8918 | 14.39945 | 2.93E-10 | 1.22E-07 | 13.92453 |
| 647 | Cyp4f18 | 0.604901 | 5.92385 | 5.158785 | 0.000112 | 0.001471 | 0.980267 |
| 648 | Prss28 | 0.604377 | 4.948813 | 2.144272 | 0.048594 | 0.154408 | -4.99824 |
| 649 | Fam178b | 0.603502 | 5.686144 | 5.076999 | 0.000132 | 0.001648 | 0.819379 |
| 650 | Prb1 | 0.603453 | 5.209751 | 6.169691 | 1.70E-05 | 0.000375 | 2.901155 |
| 651 | Ppargc1b | 0.603359 | 7.071578 | 7.667355 | 1.34E-06 | 5.68E-05 | 5.491718 |
| 652 | Pdcl3 | 0.603059 | 8.115443 | 4.231299 | 0.000708 | 0.005938 | -0.88099 |
| 653 | Lypd5 | 0.603007 | 6.467596 | 3.16833 | 0.006286 | 0.03189 | -3.05072 |
| 654 | Gm10653 | 0.602957 | 16.12698 | 10.29792 | 3.03E-08 | 3.92E-06 | 9.33346 |
| 655 | Mup4 | 0.6015 | 4.969282 | 5.088399 | 0.000129 | 0.001624 | 0.841849 |
| 656 | Zfp438 | 0.600931 | 5.273733 | 3.438735 | 0.003601 | 0.020607 | -2.503 |
| 657 | Eif3j | 0.600692 | 9.577656 | 9.394626 | 1.02E-07 | 9.10E-06 | 8.107689 |
| 658 | Srp54a | 0.600195 | 8.678075 | 7.317654 | 2.36E-06 | 8.75E-05 | 4.914632 |
| 659 | Bmp7 | 0.599303 | 9.870941 | 2.96191 | 0.009597 | 0.044256 | -3.46292 |
| 660 | Gm6712 | 0.598641 | 7.169497 | 6.456429 | 1.02E-05 | 0.000256 | 3.421265 |
| 661 | Gpr157 | 0.597408 | 11.92797 | 3.873984 | 0.001469 | 0.010286 | -1.61236 |
| 662 | Barhl1 | 0.597167 | 6.111402 | 2.856548 | 0.011895 | 0.05221 | -3.6706 |
| 663 | Fnbp1l | 0.596878 | 10.56272 | 2.695819 | 0.016465 | 0.067428 | -3.98298 |
| 664 | F9 | 0.594483 | 5.054057 | 5.182799 | 0.000107 | 0.001423 | 1.027364 |
| 665 | C1rl | 0.594168 | 5.526248 | 3.271893 | 0.005079 | 0.026923 | -2.84179 |
| 666 | Anpep | 0.593949 | 8.703459 | 2.688467 | 0.016711 | 0.068246 | -3.99713 |
| 667 | Snx24 | 0.59394 | 7.176545 | 4.361668 | 0.000544 | 0.004807 | -0.61535 |
| 668 | Gap43 | 0.59339 | 13.72715 | 3.007851 | 0.008736 | 0.04115 | -3.37175 |
| 669 | Ifi44 | 0.592763 | 4.943158 | 3.292777 | 0.004865 | 0.026036 | -2.79952 |
| 670 | Sf3a2 | 0.591869 | 12.5353 | 4.843839 | 0.000208 | 0.002347 | 0.356817 |
| 671 | Lgi2 | 0.591413 | 10.26139 | 3.009119 | 0.008714 | 0.041087 | -3.36922 |
| 672 | Asap1 | 0.591192 | 10.08711 | 4.236598 | 0.0007 | 0.005886 | -0.87017 |
| 673 | C1qtnf1 | 0.589907 | 5.579006 | 3.108123 | 0.007113 | 0.035065 | -3.17158 |
| 674 | Otud1 | 0.588632 | 5.912528 | 3.082371 | 0.007499 | 0.036574 | -3.22313 |
| 675 | Serpina11 | 0.58772 | 6.14438 | 3.531983 | 0.00297 | 0.017785 | -2.31278 |
| 676 | Gm684 | 0.586951 | 4.979674 | 2.386115 | 0.03047 | 0.107987 | -4.56575 |
| 677 | Cep57l1 | 0.586618 | 6.547044 | 7.168425 | 3.02E-06 | 0.000104 | 4.663257 |
| 678 | Ipo8 | 0.585709 | 9.848776 | 13.82851 | 5.20E-10 | 1.87E-07 | 13.36677 |
| 679 | Pnpla8 | 0.585331 | 7.298867 | 4.83145 | 0.000213 | 0.002386 | 0.332085 |
| 680 | Gm5145 | -0.58525 | 5.51201 | -4.84136 | 0.000209 | 0.002353 | 0.351871 |
| 681 | A230070E04Rik | -0.58633 | 9.661529 | -5.75284 | 3.64E-05 | 0.000649 | 2.125031 |
| 682 | Papolg | -0.58733 | 9.49431 | -2.16861 | 0.046395 | 0.148944 | -4.95576 |
| 683 | F830223B06Rik | -0.5875 | 5.02334 | -2.75922 | 0.014489 | 0.060915 | -3.86046 |
| 684 | Hsph1 | -0.58802 | 13.86654 | -4.2849 | 0.000635 | 0.005422 | -0.77167 |
| 685 | Sema5a | -0.58819 | 12.0507 | -2.70306 | 0.016227 | 0.066659 | -3.96904 |
| 686 | Csrnp1 | -0.58846 | 10.2557 | -3.378 | 0.004081 | 0.022658 | -2.62659 |
| 687 | Epha6 | -0.58961 | 9.654393 | -3.63067 | 0.002423 | 0.015186 | -2.11097 |
| 688 | Cant1 | -0.58968 | 6.021027 | -3.24248 | 0.005396 | 0.028209 | -2.90124 |
| 689 | Atoh8 | -0.59009 | 5.346164 | -2.42814 | 0.028058 | 0.101471 | -4.48841 |
| 690 | Tbc1d8b | -0.59082 | 8.198113 | -4.26446 | 0.000662 | 0.005612 | -0.81333 |
| 691 | B3gat2 | -0.59151 | 8.533558 | -3.51765 | 0.00306 | 0.018185 | -2.34204 |
| 692 | Rrad | -0.5918 | 5.161068 | -2.50733 | 0.023996 | 0.090201 | -4.34108 |
| 693 | Faah | -0.59254 | 9.13469 | -4.0002 | 0.001134 | 0.008486 | -1.35367 |
| 694 | Adamts9 | -0.59314 | 8.132724 | -4.20664 | 0.000744 | 0.006152 | -0.93133 |
| 695 | Syt3 | -0.59396 | 10.69387 | -7.27567 | 2.53E-06 | 9.22E-05 | 4.844221 |
| 696 | Chordc1 | -0.59411 | 11.27786 | -7.32653 | 2.33E-06 | 8.66E-05 | 4.929487 |
| 697 | Il18bp | -0.59508 | 6.013991 | -3.42542 | 0.003701 | 0.021033 | -2.53012 |
| 698 | 1810041L15Rik | -0.59555 | 9.212597 | -5.37727 | 7.38E-05 | 0.001072 | 1.406321 |
| 699 | Rfx3 | -0.59563 | 7.048985 | -4.47131 | 0.000436 | 0.00407 | -0.39274 |
| 700 | Zfp12 | -0.59572 | 6.077348 | -7.50857 | 1.73E-06 | 6.93E-05 | 5.231752 |
| 701 | Amigo2 | -0.59574 | 7.656381 | -3.02557 | 0.008425 | 0.039975 | -3.33648 |
| 702 | Casp6 | -0.59587 | 8.684804 | -4.17371 | 0.000796 | 0.006467 | -0.99858 |
| 703 | Klhl34 | -0.59631 | 6.637066 | -4.81321 | 0.000221 | 0.002439 | 0.295642 |
| 704 | Nrip3 | -0.59693 | 14.42933 | -4.05606 | 0.001012 | 0.007787 | -1.23926 |
| 705 | Trim68 | -0.59706 | 7.980607 | -6.03836 | 2.16E-05 | 0.000446 | 2.659155 |
| 706 | Atp6v1g2 | -0.59746 | 12.83608 | -6.97727 | 4.16E-06 | 0.000134 | 4.336752 |
| 707 | Ccdc88b | -0.59782 | 9.516716 | -3.73753 | 0.001945 | 0.012732 | -1.89211 |
| 708 | Ankrd5 | -0.59803 | 5.039679 | -5.94028 | 2.58E-05 | 0.000508 | 2.476916 |
| 709 | Enox2 | -0.59838 | 9.403181 | -4.1419 | 0.000849 | 0.006792 | -1.06362 |
| 710 | Zfyve1 | -0.59854 | 9.109716 | -8.72333 | 2.66E-07 | 1.77E-05 | 7.135596 |
| 711 | Slit1 | -0.59905 | 13.77893 | -2.7154 | 0.015829 | 0.065376 | -3.94525 |
| 712 | Neil1 | -0.59989 | 6.000484 | -6.06486 | 2.06E-05 | 0.000431 | 2.708174 |
| 713 | Npy2r | -0.6001 | 8.619398 | -2.47655 | 0.025503 | 0.094418 | -4.39859 |
| 714 | BC046331 | -0.60026 | 6.895409 | -6.66254 | 7.13E-06 | 0.000196 | 3.788074 |
| 715 | Elmo2 | -0.60052 | 8.472231 | -5.55524 | 5.27E-05 | 0.00084 | 1.749103 |
| 716 | Tmod4 | -0.60138 | 7.045999 | -3.74976 | 0.001896 | 0.012497 | -1.86703 |
| 717 | 2900005J15Rik | -0.60207 | 5.154053 | -5.17724 | 0.000108 | 0.001436 | 1.016459 |
| 718 | Nrn1 | -0.60235 | 13.48205 | -7.38018 | 2.13E-06 | 8.17E-05 | 5.019047 |
| 719 | Grik4 | -0.60257 | 13.05895 | -2.45689 | 0.026513 | 0.097223 | -4.43514 |
| 720 | Pstpip1 | -0.60292 | 5.634654 | -3.15385 | 0.006476 | 0.032629 | -3.07984 |
| 721 | Ints4 | -0.60318 | 7.657234 | -4.88374 | 0.000192 | 0.002219 | 0.436358 |
| 722 | Chst11 | -0.60364 | 9.781868 | -5.69144 | 4.08E-05 | 0.000703 | 2.008759 |
| 723 | Rala | -0.60441 | 10.84583 | -2.79261 | 0.013542 | 0.057684 | -3.79555 |
| 724 | LOC100504423 | -0.6046 | 11.24305 | -5.70304 | 4.00E-05 | 0.000694 | 2.030758 |
| 725 | Prkdc | -0.60469 | 8.705391 | -8.447 | 4.01E-07 | 2.36E-05 | 6.719353 |
| 726 | Bcas3 | -0.60551 | 7.651585 | -7.60275 | 1.49E-06 | 6.16E-05 | 5.386361 |
| 727 | Tuba8 | -0.60573 | 10.6011 | -2.64385 | 0.018277 | 0.073318 | -4.08268 |
| 728 | 1700061G19Rik | -0.60576 | 5.501821 | -5.51342 | 5.70E-05 | 0.00089 | 1.668909 |
| 729 | 5430417L22Rik | -0.60611 | 7.825445 | -5.33175 | 8.05E-05 | 0.001146 | 1.318019 |
| 730 | Hist1h3d | -0.60651 | 13.07062 | -4.32847 | 0.000581 | 0.005078 | -0.68292 |
| 731 | 1110017D15Rik | -0.60711 | 8.371722 | -3.63228 | 0.002415 | 0.01514 | -2.10767 |
| 732 | Vav3 | -0.6079 | 6.637432 | -2.90698 | 0.010735 | 0.048116 | -3.57146 |
| 733 | Atf6b | -0.60795 | 9.373016 | -5.58616 | 4.97E-05 | 0.000807 | 1.808261 |
| 734 | Dgkb | -0.60963 | 12.27372 | -4.99743 | 0.000154 | 0.001868 | 0.662153 |
| 735 | Cd38 | -0.61036 | 7.803175 | -3.66686 | 0.002249 | 0.014333 | -2.03688 |
| 736 | Fkbp4 | -0.61141 | 10.09468 | -9.12715 | 1.49E-07 | 1.16E-05 | 7.726859 |
| 737 | Dpyd | -0.61193 | 8.625412 | -5.28645 | 8.78E-05 | 0.001228 | 1.229887 |
| 738 | Cpne7 | -0.61206 | 15.45982 | -3.37874 | 0.004075 | 0.022645 | -2.6251 |
| 739 | 1500015A07Rik | -0.61312 | 8.334556 | -2.4905 | 0.024809 | 0.092516 | -4.37256 |
| 740 | 7530428D23Rik | -0.61327 | 5.255072 | -3.78925 | 0.001748 | 0.011739 | -1.78609 |
| 741 | Cd300lg | -0.61353 | 7.810974 | -4.29643 | 0.00062 | 0.005327 | -0.74817 |
| 742 | Slc29a4 | -0.61419 | 12.52015 | -3.58913 | 0.00264 | 0.016249 | -2.19597 |
| 743 | Pnmal2 | -0.61436 | 11.99582 | -8.2717 | 5.23E-07 | 2.87E-05 | 6.450257 |
| 744 | Pcgf6 | -0.61514 | 9.281785 | -7.03529 | 3.78E-06 | 0.000125 | 4.436393 |
| 745 | Gpr1 | -0.61562 | 6.848943 | -3.80953 | 0.001677 | 0.011392 | -1.74452 |
| 746 | Rtn4ip1 | -0.6169 | 5.278182 | -5.99429 | 2.34E-05 | 0.000472 | 2.57744 |
| 747 | Nol9 | -0.61736 | 7.408181 | -6.08755 | 1.97E-05 | 0.000418 | 2.750074 |
| 748 | Orai2 | -0.61747 | 11.53117 | -3.52175 | 0.003034 | 0.018091 | -2.33369 |
| 749 | Capn5 | -0.61775 | 9.086659 | -3.91486 | 0.001351 | 0.009702 | -1.52856 |
| 750 | 1700121C10Rik | -0.61782 | 6.326242 | -3.36507 | 0.004191 | 0.023135 | -2.65287 |
| 751 | Cpne4 | -0.618 | 10.696 | -3.7007 | 0.002098 | 0.013518 | -1.96758 |
| 752 | Gp5 | -0.61909 | 5.369299 | -2.97442 | 0.009355 | 0.043412 | -3.43813 |
| 753 | Mas1 | -0.61911 | 11.36681 | -3.48573 | 0.003268 | 0.019129 | -2.40719 |
| 754 | Grpel2 | -0.62006 | 7.663465 | -3.47505 | 0.003341 | 0.019447 | -2.42898 |
| 755 | Sdccag8 | -0.62013 | 7.855748 | -3.39991 | 0.003901 | 0.021937 | -2.58204 |
| 756 | Znrf3 | -0.62047 | 5.854028 | -5.05381 | 0.000138 | 0.001714 | 0.773639 |
| 757 | Rogdi | -0.62058 | 11.70112 | -5.31027 | 8.39E-05 | 0.001184 | 1.276266 |
| 758 | Gabpb2 | -0.62106 | 6.086075 | -3.35816 | 0.004252 | 0.023408 | -2.66691 |
| 759 | Slc38a7 | -0.62107 | 8.962265 | -6.9292 | 4.52E-06 | 0.000142 | 4.253837 |
| 760 | Supt6h | -0.62119 | 10.34015 | -4.81783 | 0.000219 | 0.00243 | 0.304875 |
| 761 | Map2k1 | -0.62187 | 13.75823 | -6.47136 | 9.95E-06 | 0.000252 | 3.448033 |
| 762 | Twist2 | -0.62237 | 6.781631 | -2.16275 | 0.046916 | 0.150201 | -4.96602 |
| 763 | Lsm11 | -0.62246 | 6.78957 | -3.06331 | 0.007798 | 0.037674 | -3.26122 |
| 764 | Dntt | -0.62276 | 6.232895 | -3.80938 | 0.001677 | 0.011392 | -1.74482 |
| 765 | Hpca | -0.6239 | 15.51839 | -2.95803 | 0.009673 | 0.044487 | -3.47061 |
| 766 | Dock11 | -0.62491 | 9.416905 | -4.7401 | 0.000255 | 0.002736 | 0.14929 |
| 767 | Fam123c | -0.62524 | 10.46754 | -4.17398 | 0.000795 | 0.006467 | -0.99803 |
| 768 | Tcerg1l | -0.6257 | 7.581605 | -3.57258 | 0.002732 | 0.016693 | -2.22982 |
| 769 | Btbd9 | -0.62616 | 12.2938 | -6.08556 | 1.98E-05 | 0.000419 | 2.746399 |
| 770 | Srgap3 | -0.62665 | 10.71333 | -6.95201 | 4.34E-06 | 0.000138 | 4.29322 |
| 771 | Slc5a10 | -0.62692 | 5.368739 | -4.08183 | 0.00096 | 0.007492 | -1.1865 |
| 772 | Treml4 | -0.62788 | 5.085957 | -4.49683 | 0.000414 | 0.003908 | -0.34103 |
| 773 | Ccdc37 | -0.62887 | 6.522591 | -2.92619 | 0.010322 | 0.046714 | -3.53357 |
| 774 | Tbata | -0.62905 | 7.260927 | -3.18747 | 0.006043 | 0.03088 | -3.0122 |
| 775 | Neurod2 | -0.62925 | 13.80767 | -3.76321 | 0.001844 | 0.012229 | -1.83947 |
| 776 | Tmprss11bnl | -0.62978 | 5.921692 | -8.20233 | 5.81E-07 | 3.11E-05 | 6.342684 |
| 777 | Lgi3 | -0.63018 | 11.64664 | -6.73179 | 6.33E-06 | 0.000179 | 3.909993 |
| 778 | Cdhr1 | -0.63093 | 8.474175 | -3.66192 | 0.002272 | 0.014444 | -2.04701 |
| 779 | Lrrc46 | -0.63143 | 5.463768 | -4.69529 | 0.000279 | 0.002921 | 0.059333 |
| 780 | Tmem138 | -0.63234 | 6.569962 | -4.34783 | 0.000559 | 0.004924 | -0.64351 |
| 781 | Zgpat | -0.63278 | 8.645293 | -8.71806 | 2.68E-07 | 1.77E-05 | 7.127759 |
| 782 | Rere | -0.63337 | 5.812888 | -3.98084 | 0.00118 | 0.008734 | -1.39332 |
| 783 | Ahcy | -0.63342 | 6.147786 | -4.94098 | 0.000172 | 0.002044 | 0.550215 |
| 784 | Klhl3 | -0.63408 | 5.731094 | -3.76081 | 0.001854 | 0.012271 | -1.84438 |
| 785 | Mast3 | -0.63473 | 9.58625 | -3.42683 | 0.00369 | 0.020993 | -2.52724 |
| 786 | Stk33 | -0.63525 | 6.438066 | -3.79209 | 0.001738 | 0.011696 | -1.78026 |
| 787 | Klk1b24 | -0.63566 | 5.510101 | -2.7558 | 0.014589 | 0.061268 | -3.86709 |
| 788 | Gm3146 | -0.63593 | 5.752513 | -3.58156 | 0.002682 | 0.016458 | -2.21145 |
| 789 | Nbeal2 | -0.63611 | 6.747853 | -5.66784 | 4.27E-05 | 0.000725 | 1.963941 |
| 790 | Anks3 | -0.63663 | 7.860926 | -4.5201 | 0.000395 | 0.003779 | -0.29393 |
| 791 | Dmp1 | -0.63688 | 5.392396 | -3.81281 | 0.001665 | 0.011339 | -1.73778 |
| 792 | Psme3 | -0.63723 | 8.574092 | -5.62534 | 4.62E-05 | 0.000768 | 1.883038 |
| 793 | Dusp19 | -0.63771 | 5.353371 | -5.29822 | 8.59E-05 | 0.001204 | 1.252817 |
| 794 | Fbrs | -0.63829 | 7.624979 | -3.87864 | 0.001455 | 0.010227 | -1.60281 |
| 795 | Prkg1 | -0.63855 | 8.959881 | -6.85392 | 5.13E-06 | 0.000155 | 4.123367 |
| 796 | B3galt5 | -0.63866 | 11.64745 | -3.60029 | 0.00258 | 0.015955 | -2.17314 |
| 797 | Il17rd | -0.64045 | 8.851198 | -2.64595 | 0.0182 | 0.07313 | -4.07866 |
| 798 | Prtg | -0.64149 | 7.148846 | -4.48012 | 0.000428 | 0.004012 | -0.37487 |
| 799 | F730043M19Rik | -0.64204 | 7.841169 | -4.20275 | 0.00075 | 0.006189 | -0.93928 |
| 800 | Napepld | -0.64231 | 11.38866 | -4.20973 | 0.00074 | 0.006128 | -0.92502 |
| 801 | Tgfb2 | -0.64281 | 11.27341 | -3.37249 | 0.004128 | 0.022864 | -2.6378 |
| 802 | Mprip | -0.64384 | 11.61395 | -2.38925 | 0.030283 | 0.107457 | -4.56001 |
| 803 | Wipf3 | -0.64399 | 15.0409 | -3.58543 | 0.00266 | 0.016364 | -2.20354 |
| 804 | Spint1 | -0.64585 | 5.760313 | -5.04461 | 0.00014 | 0.001741 | 0.755459 |
| 805 | Tcp11 | -0.6464 | 6.192481 | -2.62698 | 0.018905 | 0.075059 | -4.11488 |
| 806 | Evc2 | -0.64762 | 8.226212 | -4.21465 | 0.000732 | 0.006087 | -0.91496 |
| 807 | Pion | -0.6486 | 6.628238 | -2.52255 | 0.023282 | 0.088088 | -4.31254 |
| 808 | Ddn | -0.64923 | 12.96852 | -3.61455 | 0.002505 | 0.015584 | -2.14397 |
| 809 | Tecr | -0.65032 | 13.87371 | -5.91795 | 2.69E-05 | 0.000522 | 2.435235 |
| 810 | Cd109 | -0.65078 | 5.450347 | -2.31727 | 0.034849 | 0.119838 | -4.69113 |
| 811 | Impg1 | -0.65103 | 10.01682 | -5.93209 | 2.62E-05 | 0.000513 | 2.461639 |
| 812 | Clmn | -0.65105 | 11.8259 | -7.26482 | 2.58E-06 | 9.37E-05 | 4.825983 |
| 813 | Hpdl | -0.6513 | 8.813895 | -3.13743 | 0.006698 | 0.033495 | -3.11281 |
| 814 | Cpne6 | -0.65257 | 16.14142 | -5.07865 | 0.000131 | 0.001645 | 0.822637 |
| 815 | Mta3 | -0.65327 | 8.305047 | -2.29843 | 0.036147 | 0.12334 | -4.72515 |
| 816 | Gss | -0.65341 | 9.315952 | -3.76136 | 0.001851 | 0.012265 | -1.84327 |
| 817 | Atp2b3 | -0.65365 | 11.37777 | -6.03722 | 2.16E-05 | 0.000446 | 2.657045 |
| 818 | Fance | -0.65371 | 10.11921 | -2.55823 | 0.021688 | 0.083416 | -4.24536 |
| 819 | Itga11 | -0.65448 | 8.983079 | -3.15656 | 0.00644 | 0.03252 | -3.07438 |
| 820 | Nrp2 | -0.6566 | 11.82249 | -5.60015 | 4.84E-05 | 0.000793 | 1.834997 |
| 821 | Tsga10 | -0.65735 | 5.707964 | -7.76585 | 1.15E-06 | 5.12E-05 | 5.651259 |
| 822 | Mt3 | -0.6585 | 12.97537 | -10.801 | 1.59E-08 | 2.40E-06 | 9.978039 |
| 823 | Immt | -0.65863 | 11.57926 | -3.76577 | 0.001835 | 0.012176 | -1.83421 |
| 824 | Col27a1 | -0.65901 | 5.547919 | -5.10852 | 0.000124 | 0.001581 | 0.881483 |
| 825 | Gm11744 | -0.6609 | 8.834985 | -2.35823 | 0.032177 | 0.112707 | -4.61674 |
| 826 | Pex10 | -0.66173 | 8.778392 | -8.98544 | 1.82E-07 | 1.33E-05 | 7.521643 |
| 827 | Onecut2 | -0.66257 | 9.889559 | -4.12252 | 0.000883 | 0.007012 | -1.10325 |
| 828 | Cela1 | -0.66316 | 6.753084 | -6.45063 | 1.03E-05 | 0.000259 | 3.410855 |
| 829 | Gatsl3 | -0.66433 | 5.333358 | -6.44474 | 1.04E-05 | 0.00026 | 3.40028 |
| 830 | Acot5 | -0.66811 | 9.511733 | -2.8009 | 0.013317 | 0.056878 | -3.77941 |
| 831 | Myo10 | -0.66892 | 7.642887 | -2.51064 | 0.023839 | 0.089733 | -4.33488 |
| 832 | 1700013F07Rik | -0.67056 | 6.0197 | -2.85608 | 0.011906 | 0.05224 | -3.67152 |
| 833 | Bsn | -0.67174 | 13.35714 | -9.01311 | 1.75E-07 | 1.31E-05 | 7.561898 |
| 834 | Slc5a5 | -0.67233 | 5.963884 | -3.13919 | 0.006674 | 0.03342 | -3.10928 |
| 835 | Bid | -0.6731 | 9.485605 | -4.31533 | 0.000597 | 0.005173 | -0.70967 |
| 836 | Gpr39 | -0.67404 | 6.732736 | -4.10768 | 0.00091 | 0.007181 | -1.13359 |
| 837 | Aldh1a3 | -0.67472 | 6.056336 | -2.72171 | 0.015629 | 0.064708 | -3.93307 |
| 838 | Kbtbd11 | -0.67555 | 12.22802 | -8.01564 | 7.75E-07 | 3.82E-05 | 6.050029 |
| 839 | Hist1h2be | -0.67566 | 9.144926 | -3.85772 | 0.001519 | 0.010532 | -1.64571 |
| 840 | Idh3b | -0.67572 | 13.4122 | -6.79245 | 5.70E-06 | 0.000166 | 4.016229 |
| 841 | Pkp3 | -0.67613 | 12.24855 | -6.31244 | 1.32E-05 | 0.00031 | 3.161508 |
| 842 | Car12 | -0.6773 | 12.29658 | -4.12098 | 0.000886 | 0.007024 | -1.10639 |
| 843 | Pfkfb3 | -0.67738 | 8.205821 | -4.4561 | 0.000449 | 0.004158 | -0.42356 |
| 844 | Ptchd2 | -0.6774 | 6.797392 | -4.53595 | 0.000383 | 0.003694 | -0.26186 |
| 845 | Dalrd3 | -0.67787 | 8.902261 | -6.24248 | 1.49E-05 | 0.00034 | 3.034255 |
| 846 | Bves | -0.67837 | 8.689153 | -2.71895 | 0.015716 | 0.06497 | -3.93839 |
| 847 | Podxl2 | -0.67889 | 10.56038 | -2.23547 | 0.04082 | 0.13543 | -4.83783 |
| 848 | Tmem126b | -0.67906 | 9.196149 | -7.42586 | 1.98E-06 | 7.70E-05 | 5.094976 |
| 849 | Lrrc33 | -0.67906 | 10.3399 | -6.47862 | 9.82E-06 | 0.00025 | 3.461048 |
| 850 | Oma1 | -0.67979 | 6.573496 | -7.1329 | 3.21E-06 | 0.000109 | 4.602961 |
| 851 | LOC100505195 | -0.67988 | 7.470755 | -4.34599 | 0.000561 | 0.00494 | -0.64725 |
| 852 | Rnf128 | -0.68027 | 13.21398 | -4.37601 | 0.000528 | 0.004699 | -0.58619 |
| 853 | D630039A03Rik | -0.68059 | 5.831669 | -4.77678 | 0.000237 | 0.002581 | 0.222771 |
| 854 | Gm4371 | -0.68063 | 5.497588 | -3.29797 | 0.004813 | 0.025798 | -2.78899 |
| 855 | Atoh7 | -0.68129 | 5.815903 | -4.5041 | 0.000408 | 0.00387 | -0.32632 |
| 856 | Crat | -0.68166 | 5.904171 | -5.76262 | 3.58E-05 | 0.000641 | 2.143517 |
| 857 | Arhgef17 | -0.68167 | 12.38654 | -5.03117 | 0.000144 | 0.001776 | 0.728906 |
| 858 | Egr3 | -0.68199 | 4.962984 | -2.4502 | 0.026865 | 0.098122 | -4.44756 |
| 859 | Mcm6 | -0.68209 | 10.13446 | -4.15526 | 0.000826 | 0.006653 | -1.0363 |
| 860 | DXBay18 | -0.68338 | 8.594869 | -4.55084 | 0.000372 | 0.003616 | -0.23176 |
| 861 | Usp24 | -0.68529 | 7.248605 | -3.36028 | 0.004233 | 0.023335 | -2.6626 |
| 862 | Colec11 | -0.6861 | 6.341741 | -5.08254 | 0.00013 | 0.00164 | 0.830295 |
| 863 | Auts2 | -0.6865 | 9.889749 | -2.69262 | 0.016571 | 0.067796 | -3.98914 |
| 864 | 6330403A02Rik | -0.69038 | 8.527221 | -3.80753 | 0.001684 | 0.011423 | -1.74862 |
| 865 | Gm5639 | -0.69248 | 8.850122 | -4.36842 | 0.000536 | 0.004758 | -0.60162 |
| 866 | Atf6 | -0.69325 | 9.274478 | -8.63918 | 3.01E-07 | 1.93E-05 | 7.009862 |
| 867 | Dph1 | -0.6939 | 5.583917 | -7.21343 | 2.81E-06 | 9.98E-05 | 4.739388 |
| 868 | Limd2 | -0.69391 | 10.92401 | -3.85435 | 0.001529 | 0.010592 | -1.65261 |
| 869 | Dlx1as | -0.69454 | 5.169219 | -5.85076 | 3.04E-05 | 0.000568 | 2.309439 |
| 870 | Tead1 | -0.69682 | 6.803066 | -6.68418 | 6.87E-06 | 0.000191 | 3.826244 |
| 871 | Nes | -0.69769 | 8.146754 | -2.41924 | 0.028553 | 0.102957 | -4.50484 |
| 872 | Stab2 | -0.69815 | 5.787373 | -4.61193 | 0.000329 | 0.003299 | -0.10844 |
| 873 | Gm7420 | -0.7004 | 6.216119 | -2.87871 | 0.011371 | 0.050341 | -3.6271 |
| 874 | Prss35 | -0.70057 | 5.476257 | -3.0967 | 0.007282 | 0.035704 | -3.19446 |
| 875 | Fbxo33 | -0.70069 | 10.34433 | -5.57941 | 5.04E-05 | 0.000814 | 1.795366 |
| 876 | Hdc | -0.70091 | 6.741071 | -3.92364 | 0.001326 | 0.00956 | -1.51056 |
| 877 | 9030425E11Rik | -0.70135 | 8.850015 | -4.18807 | 0.000773 | 0.006327 | -0.96926 |
| 878 | Sec1 | -0.70203 | 5.253146 | -3.31866 | 0.004612 | 0.024952 | -2.74707 |
| 879 | Gm5640 | -0.70338 | 9.254649 | -4.48182 | 0.000427 | 0.004 | -0.37143 |
| 880 | 2410004A20Rik | -0.70714 | 7.327916 | -2.47215 | 0.025726 | 0.095065 | -4.40676 |
| 881 | Tdo2 | -0.70715 | 5.457107 | -2.35309 | 0.032502 | 0.113663 | -4.62611 |
| 882 | Nr3c2 | -0.70898 | 8.226006 | -5.9441 | 2.56E-05 | 0.000505 | 2.484034 |
| 883 | Rasd1 | -0.70903 | 9.718732 | -2.90697 | 0.010735 | 0.048116 | -3.57148 |
| 884 | Tmem28 | -0.71054 | 6.067352 | -7.62807 | 1.43E-06 | 5.99E-05 | 5.427725 |
| 885 | Grina | -0.71137 | 13.44456 | -8.38969 | 4.37E-07 | 2.51E-05 | 6.631819 |
| 886 | Fam69b | -0.71454 | 11.1634 | -2.7621 | 0.014405 | 0.060711 | -3.85487 |
| 887 | Pdlim5 | -0.71722 | 6.492906 | -5.20935 | 0.000102 | 0.001368 | 1.079365 |
| 888 | Irf5 | -0.7183 | 7.14469 | -3.19034 | 0.006007 | 0.030755 | -3.00641 |
| 889 | 2700081O15Rik | -0.71864 | 8.31207 | -8.07114 | 7.11E-07 | 3.59E-05 | 6.137506 |
| 890 | Gtf2h2 | -0.71925 | 8.720747 | -2.98209 | 0.009209 | 0.042891 | -3.42292 |
| 891 | Ranbp10 | -0.72021 | 10.78316 | -4.98544 | 0.000157 | 0.001903 | 0.638407 |
| 892 | Vps39 | -0.72123 | 9.905229 | -5.16587 | 0.000111 | 0.001456 | 0.994175 |
| 893 | Jun | -0.72178 | 13.97956 | -2.96779 | 0.009482 | 0.043854 | -3.45127 |
| 894 | Gfra1 | -0.72206 | 8.058103 | -4.99306 | 0.000155 | 0.00188 | 0.653512 |
| 895 | 3110070M22Rik | -0.72217 | 7.083607 | -5.39541 | 7.13E-05 | 0.001046 | 1.441425 |
| 896 | Adarb2 | -0.72472 | 5.335035 | -5.1103 | 0.000123 | 0.001577 | 0.884968 |
| 897 | Thrsp | -0.72577 | 12.01415 | -10.7544 | 1.69E-08 | 2.51E-06 | 9.919439 |
| 898 | Ntf3 | -0.72638 | 10.10119 | -2.50964 | 0.023886 | 0.089865 | -4.33676 |
| 899 | Atp9a | -0.72808 | 13.40085 | -2.68375 | 0.01687 | 0.068788 | -4.0062 |
| 900 | Chst15 | -0.72868 | 8.069321 | -5.65741 | 4.35E-05 | 0.000736 | 1.944107 |
| 901 | Rbm33 | -0.72887 | 9.799402 | -2.76069 | 0.014446 | 0.060816 | -3.85761 |
| 902 | C330011M18Rik | -0.72942 | 6.323874 | -7.77389 | 1.13E-06 | 5.07E-05 | 5.664217 |
| 903 | Slitrk3 | -0.73104 | 9.882666 | -9.89257 | 5.17E-08 | 5.81E-06 | 8.794608 |
| 904 | X99384 | -0.7314 | 7.79691 | -5.83284 | 3.14E-05 | 0.000581 | 2.275794 |
| 905 | Gm7251 | -0.73299 | 15.34654 | -15.6597 | 8.84E-11 | 5.31E-08 | 15.0789 |
| 906 | Iqca | -0.73328 | 6.8133 | -3.64341 | 0.002361 | 0.01486 | -2.08491 |
| 907 | Syt17 | -0.73421 | 9.373826 | -5.24085 | 9.58E-05 | 0.001311 | 1.140954 |
| 908 | Shisa6 | -0.73433 | 12.03766 | -3.24106 | 0.005412 | 0.028251 | -2.90411 |
| 909 | Cd163l1 | -0.73439 | 5.512766 | -3.45901 | 0.003453 | 0.019938 | -2.4617 |
| 910 | Dmxl2 | -0.73468 | 7.936073 | -6.15259 | 1.75E-05 | 0.000384 | 2.869778 |
| 911 | Slc27a3 | -0.73524 | 10.26492 | -3.10983 | 0.007089 | 0.034996 | -3.16817 |
| 912 | Glipr1 | -0.73781 | 6.506363 | -4.52167 | 0.000394 | 0.00377 | -0.29075 |
| 913 | Pop5 | -0.7392 | 9.934974 | -4.56191 | 0.000363 | 0.003555 | -0.2094 |
| 914 | Zfp651 | -0.73931 | 6.078098 | -3.49616 | 0.003198 | 0.018817 | -2.38591 |
| 915 | Chst9 | -0.73964 | 6.461123 | -3.17475 | 0.006203 | 0.031552 | -3.0378 |
| 916 | Gm5868 | -0.73984 | 5.468878 | -3.61196 | 0.002519 | 0.015641 | -2.14926 |
| 917 | C1ql2 | -0.74309 | 8.455311 | -2.93918 | 0.010052 | 0.045802 | -3.50789 |
| 918 | Mat2a | -0.74542 | 11.59957 | -2.50881 | 0.023926 | 0.089967 | -4.33831 |
| 919 | Hectd2 | -0.74825 | 10.32152 | -2.1738 | 0.045939 | 0.147781 | -4.94669 |
| 920 | Epha10 | -0.7497 | 6.352556 | -2.73572 | 0.015193 | 0.0632 | -3.90598 |
| 921 | 2900040C04Rik | -0.75042 | 10.78966 | -6.8554 | 5.12E-06 | 0.000155 | 4.125933 |
| 922 | Jph1 | -0.75118 | 6.374581 | -4.59965 | 0.000337 | 0.003355 | -0.13321 |
| 923 | Mc4r | -0.75201 | 8.313737 | -5.01765 | 0.000148 | 0.001815 | 0.702173 |
| 924 | Ndrg3 | -0.75324 | 11.80149 | -2.61295 | 0.019444 | 0.076637 | -4.14162 |
| 925 | Pdzd3 | -0.75342 | 6.227132 | -3.87572 | 0.001464 | 0.010265 | -1.6088 |
| 926 | Traf3ip3 | -0.75342 | 5.773882 | -7.22592 | 2.75E-06 | 9.82E-05 | 4.760466 |
| 927 | Ccdc86 | -0.75434 | 10.19156 | -2.30833 | 0.03546 | 0.121501 | -4.70729 |
| 928 | Pyroxd2 | -0.75514 | 6.283004 | -3.68426 | 0.00217 | 0.013911 | -2.00124 |
| 929 | Slc26a4 | -0.75563 | 12.25371 | -3.68424 | 0.00217 | 0.013911 | -2.00128 |
| 930 | Rnf32 | -0.75757 | 9.01604 | -6.61549 | 7.74E-06 | 0.00021 | 3.704858 |
| 931 | 3000002C10Rik | -0.75795 | 15.36057 | -12.9943 | 1.25E-09 | 3.39E-07 | 12.50912 |
| 932 | Tmem8 | -0.75865 | 7.943949 | -2.52318 | 0.023253 | 0.088011 | -4.31136 |
| 933 | Fam129c | -0.7598 | 5.867542 | -4.61609 | 0.000326 | 0.003279 | -0.10005 |
| 934 | Krt10 | -0.76344 | 7.779668 | -5.88747 | 2.84E-05 | 0.000543 | 2.378254 |
| 935 | Ccno | -0.76352 | 8.773204 | -4.3302 | 0.000579 | 0.005066 | -0.67939 |
| 936 | Gdf10 | -0.76448 | 8.700983 | -4.06212 | 0.000999 | 0.007713 | -1.22684 |
| 937 | Ndnl2 | -0.76778 | 11.14164 | -6.5735 | 8.32E-06 | 0.000221 | 3.630331 |
| 938 | Adamts1 | -0.77038 | 9.786318 | -3.42924 | 0.003672 | 0.020916 | -2.52234 |
| 939 | Gdf11 | -0.77042 | 5.418837 | -5.44734 | 6.46E-05 | 0.000972 | 1.541738 |
| 940 | Cdc40 | -0.77265 | 8.919496 | -14.3312 | 3.13E-10 | 1.26E-07 | 13.85904 |
| 941 | Cspp1 | -0.7729 | 8.462351 | -3.21539 | 0.005706 | 0.029518 | -2.95592 |
| 942 | Atp6v1c2 | -0.77444 | 7.934243 | -2.71582 | 0.015815 | 0.065357 | -3.94443 |
| 943 | Cby3 | -0.77507 | 5.268922 | -8.72826 | 2.64E-07 | 1.77E-05 | 7.142942 |
| 944 | Gstm6 | -0.77516 | 9.315677 | -6.60042 | 7.94E-06 | 0.000214 | 3.678143 |
| 945 | Nckap5 | -0.77625 | 6.84533 | -7.78207 | 1.12E-06 | 5.04E-05 | 5.677402 |
| 946 | Lrp2 | -0.77732 | 5.527339 | -2.84902 | 0.012078 | 0.052753 | -3.68536 |
| 947 | Alpk1 | -0.77777 | 6.726396 | -6.84009 | 5.25E-06 | 0.000158 | 4.099305 |
| 948 | Kctd13 | -0.7787 | 12.56283 | -2.96703 | 0.009497 | 0.043894 | -3.45277 |
| 949 | Wnt9b | -0.78049 | 5.330217 | -2.87697 | 0.011411 | 0.050478 | -3.63051 |
| 950 | Gm5423 | -0.78116 | 5.376777 | -4.18136 | 0.000783 | 0.006393 | -0.98296 |
| 951 | Zcwpw1 | -0.78413 | 5.660668 | -5.69701 | 4.04E-05 | 0.000699 | 2.019336 |
| 952 | Gp1bb | -0.7857 | 6.422539 | -3.20154 | 0.005871 | 0.030216 | -2.98385 |
| 953 | Lhx9 | -0.78916 | 7.511422 | -3.85107 | 0.00154 | 0.01066 | -1.65933 |
| 954 | Slc44a5 | -0.78937 | 8.722525 | -6.11848 | 1.87E-05 | 0.000399 | 2.80707 |
| 955 | Six4 | -0.79093 | 6.057522 | -4.53945 | 0.00038 | 0.003671 | -0.25478 |
| 956 | Tanc1 | -0.79306 | 8.007369 | -5.49306 | 5.93E-05 | 0.000913 | 1.629793 |
| 957 | Npnt | -0.79313 | 6.06074 | -4.10133 | 0.000922 | 0.007248 | -1.14659 |
| 958 | Cidea | -0.79595 | 11.70998 | -2.9023 | 0.010837 | 0.048467 | -3.58068 |
| 959 | 4932443I19Rik | -0.79733 | 6.943829 | -2.48387 | 0.025137 | 0.093405 | -4.38493 |
| 960 | Snca | -0.80369 | 15.73513 | -7.19567 | 2.89E-06 | 0.000102 | 4.709381 |
| 961 | Nhlh2 | -0.80789 | 5.794136 | -3.57317 | 0.002729 | 0.016681 | -2.2286 |
| 962 | Hadha | -0.80948 | 9.051136 | -10.906 | 1.40E-08 | 2.18E-06 | 10.10932 |
| 963 | Gpr115 | -0.81297 | 6.678841 | -2.64343 | 0.018293 | 0.073342 | -4.08349 |
| 964 | Ptk2b | -0.81494 | 13.02291 | -5.40106 | 7.06E-05 | 0.001035 | 1.45237 |
| 965 | Tcta | -0.8159 | 8.458536 | -2.44615 | 0.02708 | 0.098746 | -4.45508 |
| 966 | Dpf3 | -0.81698 | 7.166697 | -3.5148 | 0.003078 | 0.018265 | -2.34787 |
| 967 | D330050I16Rik | -0.82354 | 5.821887 | -5.52104 | 5.62E-05 | 0.00088 | 1.683544 |
| 968 | Prdm5 | -0.82395 | 9.60634 | -3.38305 | 0.004039 | 0.022507 | -2.61634 |
| 969 | 1700017B05Rik | -0.82544 | 8.314759 | -6.37146 | 1.19E-05 | 0.000288 | 3.268328 |
| 970 | Cdh9 | -0.82684 | 10.63874 | -4.22719 | 0.000714 | 0.005979 | -0.88937 |
| 971 | Ccbe1 | -0.82786 | 6.493424 | -3.56293 | 0.002787 | 0.01696 | -2.24955 |
| 972 | Galnt3 | -0.83001 | 7.59177 | -4.31297 | 0.0006 | 0.005186 | -0.71448 |
| 973 | Lpl | -0.8368 | 10.93009 | -3.72863 | 0.00198 | 0.012914 | -1.91033 |
| 974 | Tmprss9 | -0.83723 | 7.142209 | -3.11588 | 0.007001 | 0.034697 | -3.15605 |
| 975 | Kcng3 | -0.83726 | 5.067879 | -6.05182 | 2.10E-05 | 0.000439 | 2.684066 |
| 976 | LOC100039024 | -0.83858 | 6.352235 | -9.58276 | 7.86E-08 | 7.67E-06 | 8.370537 |
| 977 | Pkp2 | -0.83883 | 11.77588 | -3.61437 | 0.002506 | 0.015586 | -2.14433 |
| 978 | C2cd4c | -0.83904 | 7.194571 | -5.10662 | 0.000124 | 0.001584 | 0.877728 |
| 979 | Sebox | -0.83986 | 8.272664 | -3.18183 | 0.006114 | 0.031182 | -3.02354 |
| 980 | C4bp | -0.84114 | 6.117524 | -2.29577 | 0.036334 | 0.123901 | -4.72995 |
| 981 | Dnahc1 | -0.84179 | 5.533529 | -4.29097 | 0.000627 | 0.005366 | -0.75929 |
| 982 | Gins2 | -0.84248 | 6.551891 | -2.39665 | 0.029847 | 0.106389 | -4.54642 |
| 983 | Serpina3n | -0.8443 | 10.53398 | -4.78018 | 0.000235 | 0.002569 | 0.229591 |
| 984 | Gm3970 | -0.84841 | 5.194145 | -5.9222 | 2.67E-05 | 0.00052 | 2.443167 |
| 985 | Wbscr17 | -0.84975 | 8.879205 | -4.41822 | 0.000485 | 0.004402 | -0.50042 |
| 986 | Cenpe | -0.85016 | 8.84668 | -3.26453 | 0.005157 | 0.027245 | -2.85668 |
| 987 | Clec18a | -0.85079 | 6.900303 | -4.5772 | 0.000352 | 0.003473 | -0.17852 |
| 988 | Rreb1 | -0.85356 | 8.169249 | -2.63365 | 0.018654 | 0.074398 | -4.10215 |
| 989 | Zfp608 | -0.85497 | 10.10931 | -8.78447 | 2.43E-07 | 1.65E-05 | 7.2264 |
| 990 | Gm7120 | -0.85509 | 8.297494 | -8.91952 | 2.00E-07 | 1.42E-05 | 7.425342 |
| 991 | Iffo2 | -0.85547 | 8.245854 | -2.53581 | 0.022677 | 0.086377 | -4.28761 |
| 992 | Rhpn1 | -0.85695 | 6.818567 | -2.85273 | 0.011987 | 0.052465 | -3.67809 |
| 993 | Heatr7a | -0.85752 | 6.495869 | -3.45059 | 0.003514 | 0.020197 | -2.47885 |
| 994 | Fgf10 | -0.86353 | 9.235145 | -3.92558 | 0.001321 | 0.009544 | -1.50658 |
| 995 | Il1r1 | -0.86557 | 8.610991 | -2.97386 | 0.009365 | 0.043443 | -3.43924 |
| 996 | Wbscr25 | -0.86979 | 5.222306 | -4.75723 | 0.000246 | 0.002663 | 0.183615 |
| 997 | Homer3 | -0.87014 | 6.632491 | -3.98545 | 0.001169 | 0.008693 | -1.38388 |
| 998 | Dgkh | -0.87308 | 11.06184 | -2.88114 | 0.011314 | 0.050132 | -3.62231 |
| 999 | Bcat2 | -0.87359 | 7.767936 | -2.15293 | 0.047802 | 0.15246 | -4.98317 |
| 1000 | Parvg | -0.87875 | 8.080474 | -5.43931 | 6.56E-05 | 0.000984 | 1.526258 |
| 1001 | Rerg | -0.87992 | 7.106182 | -3.01039 | 0.008691 | 0.041012 | -3.3667 |
| 1002 | Mndal | -0.88085 | 6.532919 | -3.86389 | 0.0015 | 0.010432 | -1.63306 |
| 1003 | Wfikkn1 | -0.88138 | 5.298267 | -8.74095 | 2.59E-07 | 1.74E-05 | 7.161819 |
| 1004 | Tbxa2r | -0.88341 | 7.160706 | -6.80896 | 5.54E-06 | 0.000164 | 4.045058 |
| 1005 | Kcnip2 | -0.88731 | 9.558462 | -4.50509 | 0.000407 | 0.003866 | -0.3243 |
| 1006 | Slc9a4 | -0.89587 | 8.420408 | -2.84196 | 0.012253 | 0.053377 | -3.69919 |
| 1007 | Ehd1 | -0.89621 | 11.52687 | -2.64606 | 0.018196 | 0.073127 | -4.07845 |
| 1008 | Il16 | -0.89806 | 9.648031 | -2.86449 | 0.011704 | 0.051531 | -3.65502 |
| 1009 | Gjb3 | -0.89964 | 5.650101 | -4.96825 | 0.000163 | 0.001959 | 0.604333 |
| 1010 | Nrl | -0.89996 | 8.161925 | -3.54473 | 0.002893 | 0.017442 | -2.28674 |
| 1011 | Mapkbp1 | -0.90685 | 5.560345 | -6.50269 | 9.42E-06 | 0.000242 | 3.504109 |
| 1012 | Drd5 | -0.91034 | 7.20297 | -3.28896 | 0.004904 | 0.026205 | -2.80724 |
| 1013 | 6230427J02Rik | -0.91427 | 6.369743 | -5.92922 | 2.63E-05 | 0.000516 | 2.456269 |
| 1014 | Zbtb20 | -0.91544 | 12.47757 | -4.99633 | 0.000154 | 0.00187 | 0.659972 |
| 1015 | Epb4.1l4a | -0.91663 | 7.180681 | -5.58166 | 5.01E-05 | 0.000811 | 1.799656 |
| 1016 | Zcchc13 | -0.92325 | 5.173889 | -7.59216 | 1.51E-06 | 6.24E-05 | 5.36903 |
| 1017 | C2cd4a | -0.92469 | 6.042087 | -2.59007 | 0.020353 | 0.079435 | -4.18509 |
| 1018 | Hist2h2be | -0.92733 | 7.988421 | -2.27317 | 0.037958 | 0.128066 | -4.77054 |
| 1019 | Cldn26 | -0.92867 | 8.713678 | -3.23935 | 0.005431 | 0.028324 | -2.90756 |
| 1020 | Mdga1 | -0.9401 | 8.782214 | -5.86467 | 2.96E-05 | 0.000557 | 2.335533 |
| 1021 | Klrg1 | -0.9441 | 5.356795 | -9.11771 | 1.51E-07 | 1.16E-05 | 7.713259 |
| 1022 | Chrna1 | -0.94598 | 5.951392 | -4.94308 | 0.000171 | 0.002038 | 0.554376 |
| 1023 | 4930579J09Rik | -0.94645 | 6.963337 | -2.37258 | 0.031288 | 0.110293 | -4.59055 |
| 1024 | Crlf1 | -0.94832 | 9.490131 | -3.46805 | 0.003389 | 0.019678 | -2.44325 |
| 1025 | Sipa1l3 | -0.95136 | 12.70485 | -2.34799 | 0.032827 | 0.114491 | -4.6354 |
| 1026 | Klk8 | -0.95204 | 10.06191 | -2.9567 | 0.009699 | 0.044581 | -3.47324 |
| 1027 | Popdc3 | -0.95394 | 8.041632 | -3.63727 | 0.002391 | 0.015015 | -2.09747 |
| 1028 | Orc2 | -0.9541 | 7.2839 | -2.92818 | 0.010281 | 0.046592 | -3.52964 |
| 1029 | Robo3 | -0.95489 | 8.471071 | -2.59686 | 0.02008 | 0.078617 | -4.17222 |
| 1030 | Umodl1 | -0.95572 | 6.168468 | -3.82661 | 0.001619 | 0.011077 | -1.70949 |
| 1031 | Adra1d | -0.96503 | 8.984922 | -4.64459 | 0.000308 | 0.003162 | -0.04265 |
| 1032 | Smoc2 | -0.96564 | 12.54049 | -3.3452 | 0.004367 | 0.023929 | -2.69323 |
| 1033 | St18 | -0.97077 | 8.316155 | -2.71272 | 0.015914 | 0.065644 | -3.95042 |
| 1034 | Pcdhb9 | -0.97788 | 7.105268 | -10.0364 | 4.27E-08 | 5.11E-06 | 8.987791 |
| 1035 | Vwa3a | -0.98267 | 7.758969 | -3.31902 | 0.004609 | 0.024945 | -2.74633 |
| 1036 | Tspan18 | -1.01323 | 9.135506 | -4.82233 | 0.000217 | 0.002414 | 0.313871 |
| 1037 | Fst | -1.01331 | 8.722084 | -3.24167 | 0.005405 | 0.028243 | -2.90289 |
| 1038 | Ccnjl | -1.03072 | 10.1811 | -5.59113 | 4.93E-05 | 0.000802 | 1.817763 |
| 1039 | Tjp3 | -1.04391 | 7.964018 | -3.8618 | 0.001506 | 0.01047 | -1.63733 |
| 1040 | Avp | -1.04761 | 5.91102 | -2.93133 | 0.010215 | 0.046417 | -3.52341 |
| 1041 | Tyrp1 | -1.05198 | 5.659817 | -4.11446 | 0.000898 | 0.0071 | -1.11973 |
| 1042 | 1110002N22Rik | -1.05781 | 7.785221 | -2.78674 | 0.013704 | 0.058329 | -3.80699 |
| 1043 | Creb3l3 | -1.05843 | 7.245479 | -3.30656 | 0.004729 | 0.025444 | -2.77159 |
| 1044 | D330025C20Rik | -1.06065 | 6.09291 | -5.04036 | 0.000141 | 0.00175 | 0.747068 |
| 1045 | Ryr1 | -1.06767 | 7.363964 | -4.21862 | 0.000726 | 0.00605 | -0.90687 |
| 1046 | Cabp7 | -1.071 | 7.319771 | -5.22883 | 9.81E-05 | 0.001331 | 1.117467 |
| 1047 | Ghsr | -1.07266 | 5.832386 | -4.45861 | 0.000447 | 0.00414 | -0.41847 |
| 1048 | Pcdh20 | -1.0851 | 12.76891 | -4.77086 | 0.00024 | 0.002602 | 0.210919 |
| 1049 | Olfml2b | -1.0878 | 9.47393 | -4.0439 | 0.001037 | 0.007913 | -1.26415 |
| 1050 | D4Bwg0951e | -1.09609 | 11.49078 | -2.23661 | 0.040731 | 0.135192 | -4.83581 |
| 1051 | C2cd4b | -1.09779 | 8.389347 | -2.25266 | 0.039491 | 0.132071 | -4.80722 |
| 1052 | Prox1 | -1.10375 | 8.164313 | -4.46737 | 0.000439 | 0.004085 | -0.40072 |
| 1053 | Gm606 | -1.11099 | 6.246002 | -3.39235 | 0.003962 | 0.02218 | -2.59742 |
| 1054 | Tmem171 | -1.11822 | 7.258166 | -4.03103 | 0.001065 | 0.008075 | -1.2905 |
| 1055 | 4933427G17Rik | -1.12397 | 7.504265 | -4.41926 | 0.000484 | 0.004398 | -0.49831 |
| 1056 | Zap70 | -1.14847 | 9.071802 | -3.90637 | 0.001374 | 0.009837 | -1.54597 |
| 1057 | Frzb | -1.1501 | 9.649089 | -4.21705 | 0.000729 | 0.006067 | -0.91008 |
| 1058 | 9030224M15Rik | -1.15278 | 7.01337 | -4.57804 | 0.000352 | 0.00347 | -0.17682 |
| 1059 | Dsp | -1.1753 | 9.452206 | -3.00377 | 0.00881 | 0.041407 | -3.37986 |
| 1060 | Klk1b27 | -1.18826 | 5.696231 | -5.25702 | 9.29E-05 | 0.00128 | 1.172506 |
| 1061 | Lrrc10b | -1.24182 | 8.961616 | -4.27076 | 0.000653 | 0.005549 | -0.8005 |
| 1062 | 5830408C22Rik | -1.27016 | 7.012622 | -3.17394 | 0.006214 | 0.031576 | -3.03942 |
| 1063 | Gm52 | -1.28572 | 6.021975 | -5.27676 | 8.95E-05 | 0.001245 | 1.211009 |
| 1064 | Il20rb | -1.30533 | 6.687642 | -3.6444 | 0.002356 | 0.014839 | -2.08288 |
| 1065 | Fcgbp | -1.3181 | 6.380846 | -4.06491 | 0.000993 | 0.007687 | -1.22112 |
| 1066 | Olfr550 | -1.34066 | 5.85029 | -6.00898 | 2.28E-05 | 0.000462 | 2.604714 |
| 1067 | Loxl1 | -1.36876 | 8.015667 | -5.09575 | 0.000127 | 0.00161 | 0.856328 |
| 1068 | Cldn22 | -1.38678 | 9.680569 | -2.92528 | 0.010342 | 0.046781 | -3.53536 |
| 1069 | Slco2a1 | -1.42659 | 8.779044 | -4.34853 | 0.000558 | 0.004919 | -0.64208 |
| 1070 | Slc26a10 | -1.45812 | 8.211031 | -2.74191 | 0.015004 | 0.062594 | -3.89401 |
| 1071 | Rasl11a | -1.48893 | 8.167993 | -5.91575 | 2.70E-05 | 0.000524 | 2.431134 |

**Table S2. The statistical metrics for key differentially expressed genes (DEGs with Fold Change> 1.2 ) in ACC**

| **NO** | **Gene** | **logFC** | **AveExpr** | **t** | **P.Value** | **adj.P.Val** | **B** |
| --- | --- | --- | --- | --- | --- | --- | --- |
| 1 | Ppm1g | 0.28050887 | 9.9355625 | 6.50057 | 9.53E-06 | 0.04260117 | 3.462284849 |
| 2 | Mab21l2 | 0.28137876 | 5.3150601 | 6.477407 | 9.92E-06 | 0.04260117 | 3.428592046 |
| 3 | Igfbp4 | 0.53024198 | 8.3349793 | 6.388724 | 1.16E-05 | 0.04260117 | 3.298736171 |
| 4 | Gm5316 | 0.28841053 | 9.7261361 | 5.988345 | 2.38E-05 | 0.05241625 | 2.695504904 |
| 5 | Foxi1 | 0.53649127 | 5.2686647 | 5.843529 | 3.10E-05 | 0.05323993 | 2.470502767 |
| 6 | Rmnd5b | 0.27472669 | 10.506479 | 5.809856 | 3.30E-05 | 0.05323993 | 2.417670718 |
| 7 | Mpp2 | 0.31715231 | 8.1218886 | 5.43008 | 6.71E-05 | 0.07195837 | 1.808562867 |
| 8 | Slc35a4 | 0.26741408 | 9.1644727 | 5.411946 | 6.95E-05 | 0.07195837 | 1.778879563 |
| 9 | Rrp9 | 0.33295727 | 9.7112517 | 5.35015 | 7.82E-05 | 0.07489819 | 1.677325188 |
| 10 | Ptcd1 | 0.31618052 | 7.4266137 | 5.275547 | 9.01E-05 | 0.08064384 | 1.553903334 |
| 11 | Cd37 | 0.31591787 | 6.3455458 | 5.267881 | 9.15E-05 | 0.08064384 | 1.541170192 |
| 12 | Casp14 | 0.74996863 | 6.5257186 | 5.183322 | 0.00010763 | 0.08179298 | 1.40010623 |
| 13 | Wfdc13 | 0.65106439 | 10.191444 | 5.133897 | 0.00011841 | 0.08598227 | 1.317134642 |
| 14 | Sun5 | 0.36263345 | 6.6684753 | 5.122938 | 0.00012094 | 0.08598227 | 1.298687096 |
| 15 | Rtn4rl2 | 0.41042114 | 6.5106039 | 4.90595 | 0.00018459 | 0.09470365 | 0.929682508 |
| 16 | Meis3 | 0.281044 | 8.8406264 | 4.905433 | 0.00018478 | 0.09470365 | 0.928794788 |
| 17 | Fto | 0.28280164 | 10.96556 | 4.828596 | 0.0002149 | 0.10498851 | 0.796470723 |
| 18 | Srm | 0.30362947 | 12.115039 | 4.648453 | 0.00030703 | 0.12218204 | 0.483041562 |
| 19 | Prss37 | 0.33522616 | 7.8588312 | 4.625092 | 0.00032164 | 0.12218204 | 0.442081393 |
| 20 | Lrfn1 | 0.3165703 | 7.8426841 | 4.558897 | 0.00036707 | 0.12373795 | 0.325640652 |
| 21 | Rbm3 | 0.40638724 | 9.3891698 | 4.550043 | 0.00037363 | 0.12373795 | 0.310026038 |
| 22 | Kbtbd5 | 0.54497363 | 8.2105705 | 4.439881 | 0.000466 | 0.12373795 | 0.114945974 |
| 23 | Slc6a16 | 0.31602314 | 4.7749005 | 4.416646 | 0.0004883 | 0.12555361 | 0.07362035 |
| 24 | Chrna2 | 0.37583704 | 7.9165316 | 4.289374 | 0.00063134 | 0.13914103 | -0.15378792 |
| 25 | Actl6b | 0.29343767 | 9.2782274 | 4.244459 | 0.00069148 | 0.14295543 | -0.23444247 |
| 26 | Cdc23 | 0.54995426 | 5.7807579 | 4.235962 | 0.0007035 | 0.14295543 | -0.2497228 |
| 27 | Arl4c | 0.48678893 | 12.228709 | 4.215377 | 0.00073351 | 0.14295543 | -0.28676812 |
| 28 | Fibp | 0.30848276 | 11.097951 | 4.199408 | 0.00075769 | 0.14295543 | -0.31553383 |
| 29 | H1fx | 0.47123075 | 9.0990457 | 4.185765 | 0.00077899 | 0.14295543 | -0.34012999 |
| 30 | Cndp2 | 0.27083922 | 8.8525789 | 4.151051 | 0.00083596 | 0.14295543 | -0.40278528 |
| 31 | Tut1 | 0.29923672 | 8.1043394 | 4.122662 | 0.0008857 | 0.14295543 | -0.45410306 |
| 32 | Sox13 | 0.31647431 | 8.2582509 | 4.117591 | 0.0008949 | 0.14295543 | -0.46327841 |
| 33 | Myo3b | 0.38085559 | 7.4423775 | 4.117346 | 0.00089535 | 0.14295543 | -0.46372168 |
| 34 | March9 | 0.26758012 | 12.208558 | 4.103487 | 0.000921 | 0.14295543 | -0.48880505 |
| 35 | Bzw2 | 0.29054242 | 11.221205 | 4.093421 | 0.00094009 | 0.14387934 | -0.50703181 |
| 36 | Gm3625 | 0.35807395 | 6.9952568 | 4.020755 | 0.00109041 | 0.15306769 | -0.63885703 |
| 37 | Fam194a | 0.31743884 | 7.9299665 | 3.970136 | 0.00120935 | 0.15466473 | -0.73091504 |
| 38 | Tm4sf5 | 0.41487194 | 8.4498788 | 3.937135 | 0.0012939 | 0.15466473 | -0.7910249 |
| 39 | G6pd2 | 0.32949683 | 4.8641502 | 3.931056 | 0.00131011 | 0.15466473 | -0.8021044 |
| 40 | Nanos3 | 0.31400875 | 10.180957 | 3.926219 | 0.00132316 | 0.15466473 | -0.81092308 |
| 41 | Fahd2a | 0.29842594 | 11.236591 | 3.925929 | 0.00132395 | 0.15466473 | -0.81145178 |
| 42 | Grwd1 | 0.2678621 | 8.8708135 | 3.921872 | 0.001335 | 0.15466473 | -0.81884811 |
| 43 | Nipsnap1 | 0.28371891 | 11.421248 | 3.896386 | 0.0014066 | 0.15736106 | -0.86533983 |
| 44 | Prkcc | 0.43585254 | 11.090324 | 3.866371 | 0.00149593 | 0.1608234 | -0.92013784 |
| 45 | Gm13178 | 0.5174146 | 5.7725106 | 3.833684 | 0.00159975 | 0.16604867 | -0.97987081 |
| 46 | Rgs14 | 0.42343569 | 12.464629 | 3.817 | 0.00165552 | 0.16660325 | -1.01037955 |
| 47 | Gm3952 | 0.47389277 | 5.7484371 | 3.762757 | 0.00185078 | 0.16696695 | -1.10965493 |
| 48 | Acr | 0.43981505 | 5.9515357 | 3.723376 | 0.00200695 | 0.17126572 | -1.18180079 |
| 49 | Man2b2 | 0.30059312 | 7.9483008 | 3.722368 | 0.00201112 | 0.17126572 | -1.18364856 |
| 50 | Rhbdl1 | 0.2824853 | 11.116666 | 3.717858 | 0.00202987 | 0.17126572 | -1.19191452 |
| 51 | Taok2 | 0.34534194 | 7.3808429 | 3.674805 | 0.00221798 | 0.17457858 | -1.27085223 |
| 52 | Ccl17 | 0.50152489 | 8.8793488 | 3.65762 | 0.00229788 | 0.17554603 | -1.30237502 |
| 53 | Gm10035 | 0.4067569 | 8.7663663 | 3.630953 | 0.00242763 | 0.17834144 | -1.35130274 |
| 54 | Cdc42bpb | 0.33429108 | 9.0247352 | 3.609317 | 0.00253829 | 0.18214442 | -1.39101075 |
| 55 | Smyd5 | 0.32684344 | 8.2183576 | 3.597841 | 0.00259903 | 0.18242043 | -1.41207414 |
| 56 | Rad23a | 0.27599611 | 6.766929 | 3.589334 | 0.002645 | 0.18331149 | -1.4276899 |
| 57 | H2-DMa | 0.31522991 | 9.1146805 | 3.582134 | 0.00268454 | 0.18360632 | -1.44090774 |
| 58 | Dgcr14 | 0.26562215 | 10.43095 | 3.559572 | 0.00281232 | 0.18654808 | -1.48232755 |
| 59 | Grin1 | 0.31058001 | 9.5846869 | 3.537225 | 0.0029449 | 0.18894897 | -1.5233568 |
| 60 | Tgfbrap1 | 0.26553296 | 7.9053359 | 3.513173 | 0.0030946 | 0.19224608 | -1.56751647 |
| 61 | Armc7 | 0.27970701 | 8.135597 | 3.501729 | 0.00316848 | 0.19224608 | -1.5885278 |
| 62 | Lta | 0.51361252 | 5.6268061 | 3.497234 | 0.00319798 | 0.19224608 | -1.59678081 |
| 63 | Tgif2lx1 | 0.39264529 | 6.7805584 | 3.497138 | 0.00319861 | 0.19224608 | -1.59695649 |
| 64 | Trim62 | 0.29876567 | 8.9144134 | 3.490619 | 0.00324189 | 0.19224608 | -1.60892536 |
| 65 | Tspan4 | 0.46904248 | 9.6036105 | 3.485989 | 0.00327299 | 0.19224608 | -1.617426 |
| 66 | Mthfd1l | 0.3942383 | 9.1651773 | 3.48576 | 0.00327454 | 0.19224608 | -1.61784757 |
| 67 | Jsrp1 | 0.40623101 | 10.1723 | 3.482583 | 0.00329605 | 0.19224608 | -1.62367979 |
| 68 | Tbl3 | 0.32468993 | 8.5697806 | 3.480314 | 0.00331151 | 0.19224608 | -1.62784546 |
| 69 | Def6 | 0.31796955 | 10.826243 | 3.469068 | 0.00338919 | 0.19250065 | -1.64849192 |
| 70 | Rdh12 | 0.31870674 | 13.27642 | 3.46178 | 0.00344049 | 0.1931321 | -1.66186916 |
| 71 | Fam132a | 0.31844071 | 10.952416 | 3.449644 | 0.00352767 | 0.1948527 | -1.68414824 |
| 72 | Kcnh3 | 0.2668454 | 13.521004 | 3.442761 | 0.00357808 | 0.19519144 | -1.69678226 |
| 73 | Parp1 | 0.30942996 | 10.173408 | 3.414493 | 0.00379279 | 0.19608199 | -1.74866107 |
| 74 | Uap1l1 | 0.31827251 | 10.456442 | 3.413372 | 0.00380156 | 0.19608199 | -1.75071704 |
| 75 | Tas2r109 | 0.38975813 | 7.9048948 | 3.410298 | 0.00382573 | 0.19608199 | -1.75635787 |
| 76 | Kcnab2 | 0.40446405 | 8.0770971 | 3.399942 | 0.00390828 | 0.19677266 | -1.77536019 |
| 77 | Entpd6 | 0.30413095 | 9.8647263 | 3.395206 | 0.00394662 | 0.1976809 | -1.78404897 |
| 78 | Glt25d1 | 0.29376431 | 10.139316 | 3.369693 | 0.0041597 | 0.20212283 | -1.8308461 |
| 79 | Fmnl1 | 0.26595219 | 8.1613877 | 3.367069 | 0.00418226 | 0.20212283 | -1.83565812 |
| 80 | Ucp2 | 0.35661877 | 8.1496285 | 3.358453 | 0.00425718 | 0.20308225 | -1.85145729 |
| 81 | Mchr1 | 0.42962861 | 6.8487892 | 3.34629 | 0.00436523 | 0.20512874 | -1.87375816 |
| 82 | Pdzd9 | 0.330396 | 4.9055242 | 3.31635 | 0.00464297 | 0.20836841 | -1.92862934 |
| 83 | Vstm2l | 0.27833052 | 14.080549 | 3.28866 | 0.00491545 | 0.20994495 | -1.97934569 |
| 84 | Syngap1 | 0.37475057 | 5.7631178 | 3.284577 | 0.00495695 | 0.21130796 | -1.98682122 |
| 85 | Baiap2l2 | 0.50180738 | 10.325896 | 3.263541 | 0.00517634 | 0.21484262 | -2.02532407 |
| 86 | Morn1 | 0.30625712 | 7.8373552 | 3.256869 | 0.00524792 | 0.21591557 | -2.03753053 |
| 87 | Gm757 | 0.38502942 | 7.1872881 | 3.239741 | 0.0054362 | 0.21778041 | -2.06885835 |
| 88 | Fermt3 | 0.52590986 | 5.2008175 | 3.236902 | 0.00546804 | 0.21778041 | -2.07404943 |
| 89 | Psmf1 | 0.36408407 | 8.4374052 | 3.234338 | 0.00549697 | 0.21778041 | -2.07873776 |
| 90 | 2300005B03Rik | 0.28928583 | 8.8757735 | 3.216437 | 0.00570317 | 0.22090006 | -2.11145711 |
| 91 | Ubl7 | 0.27028024 | 11.369718 | 3.207227 | 0.00581221 | 0.22264953 | -2.12828216 |
| 92 | Rasal1 | 0.36714227 | 11.114313 | 3.193607 | 0.00597729 | 0.22403663 | -2.15315724 |
| 93 | Gm129 | 0.95824295 | 7.1357483 | 3.188487 | 0.00604053 | 0.22530678 | -2.16250369 |
| 94 | Sez6 | 0.34582084 | 10.446207 | 3.1751 | 0.00620905 | 0.22655824 | -2.18693647 |
| 95 | Krtap10-10 | 0.31233744 | 9.4153435 | 3.160616 | 0.00639661 | 0.2281146 | -2.21335705 |
| 96 | Gm438 | 0.42887271 | 5.4101552 | 3.15418 | 0.00648172 | 0.22856173 | -2.2250906 |
| 97 | 3830431G21Rik | 0.41888174 | 6.6715426 | 3.154179 | 0.00648174 | 0.22856173 | -2.22509278 |
| 98 | Ak8 | 0.40619233 | 7.1631188 | 3.143762 | 0.0066219 | 0.23176308 | -2.24408004 |
| 99 | Sbno2 | 0.31904491 | 6.5449636 | 3.127777 | 0.00684283 | 0.23422575 | -2.27320021 |
| 100 | Prkar2a | 0.29800195 | 8.1813999 | 3.126856 | 0.00685578 | 0.23422575 | -2.27487823 |
| 101 | Ephx1 | 0.28555141 | 10.36683 | 3.107793 | 0.00712928 | 0.23561096 | -2.30957728 |
| 102 | Ddn | 0.37573059 | 12.970874 | 3.106084 | 0.00715432 | 0.23568614 | -2.31268654 |
| 103 | Cyp2d22 | 0.43179511 | 5.67239 | 3.090054 | 0.00739344 | 0.2392718 | -2.34183998 |
| 104 | 4732415M23Rik | 0.30694806 | 6.0729175 | 3.086864 | 0.00744197 | 0.24013685 | -2.3476394 |
| 105 | L3mbtl2 | 0.28810612 | 8.2952033 | 3.080753 | 0.00753578 | 0.2417484 | -2.35874539 |
| 106 | Egln1 | 0.33065845 | 5.8855225 | 3.070628 | 0.0076938 | 0.2446341 | -2.37713875 |
| 107 | Acsf3 | 0.29229375 | 10.676157 | 3.062224 | 0.00782743 | 0.24527605 | -2.39239837 |
| 108 | Parvg | 0.37558445 | 8.0084148 | 3.057205 | 0.00790832 | 0.24679255 | -2.40150895 |
| 109 | Prr12 | 0.26522112 | 6.8962559 | 3.052734 | 0.00798108 | 0.24679255 | -2.4096229 |
| 110 | Hist1h3i | 0.28691799 | 4.7782031 | 3.043252 | 0.00813756 | 0.24891594 | -2.42682383 |
| 111 | Tcea2 | 0.28587446 | 8.6798788 | 3.02866 | 0.00838429 | 0.25006696 | -2.45327783 |
| 112 | Nt5dc2 | 0.33036495 | 5.2416637 | 3.026302 | 0.00842485 | 0.25006696 | -2.45755058 |
| 113 | Kank4 | 0.46638731 | 5.2702764 | 3.008496 | 0.00873735 | 0.25128139 | -2.4897947 |
| 114 | Slc25a45 | 0.30381369 | 6.2349331 | 3.007455 | 0.00875597 | 0.25128139 | -2.49167878 |
| 115 | Slc28a1 | 0.29709279 | 8.4545888 | 2.991174 | 0.0090523 | 0.25414483 | -2.52113213 |
| 116 | Gm436 | 0.3252386 | 5.0271941 | 2.962618 | 0.00959597 | 0.2605541 | -2.57271826 |
| 117 | Slc1a7 | 0.52570866 | 5.1794439 | 2.942086 | 0.0100065 | 0.26259944 | -2.60974845 |
| 118 | Glis1 | 0.27287544 | 9.7565319 | 2.941544 | 0.01001758 | 0.26259944 | -2.61072579 |
| 119 | Spata24 | 0.56505074 | 5.215899 | 2.934976 | 0.01015265 | 0.26504328 | -2.62256077 |
| 120 | Map3k15 | 0.28146751 | 5.1519949 | 2.920344 | 0.01045998 | 0.26619779 | -2.64890494 |
| 121 | Gm13304 | 0.34678939 | 12.748721 | 2.903138 | 0.01083303 | 0.2678406 | -2.67984881 |
| 122 | Gm9962 | 0.28513569 | 6.4714192 | 2.872991 | 0.01151823 | 0.27372392 | -2.73396947 |
| 123 | Apex1 | 0.38554795 | 11.597197 | 2.871417 | 0.01155514 | 0.27394214 | -2.73679154 |
| 124 | Mettl2 | 0.30315844 | 7.4250988 | 2.85575 | 0.01192892 | 0.27654471 | -2.76486421 |
| 125 | Gm16516 | 0.28365831 | 6.9003624 | 2.849134 | 0.01209027 | 0.27654471 | -2.77670743 |
| 126 | Gm6209 | 0.31562399 | 7.6204569 | 2.848715 | 0.01210055 | 0.27654471 | -2.77745707 |
| 127 | Rhoh | 0.31680268 | 8.7372855 | 2.844646 | 0.01220092 | 0.27654471 | -2.78473766 |
| 128 | Dbp | 0.64909808 | 14.205666 | 2.842429 | 0.01225596 | 0.27654471 | -2.78870419 |
| 129 | Rbp3 | 0.39568194 | 11.867552 | 2.835748 | 0.01242326 | 0.27745007 | -2.80065133 |
| 130 | 2200002K05Rik | 0.26525907 | 8.3282425 | 2.773153 | 0.01410248 | 0.29092587 | -2.91224138 |
| 131 | Cant1 | 0.2678025 | 6.1699132 | 2.772391 | 0.01412422 | 0.29092587 | -2.91359544 |
| 132 | Cd79a | 0.32653648 | 5.0595367 | 2.750888 | 0.01475133 | 0.29581845 | -2.95177738 |
| 133 | Nfkbib | 0.30872332 | 8.7647993 | 2.743956 | 0.01495918 | 0.29755 | -2.9640692 |
| 134 | Gkn1 | 0.31205667 | 13.275848 | 2.737298 | 0.01516148 | 0.2989857 | -2.97586665 |
| 135 | Mrpl1 | 0.32193238 | 7.7262646 | 2.730603 | 0.01536759 | 0.30034048 | -2.98772232 |
| 136 | Tmem210 | 0.38424252 | 5.3695973 | 2.72787 | 0.01545249 | 0.30034048 | -2.99255909 |
| 137 | Chrnb4 | 0.48821459 | 5.2630278 | 2.712121 | 0.01595072 | 0.30557746 | -3.02040813 |
| 138 | 9130213A22Rik | 0.36277783 | 7.6617955 | 2.706698 | 0.01612581 | 0.30676113 | -3.02998536 |
| 139 | Mri1 | 0.51386372 | 8.7618691 | 2.698962 | 0.01637884 | 0.30841557 | -3.04363955 |
| 140 | Tspyl2 | 0.27305432 | 11.889841 | 2.696172 | 0.01647103 | 0.30841557 | -3.04856118 |
| 141 | Gm6985 | 0.36605946 | 5.4571097 | 2.688632 | 0.01672273 | 0.31049049 | -3.06185603 |
| 142 | Lamb3 | 0.3476761 | 6.3427206 | 2.68508 | 0.01684254 | 0.31117635 | -3.06811336 |
| 143 | Gstm7 | 0.39640032 | 7.6506669 | 2.683361 | 0.01690086 | 0.31117635 | -3.07114253 |
| 144 | Tra2b | 0.3405412 | 5.1827119 | 2.652423 | 0.01798387 | 0.3177612 | -3.1255384 |
| 145 | Tbrg4 | 0.28645362 | 6.4037092 | 2.651082 | 0.0180323 | 0.3177612 | -3.12789205 |
| 146 | Ggn | 0.28248079 | 5.4898351 | 2.650644 | 0.01804811 | 0.3177612 | -3.12865928 |
| 147 | Wdr6 | 0.44397584 | 10.938526 | 2.643205 | 0.01831927 | 0.31779396 | -3.14170785 |
| 148 | Pkhd1 | 0.39842335 | 6.2211 | 2.630933 | 0.01877514 | 0.32276548 | -3.16320734 |
| 149 | Map2k3 | 0.33312191 | 6.9908347 | 2.625806 | 0.01896884 | 0.32403682 | -3.17218137 |
| 150 | Cebpa | 0.37918483 | 8.6216934 | 2.61299 | 0.01946141 | 0.32792739 | -3.1945868 |
| 151 | Armcx5 | 0.2663967 | 10.358758 | 2.611174 | 0.01953217 | 0.3286026 | -3.19775763 |
| 152 | Krt9 | 0.53932839 | 9.0215886 | 2.610467 | 0.0195598 | 0.3287445 | -3.19899259 |
| 153 | Nr1d1 | 0.59491394 | 8.9208436 | 2.603889 | 0.01981862 | 0.32999666 | -3.2104754 |
| 154 | Rrp8 | 0.34594084 | 7.1702956 | 2.601527 | 0.01991236 | 0.33061713 | -3.21459665 |
| 155 | Gm1332 | 0.29936105 | 5.69833 | 2.569289 | 0.02123497 | 0.34000384 | -3.27071519 |
| 156 | Gm3448 | 0.29204035 | 7.5966277 | 2.549642 | 0.02208186 | 0.34471973 | -3.30479957 |
| 157 | Ptk2b | 0.30361903 | 12.86154 | 2.546478 | 0.02222124 | 0.34516247 | -3.31028081 |
| 158 | Pipox | 0.5173591 | 6.8367054 | 2.529907 | 0.02296502 | 0.3514702 | -3.33894607 |
| 159 | 4933400C05Rik | 0.30534763 | 7.4915759 | 2.521653 | 0.02334432 | 0.35362788 | -3.35319994 |
| 160 | Cdhr1 | 0.39918224 | 8.2914676 | 2.513771 | 0.02371209 | 0.35499146 | -3.36679537 |
| 161 | Elane | 0.32339151 | 9.0426366 | 2.51347 | 0.02372623 | 0.35499146 | -3.3673139 |
| 162 | Ppp1r1b | 0.28513231 | 12.019779 | 2.510071 | 0.02388661 | 0.35699125 | -3.37317155 |
| 163 | Ppp1r3b | 0.29498385 | 10.796595 | 2.509591 | 0.02390937 | 0.35699125 | -3.37399966 |
| 164 | Cdh22 | 0.26702385 | 11.998521 | 2.496741 | 0.02452564 | 0.36006666 | -3.39611706 |
| 165 | Cyp4f13 | 0.29576297 | 8.0915576 | 2.486416 | 0.02503181 | 0.36320327 | -3.41386061 |
| 166 | LOC628110 | 0.27380462 | 4.9714705 | 2.481503 | 0.02527613 | 0.36431479 | -3.42229354 |
| 167 | Acy1 | 0.28682171 | 7.2121138 | 2.474697 | 0.0256183 | 0.36486915 | -3.43396432 |
| 168 | Tbc1d10c | 0.4266004 | 5.2544491 | 2.469898 | 0.02586227 | 0.36486915 | -3.44218811 |
| 169 | Zdhhc4 | 0.30334835 | 8.0177415 | 2.467973 | 0.02596072 | 0.36486915 | -3.4454841 |
| 170 | Pion | 0.28594208 | 6.6968163 | 2.464375 | 0.0261457 | 0.36493039 | -3.45164264 |
| 171 | Slc25a37 | 0.30513066 | 7.7333008 | 2.452565 | 0.02676181 | 0.36830498 | -3.47183616 |
| 172 | 1700001L05Rik | 0.36680999 | 8.8619333 | 2.44945 | 0.02692661 | 0.36930321 | -3.47715651 |
| 173 | Rbmx | 0.30693913 | 10.147037 | 2.442256 | 0.02731083 | 0.36971952 | -3.48943158 |
| 174 | Zfp57 | 0.26366372 | 6.9601893 | 2.432008 | 0.02786725 | 0.37299049 | -3.50689661 |
| 175 | Plbd2 | 0.27688481 | 11.129419 | 2.431467 | 0.0278969 | 0.37307022 | -3.50781726 |
| 176 | Sycp3 | 0.40226626 | 5.588613 | 2.428678 | 0.02805029 | 0.3740544 | -3.51256376 |
| 177 | 5430411C19Rik | 0.27102283 | 5.666888 | 2.421878 | 0.02842774 | 0.37413938 | -3.5241303 |
| 178 | Itgb7 | 0.29725182 | 6.0506423 | 2.417429 | 0.02867728 | 0.37413938 | -3.53169095 |
| 179 | Cirbp | 0.48182373 | 12.508729 | 2.408134 | 0.02920531 | 0.37450865 | -3.54746785 |
| 180 | Gm5868 | 0.45695723 | 5.2413946 | 2.38405 | 0.03061663 | 0.37929168 | -3.58823459 |
| 181 | Nfatc2 | 0.2801887 | 7.1613381 | 2.382154 | 0.03073044 | 0.37944472 | -3.59143713 |
| 182 | Osgin1 | 0.3904405 | 5.8346415 | 2.37495 | 0.03116646 | 0.37946848 | -3.60359441 |
| 183 | Notum | 0.292259 | 11.345045 | 2.37262 | 0.03130869 | 0.37952406 | -3.60752227 |
| 184 | Lrrfip2 | 0.2926099 | 7.3206504 | 2.369078 | 0.03152615 | 0.37986495 | -3.61349186 |
| 185 | Fam184b | 0.28211666 | 4.9864557 | 2.361712 | 0.03198294 | 0.38222369 | -3.62589471 |
| 186 | LOC100504944 | 0.28879038 | 5.2069208 | 2.357233 | 0.03226379 | 0.38414995 | -3.63342962 |
| 187 | Tmem82 | 0.3614685 | 4.8984448 | 2.345508 | 0.03300986 | 0.38605928 | -3.65312203 |
| 188 | Slc26a10 | 0.53457784 | 8.2648565 | 2.339454 | 0.03340145 | 0.38710241 | -3.66327452 |
| 189 | Eef2k | 0.28094271 | 6.637343 | 2.336611 | 0.03358684 | 0.38797503 | -3.66803822 |
| 190 | Olfr1466 | 0.32863562 | 10.887632 | 2.334953 | 0.03369543 | 0.3883962 | -3.67081583 |
| 191 | Pltp | 0.31816619 | 10.636541 | 2.330775 | 0.03397045 | 0.38950723 | -3.67780954 |
| 192 | Tssk5 | 0.36853652 | 5.2546562 | 2.325514 | 0.03431979 | 0.39182284 | -3.68660898 |
| 193 | Plcxd1 | 0.30228288 | 8.7285391 | 2.30311 | 0.03584558 | 0.39744589 | -3.72398541 |
| 194 | Prss28 | 0.45000212 | 5.1738001 | 2.302718 | 0.03587281 | 0.39744589 | -3.72463751 |
| 195 | Neurl2 | 0.27078165 | 9.0044227 | 2.3016 | 0.03595066 | 0.39794907 | -3.72649859 |
| 196 | Trim28 | 0.2901633 | 8.4367807 | 2.294863 | 0.03642301 | 0.39916796 | -3.73770237 |
| 197 | Ctrl | 0.27893611 | 6.0696686 | 2.288111 | 0.03690232 | 0.40083299 | -3.74891814 |
| 198 | AW549542 | 0.39625422 | 5.3309581 | 2.281609 | 0.03736945 | 0.40292823 | -3.75970474 |
| 199 | Birc7 | 0.26735732 | 4.9523838 | 2.279411 | 0.03752863 | 0.40326352 | -3.76334847 |
| 200 | Chid1 | 0.30527274 | 8.1030358 | 2.277636 | 0.0376576 | 0.40344325 | -3.76628883 |
| 201 | Olfr907 | 0.43676816 | 5.0175877 | 2.275339 | 0.03782513 | 0.40408537 | -3.77009305 |
| 202 | Wfikkn1 | 0.31751639 | 5.1881941 | 2.258581 | 0.0390687 | 0.410765 | -3.79779513 |
| 203 | Npcd | 0.35996099 | 11.790902 | 2.255637 | 0.03929105 | 0.4109425 | -3.80265189 |
| 204 | Fam71e1 | 0.27326226 | 9.878963 | 2.252678 | 0.03951581 | 0.41170085 | -3.80753208 |
| 205 | Myh7b | 0.28421086 | 8.5713352 | 2.239548 | 0.04052738 | 0.41580911 | -3.82914526 |
| 206 | 4933426M11Rik | 0.27147278 | 11.712268 | 2.234633 | 0.04091221 | 0.41719346 | -3.83722069 |
| 207 | AI854517 | 0.37947996 | 9.5370012 | 2.229231 | 0.04133917 | 0.41754076 | -3.84608828 |
| 208 | LOC677113 | 0.26663277 | 10.882625 | 2.226789 | 0.04153346 | 0.41778048 | -3.85009202 |
| 209 | Acy3 | 0.27081055 | 8.1490337 | 2.210022 | 0.04289102 | 0.42295711 | -3.87753482 |
| 210 | Gp5 | 0.36458959 | 5.1415191 | 2.207823 | 0.04307212 | 0.42361318 | -3.88112726 |
| 211 | Fanci | 0.65471214 | 4.9723245 | 2.205509 | 0.04326343 | 0.42426123 | -3.88490515 |
| 212 | Gm5101 | 0.38376183 | 6.0125435 | 2.198737 | 0.04382788 | 0.42565066 | -3.89595092 |
| 213 | Miip | 0.29681528 | 6.7723262 | 2.198251 | 0.04386863 | 0.42565066 | -3.89674256 |
| 214 | BC018473 | 0.30665692 | 5.4565259 | 2.195245 | 0.04412153 | 0.42611496 | -3.90163888 |
| 215 | Htra3 | 0.3618836 | 5.3172709 | 2.185195 | 0.0449771 | 0.43004356 | -3.91798914 |
| 216 | Tbc1d2b | 0.28764718 | 7.0697827 | 2.172996 | 0.04603625 | 0.43611383 | -3.93778596 |
| 217 | Ccnd2 | 0.27980496 | 12.255246 | 2.162197 | 0.04699305 | 0.44034434 | -3.95526542 |
| 218 | Golt1a | 0.62272117 | 5.1427423 | 2.159608 | 0.04722517 | 0.44070967 | -3.95944995 |
| 219 | Thsd4 | 0.45353438 | 7.2967049 | 2.149489 | 0.04814257 | 0.44259889 | -3.97578051 |
| 220 | Eif2s3y | 0.3774935 | 9.6480493 | 2.140398 | 0.04898078 | 0.44680768 | -3.99041955 |
| 221 | Olfr921 | 0.44725061 | 8.5953776 | 2.132392 | 0.04973013 | 0.45028851 | -4.0032865 |
| 222 | Cntn5 | 0.33721913 | 6.0959684 | 2.129806 | 0.0499744 | 0.45083332 | -4.00743711 |
| 223 | C78653 | -0.9607698 | 5.3489687 | -8.62307 | 3.12E-07 | 0.00688522 | 6.16953566 |
| 224 | Pcdhb18 | -0.3246402 | 7.7186377 | -7.02165 | 3.90E-06 | 0.03341465 | 4.195783714 |
| 225 | 4921531C22Rik | -0.408249 | 7.7440112 | -6.21862 | 1.57E-05 | 0.04940964 | 3.045846259 |
| 226 | Mthfd2l | -0.2723291 | 8.5349437 | -5.67003 | 4.28E-05 | 0.06282674 | 2.196220182 |
| 227 | Phka1 | -0.2875136 | 9.8048654 | -5.39453 | 7.18E-05 | 0.07195837 | 1.750324618 |
| 228 | Tnfaip8 | -0.3200326 | 7.7711411 | -5.06425 | 0.00013552 | 0.08784416 | 1.199579216 |
| 229 | Abcc4 | -0.29266 | 8.3941867 | -4.98768 | 0.00015731 | 0.09403222 | 1.06948775 |
| 230 | Kcnu1 | -0.337068 | 8.1116928 | -4.93207 | 0.00017538 | 0.09470365 | 0.974471909 |
| 231 | Magef1 | -0.3061077 | 9.9323304 | -4.67196 | 0.000293 | 0.12218204 | 0.524181137 |
| 232 | Tmco6 | -0.3100634 | 7.6478029 | -4.6658 | 0.00029661 | 0.12218204 | 0.51341148 |
| 233 | Edil3 | -0.3751912 | 8.3589402 | -4.61667 | 0.00032709 | 0.12218204 | 0.42729228 |
| 234 | Gm4673 | -0.294831 | 7.3937948 | -4.60076 | 0.00033763 | 0.12373795 | 0.399352272 |
| 235 | Glul | -0.2824035 | 15.0409 | -4.57787 | 0.00035342 | 0.12373795 | 0.359065097 |
| 236 | Foxs1 | -0.4923149 | 6.3216624 | -4.48919 | 0.00042207 | 0.12373795 | 0.20243621 |
| 237 | Fbxl3 | -0.3191985 | 8.4298938 | -4.48517 | 0.00042549 | 0.12373795 | 0.19531011 |
| 238 | Xkr6 | -0.3909605 | 7.8836705 | -4.44611 | 0.0004602 | 0.12373795 | 0.126013323 |
| 239 | Lztfl1 | -0.2899662 | 8.4807419 | -4.39843 | 0.00050655 | 0.12590537 | 0.041176781 |
| 240 | Kremen2 | -0.5035439 | 6.1721824 | -4.30896 | 0.00060682 | 0.13914103 | -0.1186878 |
| 241 | Ppargc1a | -0.2858633 | 9.5643014 | -4.29609 | 0.00062282 | 0.13914103 | -0.14174567 |
| 242 | Hnrnpm | -0.2855005 | 12.85819 | -4.2589 | 0.00067153 | 0.14295543 | -0.20848297 |
| 243 | Adam8 | -0.4417072 | 8.2430161 | -4.18819 | 0.00077516 | 0.14295543 | -0.3357629 |
| 244 | Gm5918 | -0.4278393 | 9.0908058 | -4.16241 | 0.00081687 | 0.14295543 | -0.38227572 |
| 245 | Id2 | -0.3146697 | 11.832383 | -4.14335 | 0.00084917 | 0.14295543 | -0.41669784 |
| 246 | Kank1 | -0.3129414 | 9.274454 | -4.12114 | 0.00088846 | 0.14295543 | -0.45685935 |
| 247 | 1700022A21Rik | -0.5806524 | 7.937677 | -4.08205 | 0.00096214 | 0.14623839 | -0.52762796 |
| 248 | Ezr | -0.3357832 | 11.334344 | -3.9783 | 0.00118931 | 0.15466473 | -0.71605591 |
| 249 | Nkapl | -0.2986993 | 5.4728634 | -3.97336 | 0.00120139 | 0.15466473 | -0.72503894 |
| 250 | Ankrd57 | -0.3572145 | 7.829921 | -3.9292 | 0.00131511 | 0.15466473 | -0.80548982 |
| 251 | Tmem45a | -0.7774472 | 5.585397 | -3.92214 | 0.00133427 | 0.15466473 | -0.8183644 |
| 252 | Hmgcll1 | -0.3466815 | 5.7955111 | -3.92176 | 0.0013353 | 0.15466473 | -0.8190495 |
| 253 | Tas1r3 | -0.4317812 | 5.4032843 | -3.91875 | 0.00134357 | 0.15466473 | -0.82454271 |
| 254 | D14Abb1e | -0.2872762 | 8.5594302 | -3.8759 | 0.00146698 | 0.1608234 | -0.90274111 |
| 255 | Wdr82 | -0.3244115 | 11.897399 | -3.86939 | 0.00148668 | 0.1608234 | -0.91461622 |
| 256 | Acot11 | -0.4023163 | 11.754571 | -3.86669 | 0.00149495 | 0.1608234 | -0.91955447 |
| 257 | Arntl | -0.2755897 | 9.8215779 | -3.8479 | 0.00155375 | 0.16500106 | -0.95389475 |
| 258 | 2310057B04Rik | -0.3420706 | 7.1618501 | -3.8468 | 0.00155725 | 0.16500106 | -0.95589778 |
| 259 | Gm7102 | -0.3958257 | 6.6899922 | -3.82986 | 0.00161237 | 0.16605163 | -0.98686457 |
| 260 | B3galt2 | -0.3095532 | 9.0193982 | -3.81966 | 0.00164649 | 0.16660325 | -1.00550969 |
| 261 | Trps1 | -0.2913585 | 9.7290119 | -3.81716 | 0.00165497 | 0.16660325 | -1.010081 |
| 262 | Yes1 | -0.5890235 | 5.3887212 | -3.80279 | 0.00170458 | 0.16678045 | -1.03637913 |
| 263 | Zfp799 | -0.3058668 | 6.851078 | -3.78515 | 0.00176749 | 0.16678045 | -1.06864983 |
| 264 | Saa1 | -0.2936763 | 7.3779196 | -3.76947 | 0.00182541 | 0.16696695 | -1.09736167 |
| 265 | Lrrk2 | -0.2707291 | 8.7422517 | -3.72694 | 0.0019923 | 0.17126572 | -1.1752769 |
| 266 | Zfp712 | -0.3120757 | 8.1866673 | -3.71109 | 0.00205834 | 0.17126572 | -1.20431875 |
| 267 | C1galt1c1 | -0.3295255 | 10.95804 | -3.69771 | 0.0021158 | 0.17126572 | -1.2288443 |
| 268 | Gjd2 | -0.2949687 | 6.6688498 | -3.66471 | 0.00226455 | 0.17554407 | -1.28936298 |
| 269 | Tmem167b | -0.2728618 | 9.94037 | -3.65646 | 0.00230339 | 0.17554603 | -1.30451094 |
| 270 | N4bp2 | -0.2754311 | 7.7595448 | -3.60165 | 0.00257869 | 0.18214442 | -1.40507705 |
| 271 | BC065397 | -0.3535516 | 5.9100003 | -3.57171 | 0.00274284 | 0.18538112 | -1.46004617 |
| 272 | Zfp760 | -0.3620986 | 8.9028787 | -3.56699 | 0.00276962 | 0.18553111 | -1.46870069 |
| 273 | Tubd1 | -0.4220811 | 8.3235164 | -3.55848 | 0.00281866 | 0.18654808 | -1.48433359 |
| 274 | Sat1 | -0.4369198 | 9.829821 | -3.54498 | 0.00289821 | 0.18810284 | -1.5091221 |
| 275 | Fam110c | -0.3493793 | 5.171008 | -3.48613 | 0.00327205 | 0.19224608 | -1.61717087 |
| 276 | Tll1 | -0.2721532 | 7.1397561 | -3.47476 | 0.00334963 | 0.19224608 | -1.63803772 |
| 277 | Ccdc151 | -0.3522427 | 8.5878783 | -3.44709 | 0.00354626 | 0.19490256 | -1.68882767 |
| 278 | Prpf40a | -0.2685037 | 7.5820638 | -3.44388 | 0.00356982 | 0.19519144 | -1.69472387 |
| 279 | Gm6252 | -0.424963 | 11.538039 | -3.43104 | 0.00366561 | 0.19608199 | -1.71829802 |
| 280 | LOC100039024 | -0.3806273 | 6.3912109 | -3.41996 | 0.00375032 | 0.19608199 | -1.73863651 |
| 281 | Xpo1 | -0.2806774 | 10.054547 | -3.41465 | 0.00379159 | 0.19608199 | -1.74837942 |
| 282 | LOC100504004 | -0.3691213 | 13.50593 | -3.38909 | 0.00399673 | 0.19883486 | -1.79527705 |
| 283 | Gin1 | -0.2659125 | 6.7616225 | -3.38462 | 0.00403368 | 0.20022151 | -1.80346883 |
| 284 | Fndc3b | -0.3518982 | 8.2209959 | -3.36612 | 0.00419047 | 0.20212283 | -1.83740315 |
| 285 | Odf2l | -0.4465882 | 5.271483 | -3.35291 | 0.00430609 | 0.20365196 | -1.86162024 |
| 286 | Aass | -0.3372688 | 9.3985683 | -3.31972 | 0.00461085 | 0.20836841 | -1.92245418 |
| 287 | Cgrrf1 | -0.2901986 | 9.7812734 | -3.31253 | 0.00467965 | 0.20836841 | -1.93562779 |
| 288 | Sirt4 | -0.3292763 | 8.2092131 | -3.30483 | 0.00475445 | 0.2092081 | -1.94973193 |
| 289 | Pcmtd2 | -0.2786603 | 10.530745 | -3.30237 | 0.00477865 | 0.2092081 | -1.95424713 |
| 290 | Zfp110 | -0.3748467 | 7.6092768 | -3.30177 | 0.00478451 | 0.2092081 | -1.95533556 |
| 291 | Neu3 | -0.4146487 | 6.2128062 | -3.29914 | 0.00481054 | 0.2092081 | -1.9601612 |
| 292 | Gm10845 | -0.3184291 | 11.770593 | -3.29427 | 0.00485896 | 0.20980408 | -1.9690676 |
| 293 | Fzd6 | -0.3562319 | 6.9619069 | -3.27723 | 0.00503247 | 0.21255457 | -2.00026553 |
| 294 | Ttc32 | -0.4059153 | 7.3457844 | -3.27289 | 0.00507762 | 0.2134274 | -2.00820576 |
| 295 | D630039A03Rik | -0.6242129 | 5.3785189 | -3.26549 | 0.00515564 | 0.21479237 | -2.02176164 |
| 296 | Klhl24 | -0.2664737 | 8.0242617 | -3.26076 | 0.0052061 | 0.21567154 | -2.03041964 |
| 297 | Ssrp1 | -0.2952162 | 9.7487578 | -3.25838 | 0.00523157 | 0.21591557 | -2.0347577 |
| 298 | Ggcx | -0.2703661 | 7.3433457 | -3.25836 | 0.0052318 | 0.21591557 | -2.0347965 |
| 299 | Cenpq | -0.3376667 | 7.2035167 | -3.25026 | 0.00531976 | 0.21591557 | -2.0496158 |
| 300 | Tmem107 | -0.2974614 | 10.060847 | -3.21962 | 0.00566596 | 0.22090006 | -2.10564147 |
| 301 | 1700029I01Rik | -0.3399686 | 5.2598473 | -3.19545 | 0.00595464 | 0.2235677 | -2.14978464 |
| 302 | Wwtr1 | -0.3246686 | 8.122079 | -3.19192 | 0.00599801 | 0.2244314 | -2.15622988 |
| 303 | Cldn5 | -0.3589547 | 11.991082 | -3.15448 | 0.00647767 | 0.22856173 | -2.22453558 |
| 304 | Malt1 | -0.2709348 | 6.2528083 | -3.1398 | 0.00667599 | 0.23225239 | -2.25129868 |
| 305 | Pcnx | -0.2924268 | 11.415601 | -3.11388 | 0.00704075 | 0.23441328 | -2.29849445 |
| 306 | Flrt3 | -0.2735915 | 10.518749 | -3.1064 | 0.00714972 | 0.23568614 | -2.312116 |
| 307 | Hexb | -0.2815036 | 12.701749 | -3.05288 | 0.00797876 | 0.24679255 | -2.4093652 |
| 308 | St3gal4 | -0.3349081 | 7.7537868 | -3.04104 | 0.00817451 | 0.24891594 | -2.43083648 |
| 309 | Agmat | -0.3521691 | 7.3921986 | -3.03892 | 0.00821001 | 0.24891594 | -2.43467461 |
| 310 | Nfil3 | -0.3347094 | 10.276956 | -3.03891 | 0.00821015 | 0.24891594 | -2.43468991 |
| 311 | Clock | -0.3330283 | 8.583893 | -3.03077 | 0.0083481 | 0.25004953 | -2.44944658 |
| 312 | Pcgf6 | -0.2991578 | 8.8879683 | -3.01992 | 0.00853553 | 0.25006696 | -2.46910678 |
| 313 | Bcar3 | -0.3351987 | 9.1383244 | -3.01932 | 0.00854611 | 0.25006696 | -2.47020302 |
| 314 | Aldh8a1 | -0.2638987 | 6.1098894 | -3.0145 | 0.00863081 | 0.25094251 | -2.47893432 |
| 315 | 4930417G10Rik | -0.3545207 | 5.0624729 | -3.01346 | 0.0086491 | 0.25099202 | -2.48080833 |
| 316 | 1700007K13Rik | -0.4451912 | 5.8208777 | -3.00664 | 0.00877052 | 0.25128139 | -2.49314832 |
| 317 | Gm3065 | -0.384304 | 6.1787437 | -2.99572 | 0.00896852 | 0.25277514 | -2.5129049 |
| 318 | Syt4 | -0.2632041 | 10.857291 | -2.987 | 0.00912985 | 0.25534605 | -2.52867864 |
| 319 | Gja1 | -0.2739257 | 9.3287953 | -2.97039 | 0.00944485 | 0.25889922 | -2.55868166 |
| 320 | Sall1 | -0.3396383 | 7.3091998 | -2.95994 | 0.0096486 | 0.26115203 | -2.57755414 |
| 321 | Kdr | -0.3345527 | 8.7149945 | -2.94587 | 0.00992965 | 0.26257551 | -2.60293491 |
| 322 | Htra1 | -0.3055704 | 15.032298 | -2.94163 | 0.0100159 | 0.26259944 | -2.61057785 |
| 323 | Afap1l2 | -0.2662489 | 7.5742385 | -2.93055 | 0.01024462 | 0.26504328 | -2.63052817 |
| 324 | Casp4 | -0.4020716 | 5.3642256 | -2.92945 | 0.01026763 | 0.26504328 | -2.63251047 |
| 325 | Galnt1 | -0.2746912 | 8.2954711 | -2.91373 | 0.01060185 | 0.26653467 | -2.66080234 |
| 326 | Wdr44 | -0.433136 | 6.6734408 | -2.91316 | 0.01061417 | 0.26653467 | -2.66182829 |
| 327 | L3mbtl3 | -0.3123696 | 9.3040612 | -2.91098 | 0.01066138 | 0.26662252 | -2.66574705 |
| 328 | Sntb2 | -0.337854 | 8.4554034 | -2.90293 | 0.01083757 | 0.2678406 | -2.68021896 |
| 329 | 1700069B07Rik | -0.3797856 | 5.3009488 | -2.9028 | 0.0108405 | 0.2678406 | -2.68045734 |
| 330 | Gpr17 | -0.3781167 | 12.470125 | -2.89767 | 0.01095438 | 0.26823892 | -2.68968186 |
| 331 | Mesdc1 | -0.3070229 | 5.4140108 | -2.86293 | 0.01175608 | 0.27533705 | -2.7519954 |
| 332 | Camp | -0.3333629 | 7.3510393 | -2.85787 | 0.01187756 | 0.27654471 | -2.76106 |
| 333 | Zfp709 | -0.3627459 | 5.220157 | -2.85627 | 0.01191623 | 0.27654471 | -2.76392573 |
| 334 | Ctsc | -0.2689041 | 7.8966943 | -2.84078 | 0.01229701 | 0.27654471 | -2.79165074 |
| 335 | Dapk2 | -0.4287349 | 6.3391073 | -2.81014 | 0.0130852 | 0.2832119 | -2.84637036 |
| 336 | Acot1 | -0.3403929 | 11.204468 | -2.80383 | 0.01325363 | 0.2855967 | -2.85762815 |
| 337 | Irf2bp2 | -0.328295 | 11.489656 | -2.7955 | 0.01347899 | 0.28702258 | -2.87246654 |
| 338 | Gm11213 | -0.4232836 | 7.6767804 | -2.79497 | 0.01349345 | 0.28702258 | -2.8734106 |
| 339 | Nrg1 | -0.3130796 | 9.5936844 | -2.79109 | 0.01359985 | 0.28705459 | -2.88032082 |
| 340 | Prss16 | -0.500105 | 6.3637875 | -2.76552 | 0.01432189 | 0.29389203 | -2.92581371 |
| 341 | Rab13 | -0.2982797 | 5.4494642 | -2.75951 | 0.01449679 | 0.29482938 | -2.9364829 |
| 342 | Nudt7 | -0.2854606 | 6.7072383 | -2.75658 | 0.01458287 | 0.29482938 | -2.9416853 |
| 343 | Pik3r1 | -0.3195773 | 9.2830119 | -2.74785 | 0.01484221 | 0.29638191 | -2.95717321 |
| 344 | Thumpd2 | -0.3291055 | 6.2193464 | -2.73438 | 0.01525104 | 0.29983742 | -2.98103852 |
| 345 | Sc5d | -0.266315 | 10.066816 | -2.72783 | 0.0154538 | 0.30034048 | -2.9926337 |
| 346 | Cd274 | -0.3813214 | 5.8992364 | -2.71079 | 0.01599355 | 0.30570852 | -3.02276101 |
| 347 | E130114P18Rik | -0.3174099 | 6.3805175 | -2.70608 | 0.01614606 | 0.30676113 | -3.03108566 |
| 348 | Gm6403 | -0.4701963 | 5.4858977 | -2.70433 | 0.01620289 | 0.30704685 | -3.03416762 |
| 349 | Pkia | -0.2957496 | 11.400354 | -2.70138 | 0.01629946 | 0.3080822 | -3.03937927 |
| 350 | Wnt10a | -0.2755073 | 10.469673 | -2.69636 | 0.01646473 | 0.30841557 | -3.04822577 |
| 351 | Cthrc1 | -0.3050364 | 7.2959469 | -2.68393 | 0.01688155 | 0.31117635 | -3.07014038 |
| 352 | Cmpk1 | -0.2949327 | 9.8459086 | -2.67758 | 0.01709837 | 0.3116881 | -3.08132297 |
| 353 | Fst | -0.4095541 | 8.0875218 | -2.64952 | 0.01808879 | 0.3177612 | -3.13062922 |
| 354 | Sesn3 | -0.3529301 | 9.7428162 | -2.64804 | 0.01814249 | 0.3177612 | -3.13322362 |
| 355 | Ccdc75 | -0.2667347 | 9.0094298 | -2.64192 | 0.01836664 | 0.31779396 | -3.1439673 |
| 356 | Casp8ap2 | -0.2954804 | 7.8760488 | -2.61765 | 0.01928104 | 0.32712464 | -3.18645048 |
| 357 | Tgds | -0.2700023 | 5.9103659 | -2.61297 | 0.01946227 | 0.32792739 | -3.19462558 |
| 358 | LOC100316870 | -0.3049169 | 12.314498 | -2.57682 | 0.02091864 | 0.33774798 | -3.25762553 |
| 359 | Zfp654 | -0.3024038 | 5.7753743 | -2.56727 | 0.02132047 | 0.34000384 | -3.27421893 |
| 360 | Cdk15 | -0.3640266 | 5.2924464 | -2.55993 | 0.02163458 | 0.34265563 | -3.28696825 |
| 361 | AI849053 | -0.5426877 | 5.7983365 | -2.55445 | 0.02187167 | 0.34308169 | -3.29646682 |
| 362 | Emp2 | -0.2810676 | 9.3258991 | -2.54219 | 0.02241146 | 0.34661483 | -3.31770431 |
| 363 | Fcgr2b | -0.3632977 | 6.150446 | -2.54063 | 0.02248114 | 0.34732152 | -3.32040744 |
| 364 | Arrdc3 | -0.3288876 | 8.1331017 | -2.48855 | 0.0249264 | 0.36284877 | -3.41019613 |
| 365 | S100a8 | -0.6424191 | 6.3324238 | -2.47583 | 0.02556113 | 0.36486915 | -3.43202538 |
| 366 | Creb3l4 | -0.3007105 | 4.9664401 | -2.46381 | 0.02617471 | 0.36505426 | -3.45260423 |
| 367 | Rsbn1l | -0.271022 | 6.8986973 | -2.46356 | 0.0261877 | 0.36505426 | -3.45303478 |
| 368 | Crh | -0.3602145 | 8.273596 | -2.45395 | 0.02668874 | 0.36830498 | -3.46946616 |
| 369 | 5430400D12Rik | -0.2676703 | 7.2649939 | -2.45062 | 0.02686437 | 0.36888709 | -3.47515092 |
| 370 | Sdc4 | -0.3201849 | 8.931857 | -2.44879 | 0.0269617 | 0.36930321 | -3.47828489 |
| 371 | Klrg1 | -0.4054667 | 4.912257 | -2.4445 | 0.02719047 | 0.36971952 | -3.48560543 |
| 372 | Fignl1 | -0.2803809 | 5.7880375 | -2.44398 | 0.02721845 | 0.36971952 | -3.48649662 |
| 373 | C1galt1 | -0.3073524 | 6.0738656 | -2.44332 | 0.02725365 | 0.36971952 | -3.48761598 |
| 374 | Oas1f | -0.2852547 | 5.741691 | -2.44308 | 0.02726647 | 0.36971952 | -3.48802354 |
| 375 | Tspan15 | -0.2799695 | 7.0457345 | -2.4423 | 0.0273086 | 0.36971952 | -3.48936102 |
| 376 | Heyl | -0.4971054 | 5.9615404 | -2.43953 | 0.02745789 | 0.37035031 | -3.49408302 |
| 377 | LOC434825 | -0.499737 | 5.3293471 | -2.43029 | 0.0279618 | 0.37371146 | -3.50982895 |
| 378 | S100a9 | -0.8573194 | 8.5987756 | -2.42817 | 0.02807853 | 0.3740544 | -3.51343456 |
| 379 | 2310008H04Rik | -0.2752008 | 7.9561238 | -2.41926 | 0.02857441 | 0.37413938 | -3.52858248 |
| 380 | Fam43a | -0.3116293 | 8.3689551 | -2.41747 | 0.02867479 | 0.37413938 | -3.53161566 |
| 381 | 4930578N16Rik | -0.3426489 | 6.8287195 | -2.41618 | 0.02874754 | 0.37413938 | -3.53380733 |
| 382 | Hnrnpc | -0.3144622 | 12.501216 | -2.40981 | 0.02910934 | 0.37413938 | -3.54462235 |
| 383 | Chst5 | -0.3370875 | 6.6820212 | -2.40729 | 0.02925351 | 0.37450865 | -3.54889319 |
| 384 | Trpc6 | -0.3116069 | 9.1693509 | -2.40685 | 0.02927893 | 0.37450865 | -3.54964417 |
| 385 | Gadd45a | -0.3900475 | 7.9714594 | -2.39827 | 0.02977597 | 0.37606512 | -3.56419125 |
| 386 | Asap3 | -0.3182307 | 6.5009345 | -2.37555 | 0.03113016 | 0.37946771 | -3.60258903 |
| 387 | Stk17b | -0.269149 | 7.0853093 | -2.36753 | 0.03162184 | 0.38027693 | -3.61610532 |
| 388 | Fam111a | -0.3604055 | 5.1455502 | -2.36201 | 0.03196447 | 0.38222369 | -3.62539661 |
| 389 | Rimbp2 | -0.3548542 | 5.47495 | -2.34459 | 0.03306872 | 0.38608249 | -3.65465608 |
| 390 | 5830433M19Rik | -0.3455765 | 6.6804244 | -2.3271 | 0.03421381 | 0.39150475 | -3.68394931 |
| 391 | LOC100505360 | -0.2646851 | 10.544859 | -2.32034 | 0.03466658 | 0.39360564 | -3.69525319 |
| 392 | 4932443I19Rik | -0.3963947 | 6.5356362 | -2.3144 | 0.03506856 | 0.39573783 | -3.7051616 |
| 393 | 4930525F21Rik | -0.3324747 | 8.4039705 | -2.2997 | 0.03608317 | 0.39842618 | -3.729657 |
| 394 | Mid2 | -0.3816694 | 5.9980595 | -2.2987 | 0.03615297 | 0.3985905 | -3.73131567 |
| 395 | Lingo4 | -0.2957562 | 7.8000286 | -2.28232 | 0.03731823 | 0.40291428 | -3.75852883 |
| 396 | Prap1 | -0.3425365 | 5.0314337 | -2.27903 | 0.03755596 | 0.40336049 | -3.76397238 |
| 397 | Plau | -0.3982671 | 6.6306581 | -2.27249 | 0.03803358 | 0.40570509 | -3.7748019 |
| 398 | Sv2c | -0.3937609 | 6.8210214 | -2.24486 | 0.04011504 | 0.41455362 | -3.82040368 |
| 399 | Lpin3 | -0.3241043 | 5.8034704 | -2.22054 | 0.04203495 | 0.41986273 | -3.86033681 |
| 400 | Rbm12b | -0.2722453 | 7.5477948 | -2.21847 | 0.0422021 | 0.42069231 | -3.86372335 |
| 401 | Rint1 | -0.2738624 | 5.6104893 | -2.21565 | 0.04243102 | 0.42161283 | -3.86833859 |
| 402 | Ptpn21 | -0.3488037 | 6.2947746 | -2.19852 | 0.04384612 | 0.42565066 | -3.89630547 |
| 403 | Dnajc28 | -0.3058165 | 7.5659054 | -2.19647 | 0.04401846 | 0.42565066 | -3.89964697 |
| 404 | Bbs10 | -0.3088291 | 6.8253082 | -2.19604 | 0.04405414 | 0.42565066 | -3.90033704 |
| 405 | Refbp2 | -0.2827807 | 8.5333322 | -2.18304 | 0.0451629 | 0.43123628 | -3.92149684 |
| 406 | Gm4354 | -0.4428373 | 9.0682685 | -2.16966 | 0.04632958 | 0.4370394 | -3.94318482 |
| 407 | C030044M21Rik | -0.4492895 | 6.3738341 | -2.15764 | 0.04740206 | 0.44154441 | -3.96262449 |
| 408 | Foxn3 | -0.2699851 | 8.8021834 | -2.15496 | 0.04764465 | 0.44225277 | -3.9669579 |
| 409 | Slc18a3 | -0.367819 | 5.2414129 | -2.15336 | 0.04778959 | 0.44259889 | -3.96953607 |
| 410 | Elavl4 | -0.2793414 | 10.670994 | -2.15011 | 0.04808557 | 0.44259889 | -3.97477551 |

**Table S3. The enriched GO terms of the key differentially expressed genes in DG and ACC**

| **Sample** | **gene expression** | **Category** | **Term** | **P-value** | **Count** | **Genes** |
| --- | --- | --- | --- | --- | --- | --- |
| DG | up | biological processes (BP) | transport（GO:0006810） | 0.0346808 | 62 | SLC45A3, SLC22A18, SLC6A20A, ZMAT3, SLC16A12, CRABP2, AMN, KCNJ12, UQCRQ, KCNJ13, SLC25A22, CHRNA4, ATP5O, ANO4, TRPV6, KCNQ1, SLC22A2, SLC22A1, CHRNA2, ND4, OBP1A, SLC22A7, ND3, IPO8, CNGA2, CATSPER4, KCNT1, GABRR1, PITPNM3, PTGDS, TOMM22, KPNA4, AKAP8, ARL4C, KCNH5, ENOX1, CPLX3, RBP4, SDAD1, STAM2, RBP3, ASTN2, FXYD5, KCNA7, LRRC38, PEX14, SNX24, RLBP1, HCN1, MUP4, GABRA1, RABIF, GABRA3, SLC6A12, SLC6A13, DENND1A, SLC16A3, SLC4A10, COX2, TSPO2, RAB36, SLC13A3 |
| DG | up | biological processes (BP) | cell adhesion（GO:0007155） | 1.04E-04 | 29 | SIGLECE, MPZL2, PTK7, CDH1, RADIL, NOV, CDH7, CDH20, CTGF, FAP, FAT2, CD22, THBS1, KLRA1, SPON2, CDH26, RS1, FN1, SVEP1, CDHR2, NLGN1, COL5A1, LAMA4, OMD, SRPX2, LAMC3, HAS1, EPHA8, LAMC2 |
| DG | up | biological processes (BP) | proteolysis（GO:0006508） | 0.0381388 | 24 | AEBP1, PRSS53, CYM, USP8, MMEL1, RBP3, OVCH2, DPP10, PAMR1, ELANE, F9, ANPEP, OTUD1, CAPN11, CASP14, FAP, PRSS28, C1RL, ADAM32, ADAM33, C2, CASP1, ADAMTS2, TLL1 |
| DG | up | biological processes (BP) | ion transport（GO:0006811） | 0.0394581 | 24 | HCN1, SLC22A18, GABRA1, GABRA3, SLC22A7, KCNJ12, FXYD5, KCNA7, KCNJ13, LRRC38, CNGA2, SLC4A10, CATSPER4, GABRR1, KCNT1, CHRNA4, ATP5O, SLC13A3, TRPV6, KCNQ1, SLC22A2, CHRNA2, SLC22A1, KCNH5 |
| DG | up | biological processes (BP) | transmembrane transport（GO:0055085） | 0.0041073 | 20 | HCN1, SLC45A3, SLC22A18, SLC22A7, SLC16A12, IPO8, KCNA7, CNGA2, SLC16A3, CATSPER4, SLC25A22, SLC18A3, SLC22A6, SLC13A3, TRPV6, SLC13A4, KCNQ1, SLC22A2, SLC22A1, KCNH5 |
| DG | up | biological processes (BP) | immune system process（GO:0002376） | 0.0499128 | 17 | APOBEC3, CD244, HMGB3, GBP5, CD3E, ANXA1, OAS2, CD74, IFIT1, KRT16, H2-EB1, PYCARD, C1RL, AKAP8, C2, SPON2, CD7 |
| DG | up | biological processes (BP) | response to lipopolysaccharide（GO:0032496） | 0.0023821 | 14 | ALPL, PTGER3, LTBR, STAT5B, ELANE, DUSP10, CXCL11, THBD, NLRC3, PENK, CNR2, NOS2, CASP1, SPON2 |
| DG | up | biological processes (BP) | chemical synaptic transmission（GO:0007268） | 0.0167287 | 11 | SSTR5, PENK, GABRA3, RAPSN, SYT10, PMCH, SLC6A12, SLC6A13, SLC18A3, UNC13C, CHAT |
| DG | up | biological processes (BP) | blood vessel development（GO:0001568） | 1.05E-04 | 10 | DLX3, ALDH1A2, LAMA4, SPHK1, COL1A2, FOXC2, FOXC1, COL1A1, RAPGEF1, COL5A1 |
| DG | up | biological processes (BP) | response to peptide hormone（GO:0043434） | 1.31E-04 | 10 | CTGF, SERPINA1A, SERPINA1D, SERPINA1C, STAT5B, ANXA1, SERPINA1E, COL1A1, BMP7, TIMP1 |
| DG | up | biological processes (BP) | glucose homeostasis（GO:0042593） | 0.0091071 | 10 | RBP4, SSTR5, MUP4, WFS1, PMCH, MUP19, LEPR, MUP2, GCGR, BGLAP2 |
| DG | up | biological processes (BP) | skeletal system development（GO:0001501） | 0.0076742 | 9 | WFIKKN2, COL1A2, FOXC2, ROR2, FOXC1, HES7, COL1A1, MEPE, TRAPPC2 |
| DG | up | biological processes (BP) | axon guidance（GO:0007411） | 0.0454629 | 9 | KIF5B, EPHA8, SEMA3F, LMTK2, EFNA5, SEMA3A, BMP7, FOXD1, GAP43 |
| DG | up | biological processes (BP) | negative regulation of peptidase activity（GO:0010466） | 0.0364611 | 8 | WFIKKN2, SERPINA11, SERPINA1C, SERPINA1E, SERPIND1, SERPINA3B, TIMP1, WFDC13 |
| DG | up | biological processes (BP) | collagen fibril organization（GO:0030199） | 5.26E-04 | 7 | FMOD, COL1A2, FOXC2, FOXC1, COL1A1, ADAMTS2, COL5A1 |
| DG | up | biological processes (BP) | neurotransmitter transport（GO:0006836） | 0.0065811 | 6 | CPLX3, SLC6A20A, SLC6A12, SLC6A13, SLC18A3, SLC22A1 |
| DG | up | biological processes (BP) | muscle contraction（GO:0006936） | 0.0102484 | 6 | TNNT2, MYL6B, MYBPC1, MYH2, ANXA1, MYH4 |
| DG | up | biological processes (BP) | response to calcium ion（GO:0051592） | 0.034346 | 6 | HCN1, TNNT2, SDC1, PENK, TRPV6, THBS1 |
| DG | up | biological processes (BP) | negative regulation of insulin secretion（GO:0046676） | 0.0228918 | 5 | NOV, MIDN, PTGER3, PDE4C, KCNQ1 |
| DG | up | biological processes (BP) | ureteric bud development（GO:0001657） | 0.0381092 | 5 | TSHZ3, SDC1, FOXC2, FOXC1, BMP7 |
| DG | up | biological processes (BP) | cellular response to cAMP（GO:0071320） | 0.0433034 | 5 | HCN1, PENK, AKAP7, RAPGEF1, KCNQ1 |
| DG | up | biological processes (BP) | response to cAMP（GO:0051591） | 0.0460423 | 5 | SDC1, THBD, COL1A1, PPARGC1B, ALDH3A1 |
| DG | up | biological processes (BP) | actin crosslink formation（GO:0051764） | 0.0034202 | 4 | TNNT2, COBL, BAIAP2L2, BAIAP2L1 |
| DG | up | biological processes (BP) | prostaglandin biosynthetic process（GO:0001516） | 0.0080462 | 4 | PNPLA8, PTGIS, PTGDS, CD74 |
| DG | up | biological processes (BP) | positive regulation of synaptic transmission, GABAergic（GO:0032230） | 0.0080462 | 4 | KIF5B, CCKBR, NLGN1, CAR7 |
| DG | up | biological processes (BP) | astrocyte development（GO:0014002） | 0.0095804 | 4 | LAMC3, VIM, ROR2, POU3F2 |
| DG | up | biological processes (BP) | positive regulation of glucose metabolic process（GO:0010907） | 0.0173276 | 4 | SLC45A3, MUP4, MUP19, MUP2 |
| DG | up | biological processes (BP) | negative regulation of gluconeogenesis（GO:0045721） | 0.0248735 | 4 | MUP4, MUP19, LEPR, MUP2 |
| DG | up | biological processes (BP) | locomotor rhythm（GO:0045475） | 0.0307365 | 4 | MUP4, MUP19, MUP2, MC3R |
| DG | up | biological processes (BP) | negative regulation of myeloid cell differentiation（GO:0045638） | 0.0372589 | 4 | MEIS2, HMGB3, LEO1, GM13304 |
| DG | up | biological processes (BP) | epoxygenase P450 pathway（GO:0019373） | 0.0444292 | 4 | CYP2F2, CYP4F18, CYP2T4, CYP2B10 |
| DG | up | biological processes (BP) | synaptic transmission, cholinergic（GO:0007271） | 0.0444292 | 4 | RAPSN, HRH4, CHRNA4, CHRNA2 |
| DG | up | biological processes (BP) | neural crest cell migration involved in autonomic nervous system development（GO:1901166） | 0.0135171 | 3 | SEMA3F, SEMA3A, FN1 |
| DG | up | biological processes (BP) | positive regulation of execution phase of apoptosis（GO:1900119） | 0.0223724 | 3 | PTGIS, TRP53BP2, FAP |
| DG | up | biological processes (BP) | branchiomotor neuron axon guidance（GO:0021785） | 0.0330056 | 3 | SEMA3F, SEMA3A, PLXND1 |
| DG | up | biological processes (BP) | type B pancreatic cell proliferation（GO:0044342） | 0.0452069 | 3 | NOV, IGFBP4, BGLAP2 |
| DG | up | biological processes (BP) | nerve development（GO:0021675） | 0.0452069 | 3 | NRTN, SEMA3F, SEMA3A |
| DG | up | biological processes (BP) | semaphorin-plexin signaling pathway involved in axon guidance（GO:1902287） | 0.0452069 | 3 | SEMA3F, SEMA3A, PLXND1 |
| DG | up | molecular function (MF) | calcium ion binding（GO:0005509) | 0.0072176 | 32 | LALBA, PRF1, TNNC2, SYT2, PAMR1, CDH1, CABP5, ASTN2, NECAB3, CDH7, CDH20, MYL6B, DNER, FAT2, PLCH1, THBS1, CDH26, BGLAP2, SVEP1, SYT10, CDHR2, S100A11, ANXA1, F9, PADI2, PADI1, THBD, CAPN11, CDH18, SYTL2, SYTL1, TLL1 |
| DG | up | molecular function (MF) | calmodulin binding（GO:0005516) | 0.0017717 | 14 | AEBP1, WFS1, ADCY8, SPHK1, MYH4, CNGA2, IQCG, TRPV6, NOS2, UNC13C, KCNQ1, GAP43, MAP6D1, KCNH5 |
| DG | up | molecular function (MF) | transporter activity（GO:0005215) | 0.0078475 | 14 | RBP4, MUP4, SLC22A18, OBP1A, MUP19, MUP2, CRABP2, SLC4A10, PTGDS, SLC13A3, SLC13A4, RLBP1, SLC22A2, SLC22A1 |
| DG | up | molecular function (MF) | ion channel activity（GO:0005216) | 0.008072 | 12 | HCN1, CATSPER4, GABRA1, GABRA3, CHRNA4, TRPV6, FXYD5, KCNA7, KCNQ1, CHRNA2, CNGA2, KCNH5 |
| DG | up | molecular function (MF) | protease binding（GO:0002020) | 0.0015774 | 11 | ADRM1, SERPINA1A, SERPINA1D, FAP, ELANE, SERPINA1C, PYCARD, PINK1, SERPINA1E, FN1, TIMP1 |
| DG | up | molecular function (MF) | heparin binding（GO:0008201) | 0.0096647 | 11 | NOV, FMOD, CTGF, ELANE, LAMC2, SERPIND1, THBS1, CXCL11, BMP7, COL5A1, FN1 |
| DG | up | molecular function (MF) | serine-type endopeptidase inhibitor activity（GO:0004867) | 0.0074106 | 10 | WFIKKN2, SERPINA11, SERPINA1A, SERPINA1D, SERPINB6C, SERPINA1C, SERPINA1E, SERPIND1, SERPINA3B, WFDC13 |
| DG | up | molecular function (MF) | serine-type peptidase activity（GO:0008236) | 0.0386775 | 10 | PRSS53, RBP3, FAP, OVCH2, PRSS28, ELANE, DPP10, C1RL, F9, C2 |
| DG | up | molecular function (MF) | symporter activity（GO:0015293) | 0.0129182 | 9 | SLC16A3, SLC4A10, SLC22A18, SLC6A20A, SLC6A12, SLC16A12, SLC6A13, SLC25A22, SLC13A3 |
| DG | up | molecular function (MF) | SH3 domain binding（GO:0017124) | 0.046606 | 8 | USP8, CD3E, TRP53BP2, PLSCR2, ARHGAP1, DENND1A, ASAP1, ELMO1 |
| DG | up | molecular function (MF) | phosphatidylserine binding（GO:0001786) | 6.70E-04 | 7 | SYT10, SYT2, ASAP1, SYTL2, THBS1, GAP43, RS1 |
| DG | up | molecular function (MF) | glycoprotein binding（GO:0001948) | 0.0225927 | 7 | SDC1, SERPINA1A, SERPINA1D, VIM, SERPINA1C, SERPINA1E, CDH1 |
| DG | up | molecular function (MF) | phosphatidylinositol-4,5-bisphosphate binding（GO:0005546) | 0.0270655 | 6 | HCN1, SYT10, ASAP1, SYTL2, KCNQ1, RS1 |
| DG | up | molecular function (MF) | voltage-gated potassium channel activity（GO:0005249) | 0.045099 | 6 | HCN1, KCNT1, KCNA7, KCNQ1, CNGA2, KCNH5 |
| DG | up | molecular function (MF) | retinal binding（GO:0016918) | 1.67E-04 | 5 | RBP4, ALDH1A2, RBP3, CRABP2, RLBP1 |
| DG | up | molecular function (MF) | small molecule binding（GO:0036094) | 0.0114537 | 5 | RBP4, MUP4, PTGDS, MUP19, MUP2 |
| DG | up | molecular function (MF) | extracellular ligand-gated ion channel activity（GO:0005230) | 0.0265548 | 5 | GABRR1, GABRA1, GABRA3, CHRNA4, CHRNA2 |
| DG | up | molecular function (MF) | calcium-dependent phospholipid binding（GO:0005544) | 0.0411274 | 5 | SYT10, SYT2, ANXA1, SYTL2, SYTL1 |
| DG | up | molecular function (MF) | retinol binding（GO:0019841) | 0.0038953 | 4 | RBP4, RBP3, CRABP2, RLBP1 |
| DG | up | molecular function (MF) | insulin-like growth factor binding（GO:0005520) | 0.0250203 | 4 | NOV, CTGF, IGFBP6, IGFBP4 |
| DG | up | molecular function (MF) | chemorepellent activity（GO:0045499) | 0.0381025 | 4 | SEMA3F, SEMA3B, EFNA5, SEMA3A |
| DG | up | molecular function (MF) | retinoid binding（GO:0005501) | 0.0193247 | 3 | RBP4, PTGDS, RBP3 |
| DG | up | molecular function (MF) | neuropilin binding（GO:0038191) | 0.0193247 | 3 | SEMA3F, SEMA3B, SEMA3A |
| DG | up | molecular function (MF) | BMP receptor binding（GO:0070700) | 0.0359289 | 3 | BMP3, PYCARD, BMP7 |
| DG | up | molecular function (MF) | platelet-derived growth factor binding（GO:0048407) | 0.0423356 | 3 | COL1A2, COL1A1, COL5A1 |
| DG | up | molecular function (MF) | insulin-activated receptor activity(GO:0005009) | 0.0491311 | 3 | MUP4, MUP19, MUP2 |
| DG | up | cellular component (CC) | membrane（GO:0016020） | 0.0100604 | 208 | LYPD2, LYPD5, ADCY8, SYT2, PRKG2, KLHDC7A, CDH20, PIP5KL1, FAP, CHRNA4, CDH26, CHRNA2, GBP5, KIF5B, CD3E, CDHR2, COLEC12, SSTR5, CATSPER4, PITPNM3, HAS1, VSIG8, ROR2, EFNA5, LAMC2, UNC13C, RTP2, LMNB2, ASAP1, TAS2R113, VMN1R48, OAS2, COIL, CD74, VRK2, SLC28A1, CDC42EP3, GPR157, SPHK1, DENND1A, CAMK2N1, CYP17A1, TAS2R109, COX3, COX2, RAPSN, TSPO2, EPHA8, SYTL2, CYP2R1, UTS2R, SYTL1, SLC16A12, DPP10, ANPEP, KCNJ12, GPR88, INSRR, KCNJ13, ASGR1, AGPAT9, SLC22A6, ATP5O, TRPV6, SLC22A2, IL13RA2, SLC22A1, STS, LTBR, RXFP1, CCKBR, SLC22A7, SCAI, CDS1, ELMO1, ST6GALNAC1, PTGDS, TOMM22, ARL4C, KCNH5, ENOX1, ALPL, GPR182, PRF1, FUT9, USP8, CYP2F2, 4933402P03RIK, CTXN3, CLEC10A, GCGR, LRRC38, FAT2, CLEC2D, CD22, PEX14, MC3R, BAIAP2L2, NLGN1, MARCH9, SLC16A3, LSP1, CYP4F18, RAB36, CDH18, LRRC3B, PLSCR2, SLC22A18, MPZL2, WFS1, SLC6A20A, PTGIS, SLC25A22, DTL, CYP2B10, NTSR1, CNGA2, PNPLA8, CBLN1, THBD, KCNT1, GABRR1, FNBP1L, AKAP7, AKAP8, PTGFRN, NEU2, PPP5C, COBL, LMBR1, TMEM215, MRAP, STAM2, PTK7, COX7C, ASTN2, FXYD5, KCNA7, CNR2, PLCH1, MANEA, LMTK2, ADAM32, ADAM33, RPL7A, RPL4, PLXND1, HIST1H4H, HCN1, GABRA1, RABIF, SYT10, GABRA3, CKAP4, SLC6A12, H2-Q1, SLC6A13, PLEKHA4, SLC4A10, DDX55, TRIM59, RNF152, IFI27L2A, H2-EB1, SLC13A3, GRB7, SLC45A3, PRPH, LEPR, PINK1, AMN, UQCRQ, TMEM232, RAET1B, TMEM54, TMEM174, ANO4, KCNQ1, RHOH, PTGER3, ND4, MMEL1, LIMK1, ND3, SDC1, DEF6, GAP43, CPLX3, HS3ST6, CD244, SIGLECE, GALNT7, HS3ST2, GPR6, TMEM40, CDH1, CDH7, DNER, HRH4, PLCD3, HS6ST2, SNX24, KLRA1, FGFBP1, CD7, SVEP1, MUC20, ANXA1 |
| DG | up | cellular component (CC) | extracellular region（GO:0005576） | 1.15E-08 | 86 | LYPD2, CTHRC1, NRTN, AEBP1, MUP19, LEPR, OVCH2, IGFBP6, NOTUM, SERPINA3B, NOV, SCT, CTGF, SEMA3F, FAP, SEMA3B, SEMA3A, LGI2, SPON2, CASP1, WNT6, PRP2, IL13RA2, RS1, COCH, MMEL1, PRG4, OBP1A, PMCH, F9, WFDC13, CBLN4, SDC1, CBLN1, BGN, PTGDS, LAMC3, COL1A2, LAMC2, LRCH3, COL1A1, ADAMTS2, ENOX1, LALBA, VIP, PRF1, RBP4, WFIKKN2, FMOD, SERPINA11, RBP3, PAMR1, TIMP1, COL9A2, SERPINA1A, SERPINA1D, C1QTNF1, SERPINA1C, PYCARD, C2, THBS1, DKKL1, FGFBP1, BGLAP2, FN1, MUP4, BMP3, PRSS53, LYZ1, SVEP1, MUP2, EPHX3, MUC20, ANXA1, SERPINA1E, COL5A1, OMD, LAMA4, FAM180A, SRPX2, PENK, PRSS28, C1RL, BMP7, IGFBP4, TLL1 |
| DG | up | cellular component (CC) | extracellular exosome（GO:0070062） | 0.0451073 | 84 | LYPD2, PRPH, AEBP1, IGFBP6, CRABP2, ANPEP, AMN, SLK, ARHGAP1, RPL34-PS1, SEMA3B, SLC22A6, ATP5O, TRPV6, SPON2, SLC22A2, COCH, ELANE, CDHR2, F9, PADI2, COLEC12, PADI1, DNASE2A, SDC1, BGN, PTGDS, TBCA, FNBP1L, KRT16, COL1A2, IQCG, KPNA4, CUX2, ALPL, RBP4, GALNT7, PKHD1, VIM, CDH1, RRM2B, OAF, CD74, TIMP1, MYL6B, SERPINA1A, SERPINA1D, FAT2, SERPINA1C, HAAO, HS6ST2, CD22, RPL7A, RPL4, C2, THBS1, H1FOO, HIST1H4H, CD7, FN1, BMP3, LPO, LYZ1, BAIAP2L1, CKAP4, SLC6A13, H2-Q1, ANXA1, EPHX2, S100A11, SERPINA1E, COL5A1, LSP1, OMD, HSPA12A, LAMA4, DUSP26, COX2, CASP14, H2-EB1, C1RL, SLC13A3, SERPIND1, SYTL1 |
| DG | up | cellular component (CC) | extracellular space（GO:0005615） | 9.32E-06 | 68 | CTHRC1, AEBP1, MUP19, LEPR, IGFBP6, ANPEP, AMN, CXCL11, SERPINA3B, MTHFD2, SCT, PTGIS, CTGF, SEMA3F, FAP, SEMA3B, NOS2, SEMA3A, WNT6, SPON2, COCH, MMEL1, PRG4, PMCH, ELANE, F9, PDE4C, GM13304, CBLN4, THBD, PTGDS, COL1A2, LAMC2, COL1A1, ENOX1, ALPL, LALBA, RBP4, FMOD, PRF1, SERPINA11, RBP3, TIMP1, ALDH3A1, SERPINA1A, SERPINA1D, C1QTNF1, SERPINA1C, THBS1, FGFBP1, BGLAP2, FN1, MUP4, BMP3, LPO, LYZ1, MUP2, ANXA1, S100A11, SERPINA1E, OMD, SRPX2, SERPINB6C, PRSS28, C1RL, SERPIND1, BMP7, IGFBP4 |
| DG | up | cellular component (CC) | integral component of plasma membrane（GO:0005887） | 5.06E-04 | 49 | SLC22A18, SLC6A20A, LEPR, SLC16A12, KCNJ12, GPR88, KCNJ13, INSRR, SLC22A6, TRPV6, KCNQ1, SLC22A2, SLC22A1, PTGER3, LTBR, SLC22A7, IPO8, OLFR1347, NTSR1, CNGA2, SSTR5, THBD, HAS1, ROR2, KCNH5, PTK7, FXYD5, GCGR, CD74, C1QTNF1, HRH4, CLEC2D, CD22, ADRA2B, PLXND1, SLC28A1, HCN1, GABRA1, SLC6A12, SLC6A13, NLGN1, VMN1R70, SLC16A3, SLC4A10, EPHA8, SLC18A3, SLC13A3, UTS2R, SLC13A4 |
| DG | up | cellular component (CC) | proteinaceous extracellular matrix（GO:0005578） | 2.91E-05 | 23 | ALPL, COCH, CTHRC1, FMOD, RBP3, GM13304, MEPE, COL5A1, TIMP1, NOV, LAMA4, OMD, COL9A2, BGN, CTGF, LAMC3, COL1A2, LAMC2, COL1A1, WNT6, SPON2, ADAMTS2, FN1 |
| DG | up | cellular component (CC) | extracellular matrix（GO:0031012） | 7.14E-04 | 19 | COCH, FMOD, AEBP1, CKAP4, VIM, COL5A1, TIMP1, NOV, OMD, BGN, CASP14, COL1A2, ATP5O, COL1A1, THBS1, BMP7, ADAMTS2, HIST1H4H, FN1 |
| DG | up | cellular component (CC) | axon（GO:0030424） | 0.0329198 | 17 | HCN1, PRPH, COBL, NRTN, SYT2, VIM, SPHK1, PINK1, CDH1, NTSR1, NOV, CYP17A1, PENK, SLC18A3, SEMA3A, CHAT, GAP43 |
| DG | up | cellular component (CC) | basolateral plasma membrane（GO:0016323） | 0.0396406 | 11 | HCN1, LPO, SLC4A10, LEPR, SLC22A7, ANXA1, SLC22A6, CDH1, KCNQ1, SLC22A2, SLC22A1 |
| DG | up | cellular component (CC) | collagen trimer（GO:0005581） | 0.0208229 | 7 | CTHRC1, COL9A2, C1QTNF1, COL1A2, COLEC12, COL1A1, COL5A1 |
| DG | up | cellular component (CC) | basement membrane（GO:0005604） | 0.0404863 | 7 | LAMA4, LAMC3, EFNA5, LAMC2, COL5A1, FN1, TIMP1 |
| DG | up | cellular component (CC) | exocytic vesicle（GO:0070382） | 0.0105915 | 4 | SYT10, AKAP7, SYTL2, SYTL1 |
| DG | up | cellular component (CC) | female germ cell nucleus（GO:0001674） | 0.0038916 | 3 | AYM1, H1FOO, COIL |
| DG | down | biological processes (BP) | multicellular organism development(GO:0007275) | 0.0030211 | 30 | NRP2, NPNT, PRTG, SEMA5A, TCP11, CCBE1, WIPF3, ATOH8, ROBO3, ATOH7, TWIST2, NES, RREB1, MDGA1, CENPE, SIX4, COLEC11, FRZB, PROX1, SLIT1, TBATA, BVES, SEBOX, NRL, WNT9B, RYR1, NEUROD2, NHLH2, RFX3, RERE |
| DG | down | biological processes (BP) | cell differentiation(GO:0030154) | 0.0174159 | 22 | NRP2, PTCHD2, CPNE6, MDGA1, NPNT, SYT3, FRZB, SLIT1, SEMA5A, TCP11, TBATA, SEBOX, NEUROD2, NHLH2, WIPF3, RFX3, 1110017D15RIK, ATOH8, ROBO3, ATOH7, SYT17, TWIST2 |
| DG | down | biological processes (BP) | negative regulation of transcription, DNA-templated(GO:0045892) | 0.0144264 | 18 | ZFP12, ZBTB20, RREB1, MTA3, SIX4, PROX1, CD38, ZGPAT, JUN, PCGF6, PRDM5, ATF6B, RFX3, ATOH8, RASD1, LHX9, TWIST2, MT3 |
| DG | down | biological processes (BP) | cell adhesion(GO:0007155) | 0.0283665 | 15 | PARVG, CDHR1, PCDH20, NPNT, ITGA11, TMEM8, STAB2, AMIGO2, PODXL2, CDH9, BVES, PTK2B, GP1BB, PKP3, PSTPIP1 |
| DG | down | biological processes (BP) | nervous system development(GO:0007399) | 0.009278 | 14 | NRP2, DPF3, NES, NTF3, MDGA1, NRN1, SLIT1, SEMA5A, NEUROD2, GFRA1, ROBO3, ATOH8, ATOH7, RTN4IP1 |
| DG | down | biological processes (BP) | response to drug(GO:0042493) | 0.010053 | 13 | LPL, VAV3, MAT2A, SNCA, HADHA, TGFB2, CD38, PTK2B, JUN, MAS1, TBXA2R, DPYD, CCNO |
| DG | down | biological processes (BP) | negative regulation of neuron apoptotic process(GO:0043524) | 0.0048143 | 9 | HSPH1, NES, NTF3, PTK2B, JUN, CRLF1, SNCA, SIX4, MT3 |
| DG | down | biological processes (BP) | extracellular matrix organization(GO:0030198) | 0.0110121 | 7 | SMOC2, NPNT, COL27A1, OLFML2B, DMP1, SPINT1, TGFB2 |
| DG | down | biological processes (BP) | positive regulation of cytosolic calcium ion concentration(GO:0007204) | 0.0346232 | 7 | CD38, AVP, PTK2B, NPY2R, TBXA2R, PRKG1, ADRA1D |
| DG | down | biological processes (BP) | regulation of membrane potential(GO:0042391) | 0.0213315 | 6 | SLC26A4, BVES, GPR39, POPDC3, CHRNA1, SLC26A10 |
| DG | down | biological processes (BP) | circadian rhythm(GO:0007623) | 0.0320027 | 6 | AVP, MAT2A, JUN, DPYD, ATOH7, PROX1 |
| DG | down | biological processes (BP) | positive regulation of endothelial cell migration(GO:0010595) | 0.0043726 | 5 | PTK2B, CCBE1, ATOH8, BCAS3, PROX1 |
| DG | down | biological processes (BP) | response to hormone(GO:0009725) | 0.0076877 | 5 | RERG, CD38, MAT2A, PTK2B, GHSR |
| DG | down | biological processes (BP) | cellular response to calcium ion(GO:0071277) | 0.0108607 | 5 | JUN, CPNE6, CPNE7, NEUROD2, KRT10 |
| DG | down | biological processes (BP) | SMAD protein signal transduction(GO:0060395) | 0.037158 | 5 | JUN, GDF11, GDF10, ATOH8, TGFB2 |
| DG | down | biological processes (BP) | positive regulation of smooth muscle contraction(GO:0045987) | 0.0067501 | 4 | NPNT, NPY2R, TBXA2R, ADRA1D |
| DG | down | biological processes (BP) | ERK1 and ERK2 cascade(GO:0070371) | 0.0084658 | 4 | AVP, MAP2K1, FGF10, MT3 |
| DG | down | biological processes (BP) | positive regulation of epithelial cell migration(GO:0010634) | 0.0206369 | 4 | RREB1, JUN, FGF10, TGFB2 |
| DG | down | biological processes (BP) | embryonic cranial skeleton morphogenesis(GO:0048701) | 0.0254922 | 4 | DLX1AS, WNT9B, SIX4, TWIST2 |
| DG | down | biological processes (BP) | positive regulation of cell-substrate adhesion(GO:0010811) | 0.0309068 | 4 | SMOC2, NPNT, NPY2R, DMP1 |
| DG | down | biological processes (BP) | positive regulation of DNA replication(GO:0045740) | 0.0328352 | 4 | JUN, MAS1, FGF10, KCTD13 |
| DG | down | biological processes (BP) | positive regulation of vasoconstriction(GO:0045907) | 0.0368757 | 4 | CD38, AVP, TBXA2R, ADRA1D |
| DG | down | biological processes (BP) | tube formation(GO:0035148) | 0.0069606 | 3 | SDCCAG8, ATOH8, BCAS3 |
| DG | down | biological processes (BP) | carbohydrate biosynthetic process(GO:0016051) | 0.0109484 | 3 | B3GAT2, CHST9, CHST11 |
| DG | down | biological processes (BP) | positive regulation of transcription from RNA polymerase II promoter in response to endoplasmic reticulum stress(GO:1990440) | 0.0132383 | 3 | ATF6, ATF6B, CREB3L3 |
| DG | down | biological processes (BP) | keratinocyte proliferation(GO:0043616) | 0.0183759 | 3 | KLK8, FST, FGF10 |
| DG | down | biological processes (BP) | diet induced thermogenesis(GO:0002024) | 0.0183759 | 3 | GPR39, MC4R, OMA1 |
| DG | down | biological processes (BP) | embryonic camera-type eye development(GO:0031076) | 0.0212104 | 3 | NES, ALDH1A3, FGF10 |
| DG | down | biological processes (BP) | long term synaptic depression(GO:0060292) | 0.0307002 | 3 | CD38, PTK2B, DRD5 |
| DG | down | biological processes (BP) | induction of positive chemotaxis(GO:0050930) | 0.0341722 | 3 | NTF3, IL16, FGF10 |
| DG | down | biological processes (BP) | positive regulation of dendrite extension(GO:1903861) | 0.0377887 | 3 | CPNE6, SYT3, SYT17 |
| DG | down | biological processes (BP) | regulation of G1/S transition of mitotic cell cycle(GO:2000045) | 0.0377887 | 3 | BID, PSME3, TJP3 |
| DG | down | biological processes (BP) | positive regulation of neural precursor cell proliferation(GO:2000179) | 0.0454333 | 3 | NES, PTCHD2, PROX1 |
| DG | down | molecular function (MF) | protein binding(GO:0005515) | 0.0199394 | 85 | NRP2, STK33, IL16, PDLIM5, GRIK4, SYT3, FGF10, RRAD, PRKG1, TGFB2, HSPH1, CASP6, HOMER3, RALA, LGI3, ROBO3, CHRNA1, LOXL1, MAPKBP1, THRSP, TWIST2, NDNL2, CDHR1, MTA3, ANKS3, BSN, KRT10, SIX4, PROX1, MCM6, EVC2, BVES, HIST2H2BE, JUN, SRGAP3, C1QL2, DSP, ADAMTS1, CHORDC1, BID, TYRP1, WFIKKN1, SDCCAG8, IL1R1, NR3C2, PRKDC, ATP6V1G2, DUSP19, PTK2B, ZAP70, PSTPIP1, AUTS2, EHD1, LHX9, VPS39, LPL, NES, VAV3, CPNE4, NTF3, MAP2K1, IMMT, CPNE6, CIDEA, TEAD1, FRZB, ATF6, CSPP1, RASL11A, TBATA, IRF5, PKP2, PKP3, HPCA, GDF11, MC4R, NEUROD2, LSM11, TREML4, RFX3, FCGBP, LRP2, TJP3, PDZD3, RERE |
| DG | down | molecular function (MF) | calcium ion binding(GO:0005509) | 1.26E-04 | 27 | GALNT3, PCDH20, NPNT, SYT3, SNCA, CABP7, KCNIP2, CANT1, SMOC2, DGKB, CDH9, CCBE1, EHD1, PCDHB9, CDHR1, STAB2, SLIT1, UMODL1, HPCA, C2CD4C, RYR1, C2CD4B, C2CD4A, CHORDC1, LRP2, SYT17, TBC1D8B |
| DG | down | molecular function (MF) | protein N-terminus binding(GO:0047485) | 0.0468902 | 6 | MAP2K1, PDLIM5, SNCA, KCNIP2, GTF2H2, TGFB2 |
| DG | down | molecular function (MF) | calcium-dependent phospholipid binding(GO:0005544) | 0.0077934 | 5 | SYT3, C2CD4C, C2CD4B, C2CD4A, SYT17 |
| DG | down | molecular function (MF) | copper ion binding(GO:0005507) | 0.0190258 | 5 | TYRP1, AHCY, SNCA, LOXL1, MT3 |
| DG | down | molecular function (MF) | cAMP response element binding(GO:0035497) | 0.001172 | 4 | ATF6, JUN, ATF6B, CREB3L3 |
| DG | down | molecular function (MF) | N-acetylgalactosamine 4-sulfate 6-O-sulfotransferase activity(GO:0050659) | 0.0331955 | 2 | CHST11, CHST15 |
| DG | down | molecular function (MF) | iodide transmembrane transporter activity(GO:0015111) | 0.0493791 | 2 | SLC26A4, SLC5A5 |
| DG | down | cellular component (CC) | membrane(GO:0016020) | 8.65E-06 | 148 | SLC9A4, B3GALT5, SLC44A5, GM11744, PDLIM5, PRTG, SNCA, SYT3, GRIK4, RRAD, PRKG1, SLC26A10, IL17RD, ZNRF3, DMXL2, HOMER3, ATF6B, RALA, CREB3L3, ROBO3, CHRNA1, 1810041L15RIK, ORC2, OMA1, MDGA1, CDHR1, TANC1, SIPA1L3, TMEM126B, KRT10, TECR, TMEM28, CD38, EVC2, PODXL2, NAPEPLD, SLITRK3, TMEM138, ATP9A, RYR1, CD300LG, DSP, ORAI2, IL1R1, SLC38A7, PCDH20, DRD5, ITGA11, EPHA10, NRN1, SLCO2A1, SEMA5A, TCP11, SLC29A4, ZAP70, LPL, MAP2K1, CPNE6, MNDAL, CPNE7, CRAT, ATF6, B3GAT2, RERG, MYO10, EPHA6, TCTA, FAAH, GPR39, GRINA, TRAF3IP3, TSGA10, TBXA2R, TREML4, HIST1H3D, GHSR, SLC5A10, PDZD3, SYT17, ADRA1D, NRP2, SLC5A5, SEC1, ENOX2, NPNT, KCNIP2, CANT1, TMEM171, CHST11, CHST15, KCNG3, JPH1, ZFYVE1, RTN4IP1, PARVG, CCDC88B, WBSCR17, CLMN, NOL9, CAR12, DDN, ELMO2, SLC26A4, AMIGO2, KLRG1, BVES, UMODL1, CHST9, PSME3, SLC27A3, RASD1, BID, GALNT3, TYRP1, FAM69B, PTCHD2, NPY2R, NR3C2, CD109, PRKDC, CABP7, GPR1, SHISA6, DGKB, CDH9, PTK2B, GP1BB, GLIPR1, RNF128, MAS1, PSTPIP1, POPDC3, EHD1, PEX10, VPS39, IMMT, GJB3, CLDN22, TMEM8, CENPE, STAB2, TSPAN18, RASL11A, MC4R, GFRA1, PAPOLG, LRP2, TJP3 |
| DG | down | cellular component (CC) | extracellular region(GO:0005576) | 0.0107925 | 41 | IL1R1, WFIKKN1, ENOX2, IL16, NPNT, FST, SNCA, CRLF1, CD109, FGF10, TGFB2, HSPH1, SMOC2, GLIPR1, COL27A1, IMPG1, CCBE1, LGI3, PRSS35, LOXL1, LPL, AVP, KLK8, NTF3, OLFML2B, DMP1, SPINT1, COLEC11, FRZB, CELA1, SLIT1, SERPINA3N, C4BP, IL18BP, CLEC18A, UMODL1, WNT9B, GDF11, C1QL2, GDF10, ADAMTS1 |
| DG | down | cellular component (CC) | extracellular space(GO:0005615) | 0.0044535 | 38 | IL1R1, ENOX2, IL16, PRTG, SNCA, CD109, FGF10, NRN1, FBRS, TGFB2, DMXL2, CCBE1, KLK1B27, LOXL1, MT3, LPL, AVP, KLK8, HIST1H2BE, MDGA1, KLK1B24, SIPA1L3, SPINT1, KRT10, FRZB, CELA1, SLIT1, SERPINA3N, ADAMTS9, IL18BP, UMODL1, HIST2H2BE, WNT9B, GDF11, GFRA1, GDF10, LRP2, KLHL34 |
| DG | down | cellular component (CC) | cell junction(GO:0030054) | 0.0192147 | 20 | PARVG, SDCCAG8, PDLIM5, SNCA, GRIK4, GJB3, TANC1, BSN, CLDN22, NRN1, BVES, DMXL2, PTK2B, HOMER3, PKP2, PKP3, DSP, LGI3, CHRNA1, TJP3 |
| DG | down | cellular component (CC) | axon(GO:0030424) | 0.0022741 | 15 | NRP2, IL1R1, MAP2K1, DRD5, SLC38A7, CPNE6, SNCA, GRIK4, BSN, TGFB2, PTK2B, HPCA, GFRA1, ROBO3, MT3 |
| DG | down | cellular component (CC) | proteinaceous extracellular matrix(GO:0005578) | 0.0116953 | 12 | SMOC2, ADAMTS9, NPNT, COL27A1, OLFML2B, CCBE1, DMP1, WNT9B, ADAMTS1, FCGBP, LOXL1, SLIT1 |
| ACC | up | biological processes (BP) | proteolysis（GO:0006508） | 0.0485766 | 11 | ACR, ACY1, CASP14, CNDP2, RBP3, PRSS28, ELANE, RHBDL1, HTRA3, PRSS37, CTRL |
| ACC | up | biological processes (BP) | metabolic process（GO:0008152） | 0.0123493 | 11 | MAN2B2, ACY1, ACY3, CNDP2, GM13178, UAP1L1, GM438, FAHD2A, ACSF3, GM436, GSTM7 |
| ACC | up | biological processes (BP) | visual learning（GO:0008542） | 0.0214821 | 4 | PPP1R1B, GRIN1, SYNGAP1, RGS14 |
| ACC | up | biological processes (BP) | catabolic process（GO:0009056） | 0.0074243 | 3 | GM13178, GM438, GM436 |
| ACC | up | biological processes (BP) | cellular process（GO:0009987） | 0.0211773 | 3 | FMNL1, CDHR1, LTA |
| ACC | up | biological processes (BP) | long-term memory（GO:0007616） | 0.040502 | 3 | CCND2, GRIN1, RGS14 |
| ACC | up | biological processes (BP) | DNA methylation involved in embryo development（GO:0043045） | 0.0461374 | 2 | ZFP57, TRIM28 |
| ACC | up | molecular function (MF) | serine-type peptidase activity（GO:0008236） | 0.0045776 | 7 | ACR, RBP3, PRSS28, ELANE, RHBDL1, HTRA3, CTRL |
| ACC | up | molecular function (MF) | serine-type endopeptidase activity（GO:0004252） | 0.0155673 | 7 | ACR, PRSS28, ELANE, RHBDL1, HTRA3, PRSS37, CTRL |
| ACC | up | molecular function (MF) | aminoacylase activity（GO:0004046） | 0.0373009 | 2 | ACY1, ACY3 |
| ACC | up | cellular component (CC) | nucleolus（GO:0005730） | 3.75E-04 | 19 | CEBPA, MRI1, TBL3, TAOK2, RBM3, TRIM28, RRP8, H1FX, RRP9, GGN, TUT1, TSPYL2, CCND2, GRWD1, ACTL6B, CIRBP, PARP1, APEX1, SPATA24 |
| ACC | up | cellular component (CC) | cell junction（GO:0030054） | 0.0223523 | 13 | PARVG, KCNAB2, BAIAP2L2, FERMT3, GRIN1, RGS14, PTK2B, LRFN1, CHRNB4, SYNGAP1, SEZ6, CDC42BPB, CHRNA2 |
| ACC | up | cellular component (CC) | postsynaptic density（GO:0014069） | 0.0178416 | 7 | KCNAB2, PTK2B, GRIN1, LRFN1, EEF2K, SYNGAP1, RGS14 |
| ACC | down | biological processes (BP) | negative regulation of transcription, DNA-templated（GO:0045892） | 0.0335397 | 10 | ID2, SALL1, TRPS1, PCGF6, HEYL, ARNTL, NRG1, FOXN3, CLOCK, FOXS1 |
| ACC | down | biological processes (BP) | positive regulation of gene expression（GO:0010628） | 0.0335282 | 8 | WNT10A, EZR, ID2, CRH, GJA1, NRG1, NFIL3, PIK3R1 |
| ACC | down | biological processes (BP) | innate immune response（GO:0045087） | 0.0339161 | 8 | KLRG1, CASP4, S100A8, CAMP, S100A9, MALT1, YES1, MID2 |
| ACC | down | biological processes (BP) | cell migration（GO:0016477） | 0.0034887 | 7 | CTHRC1, ASAP3, NRG1, EMP2, FNDC3B, KDR, PRPF40A |
| ACC | down | biological processes (BP) | heart development（GO:0007507） | 0.0151058 | 7 | FLRT3, ID2, SALL1, TRPS1, CCDC151, GJA1, NRG1 |
| ACC | down | biological processes (BP) | circadian rhythm（GO:0007623） | 0.0014308 | 6 | ID2, ARNTL, NFIL3, PPARGC1A, CLOCK, FBXL3 |
| ACC | down | biological processes (BP) | rhythmic process（GO:0048511） | 0.0030089 | 6 | ID2, ARNTL, NFIL3, PPARGC1A, CLOCK, FBXL3 |
| ACC | down | biological processes (BP) | circadian regulation of gene expression（GO:0032922） | 0.0113388 | 4 | ID2, ARNTL, PPARGC1A, CLOCK |
| ACC | down | biological processes (BP) | positive regulation of inflammatory response（GO:0050729） | 0.0123742 | 4 | S100A8, S100A9, ADAM8, CLOCK |
| ACC | down | biological processes (BP) | negative regulation of protein kinase activity（GO:0006469） | 0.0362094 | 4 | FLRT3, WWTR1, PKIA, GADD45A |
| ACC | down | biological processes (BP) | cellular response to hypoxia（GO:0071456） | 0.0442997 | 4 | TRPC6, SIRT4, ADAM8, PPARGC1A |
| ACC | down | biological processes (BP) | leukocyte migration involved in inflammatory response（GO:0002523） | 0.0042685 | 3 | S100A8, S100A9, ADAM8 |
| ACC | down | biological processes (BP) | olfactory bulb development（GO:0021772） | 0.0154655 | 3 | ID2, SALL1, LRRK2 |
| ACC | down | biological processes (BP) | protein O-linked glycosylation（GO:0006493） | 0.0179161 | 3 | GALNT1, C1GALT1C1, C1GALT1 |
| ACC | down | biological processes (BP) | cellular response to ionizing radiation（GO:0071479） | 0.0232699 | 3 | FIGNL1, GADD45A, CLOCK |
| ACC | down | biological processes (BP) | cell communication（GO:0007154） | 0.035648 | 3 | GJD2, KREMEN2, GJA1 |
| ACC | down | biological processes (BP) | establishment of protein localization to plasma membrane（GO:0090002） | 0.0425966 | 3 | EZR, RAB13, TSPAN15 |
| ACC | down | biological processes (BP) | O-glycan processing, core 1（GO:0016267） | 0.015206 | 2 | C1GALT1C1, C1GALT1 |
| ACC | down | biological processes (BP) | neutrophil aggregation（GO:0070488） | 0.015206 | 2 | S100A8, S100A9 |
| ACC | down | biological processes (BP) | regulation of type B pancreatic cell development（GO:2000074） | 0.0227227 | 2 | ARNTL, CLOCK |
| ACC | down | biological processes (BP) | positive regulation of peptide secretion（GO:0002793） | 0.0301824 | 2 | S100A8, S100A9 |
| ACC | down | biological processes (BP) | positive regulation of behavioral fear response（GO:2000987） | 0.0375856 | 2 | CRH, GJA1 |
| ACC | down | biological processes (BP) | endothelium development（GO:0003158） | 0.0375856 | 2 | GJA1, KDR |
| ACC | down | biological processes (BP) | peptidyl-cysteine S-nitrosylation（GO:0018119） | 0.0375856 | 2 | S100A8, S100A9 |
| ACC | down | biological processes (BP) | protein kinase A signaling（GO:0010737） | 0.0449326 | 2 | EZR, RAB13 |
| ACC | down | biological processes (BP) | negative regulation of glucocorticoid receptor signaling pathway（GO:2000323） | 0.0449326 | 2 | ARNTL, CLOCK |
| ACC | down | biological processes (BP) | regulation of hair cycle（GO:0042634） | 0.0449326 | 2 | ARNTL, CLOCK |
| ACC | down | molecular function (MF) | protein binding（GO:0005515） | 0.0032695 | 45 | XPO1, CTHRC1, CLDN5, GJA1, TAS1R3, LZTFL1, ZFP110, SESN3, N4BP2, EZR, CASP8AP2, SAA1, HTRA1, SNTB2, ADAM8, NRG1, YES1, NFIL3, PIK3R1, PRPF40A, GJD2, FLRT3, TRPC6, L3MBTL3, SIRT4, MALT1, ARNTL, WWTR1, PPARGC1A, FZD6, KDR, GLUL, FCGR2B, KREMEN2, ID2, TRPS1, SALL1, CD274, HEYL, SLC18A3, LRRK2, GADD45A, CLOCK, PLAU, FBXL3 |
| ACC | down | molecular function (MF) | hydrolase activity（GO:0016787） | 0.0310781 | 19 | MTHFD2L, FIGNL1, HEXB, SIRT4, ACOT1, MALT1, PTPN21, AGMAT, LPIN3, CASP4, HTRA1, ACOT11, NUDT7, CTSC, PRSS16, ADAM8, NEU3, PLAU, TLL1 |
| ACC | down | molecular function (MF) | sequence-specific DNA binding（GO:0043565） | 0.0435285 | 10 | TRPS1, HEYL, CREB3L4, ARNTL, NFIL3, PPARGC1A, FOXN3, CLOCK, ZFP110, FOXS1 |
| ACC | down | molecular function (MF) | protein domain specific binding（GO:0019904） | 0.0198487 | 7 | XPO1, HNRNPM, EZR, PCGF6, GJA1, HNRNPC, PIK3R1 |
| ACC | down | molecular function (MF) | integrin binding（GO:0005178） | 0.0372966 | 4 | EDIL3, ADAM8, EMP2, KDR |
| ACC | down | molecular function (MF) | protein kinase A catalytic subunit binding（GO:0034236） | 0.0052684 | 3 | EZR, RAB13, PKIA |
| ACC | down | molecular function (MF) | clathrin binding（GO:0030276） | 0.0448212 | 3 | SYT4, TRPC6, LRRK2 |
| ACC | down | molecular function (MF) | manganese ion binding（GO:0030145） | 0.0465936 | 3 | GLUL, GALNT1, NUDT7 |
| ACC | down | molecular function (MF) | glycoprotein-N-acetylgalactosamine 3-beta-galactosyltransferase activity（GO:0016263） | 0.0146204 | 2 | C1GALT1C1, C1GALT1 |
| ACC | down | molecular function (MF) | Toll-like receptor 4 binding（GO:0035662） | 0.0361543 | 2 | S100A8, S100A9 |
| ACC | down | cellular component (CC) | cytoplasm（GO:0005737） | 4.62E-04 | 68 | ALDH8A1, SAT1, XPO1, CTHRC1, S100A8, FIGNL1, FAM110C, FST, S100A9, GJA1, ACOT1, PTPN21, LZTFL1, CASP4, CASP8AP2, HTRA1, ACOT11, SNTB2, KLHL24, ADAM8, NRG1, TMCO6, HMGCLL1, CAMP, RINT1, IRF2BP2, ARNTL, DAPK2, ARRDC3, PPARGC1A, PKIA, GLUL, TNFAIP8, PCMTD2, RAB13, LRRK2, WDR44, NEU3, EMP2, GADD45A, FBXL3, CLOCK, AFAP1L2, ASAP3, CMPK1, ZFP110, SESN3, EZR, YES1, PIK3R1, PRPF40A, FLRT3, SSRP1, TRPC6, FAM111A, MALT1, WWTR1, ELAVL4, MID2, KDR, ID2, FCGR2B, SALL1, CCDC151, HEYL, TUBD1, CRH, SLC18A3 |
| ACC | down | cellular component (CC) | membrane（GO:0016020） | 0.0063576 | 66 | XPO1, SC5D, S100A8, SYT4, S100A9, HEXB, CLDN5, GJA1, TAS1R3, SDC4, KCNU1, CASP4, HTRA1, ST3GAL4, SNTB2, CREB3L4, ADAM8, TMEM107, SV2C, FNDC3B, GJD2, TMCO6, C1GALT1C1, HMGCLL1, TMEM167B, RIMBP2, RINT1, CHST5, ARRDC3, KLRG1, CTSC, GPR17, RAB13, LRRK2, WDR44, NEU3, EMP2, GGCX, GALNT1, PHKA1, STK17B, HNRNPM, LINGO4, EZR, PCDHB18, HNRNPC, YES1, PIK3R1, C1GALT1, PRPF40A, TMEM45A, FLRT3, PCNX, MTHFD2L, TRPC6, B3GALT2, XKR6, TSPAN15, ELAVL4, KDR, FZD6, FCGR2B, KREMEN2, CD274, ABCC4, PLAU |
| ACC | down | cellular component (CC) | extracellular exosome（GO:0070062） | 0.0140211 | 30 | ALDH8A1, S100A8, FIGNL1, CLDN5, S100A9, HEXB, GJA1, EDIL3, SDC4, AGMAT, CMPK1, HNRNPM, EZR, ST3GAL4, HTRA1, SAA1, ACOT11, SNTB2, HNRNPC, YES1, C1GALT1C1, CAMP, TSPAN15, MID2, GLUL, CD274, CTSC, RAB13, LRRK2, PLAU |
| ACC | down | cellular component (CC) | cytosol（GO:0005829） | 0.0161773 | 22 | SAT1, HMGCLL1, ACOT1, GJA1, MALT1, ARNTL, ELAVL4, PPARGC1A, ZFP110, N4BP2, EZR, ID2, HTRA1, ACOT11, HNRNPC, WDR44, LRRK2, YES1, EMP2, PIK3R1, FBXL3, CLOCK |
| ACC | down | cellular component (CC) | extracellular region（GO:0005576） | 0.0453514 | 20 | FLRT3, WNT10A, CTHRC1, GALNT1, S100A8, CAMP, S100A9, FST, EDIL3, SDC4, KDR, CASP4, FCGR2B, HTRA1, SAA1, CRH, PRAP1, NRG1, PLAU, TLL1 |
| ACC | down | cellular component (CC) | Golgi apparatus（GO:0005794） | 0.0466211 | 15 | GALNT1, B3GALT2, SYT4, TMEM167B, GJA1, CHST5, KDR, ST3GAL4, CTSC, CREB3L4, RAB13, WDR44, LRRK2, YES1, EMP2 |
| ACC | down | cellular component (CC) | cytoplasmic vesicle（GO:0031410） | 0.0069037 | 12 | SYT4, SLC18A3, SNTB2, PRSS16, CREB3L4, RAB13, DAPK2, LRRK2, SV2C, EMP2, KDR, FZD6 |
| ACC | down | cellular component (CC) | cell junction（GO:0030054） | 0.0341383 | 11 | GJD2, FLRT3, SYT4, RIMBP2, CLDN5, SNTB2, GJA1, RAB13, LRRK2, SV2C, KDR |
| ACC | down | cellular component (CC) | intracellular membrane-bounded organelle（GO:0043231） | 0.0440954 | 11 | GGCX, XPO1, SC5D, GLUL, CGRRF1, SYT4, AASS, ARNTL, NEU3, LRRK2, CLOCK |
| ACC | down | cellular component (CC) | cell surface（GO:0009986） | 0.0375624 | 10 | HNRNPM, FCGR2B, CD274, TSPAN15, ADAM8, SDC4, EMP2, PLAU, KDR, FZD6 |
| ACC | down | cellular component (CC) | neuron projection（GO:0043005） | 0.0109407 | 9 | GLUL, CASP4, SYT4, SLC18A3, RAB13, LRRK2, SV2C, PIK3R1, KDR |
| ACC | down | cellular component (CC) | focal adhesion（GO:0005925） | 0.0228176 | 8 | FLRT3, EZR, SNTB2, GJA1, ASAP3, YES1, SDC4, PLAU |
| ACC | down | cellular component (CC) | membrane raft（GO:0045121） | 0.011639 | 7 | EZR, FCGR2B, GJA1, LRRK2, SDC4, EMP2, KDR |
| ACC | down | cellular component (CC) | synaptic vesicle membrane（GO:0030672） | 0.0095936 | 4 | SYT4, SLC18A3, LRRK2, SV2C |

**Table S4. The enriched KEGG pathways of the key differentially expressed genes in DG and ACC**

| **Sample** | **gene expression** | **Term** | **P-value** | **Count** | **Genes** |
| --- | --- | --- | --- | --- | --- |
| DG | up | Neuroactive ligand-receptor interaction（mmu04080） | 0.00464023 | 17 | GABRA1, PTGER3, CCKBR, RXFP1, GABRA3, LEPR, NTSR1, GCGR, SSTR5, GABRR1, HRH4, CNR2, CHRNA4, UTS2R, MC3R, ADRA2B, CHRNA2 |
| DG | up | ECM-receptor interaction（mmu04512） | 0.00259217 | 9 | LAMA4, SDC1, LAMC3, COL1A2, LAMC2, COL1A1, THBS1, COL5A1, FN1 |
| DG | up | Amoebiasis（mmu05146） | 0.01412787 | 9 | LAMA4, LAMC3, SERPINB6C, COL1A2, LAMC2, COL1A1, NOS2, COL5A1, FN1 |
| DG | up | Complement and coagulation cascades（mmu04610） | 0.00442349 | 8 | THBD, SERPINA1A, SERPINA1D, SERPINA1C, F9, SERPINA1E, SERPIND1, C2 |
| DG | up | Salivary secretionmmu04970） | 0.0047564 | 8 | LPO, PRB1, LYZ1, ADCY8, GUCY1B2, GUCY1A3, PRKG2, TRPV6 |
| DG | down | Calcium signaling pathway（mmu04020） | 0.02506084 | 8 | ORAI2, CD38, ATP2B3, PTK2B, DRD5, RYR1, TBXA2R, ADRA1D |
| DG | down | Hepatitis B（mmu05161） | 0.02930289 | 7 | EGR3, MAP2K1, PTK2B, JUN, ATF6B, CREB3L3, TGFB2 |
| DG | down | Thyroid hormone synthesis（mmu04918） | 0.02541138 | 5 | SLC26A4, SLC5A5, ATF6B, CREB3L3, LRP2 |
| DG | down | Hematopoietic cell lineage（mmu04640） | 0.04685471 | 5 | CD38, IL1R1, GP5, DNTT, GP1BB |
| ACC | up | Toxoplasmosis（mmu05145） | 0.01660124 | 5 | LAMB3, NFKBIB, MAP2K3, BIRC7, H2-DMA |
| ACC | dowm | Proteoglycans in cancer（mmu05205） | 0.00822676 | 7 | WNT10A, EZR, SDC4, PLAU, PIK3R1, KDR, FZD6 |
| ACC | dowm | Signaling pathways regulating pluripotency of stem cells（mmu04550） | 0.03187373 | 5 | WNT10A, ID2, PCGF6, PIK3R1, FZD6 |
| ACC | dowm | Mucin type O-Glycan biosynthesis（mmu00512） | 0.02442757 | 3 | GALNT1, C1GALT1C1, C1GALT1 |
| ACC | dowm | Circadian rhythm（mmu04710） | 0.02955308 | 3 | ARNTL, CLOCK, FBXL3 |

**Table S5. Topology table for the key differentially expressed genes in DG and ACC**

| **Sample** | **regualtion** | **node** | **Betweenness Centrality** | **Degree** |
| --- | --- | --- | --- | --- |
| DG | up | Fn1 | 0.20648335 | 44 |
| DG | up | Wfs1 | 0.14437992 | 15 |
| DG | up | Kcnq1 | 0.1290103 | 12 |
| DG | up | Adcy8 | 0.12888893 | 12 |
| DG | up | Cnga2 | 0.1034601 | 10 |
| DG | up | Cdh1 | 0.08568976 | 19 |
| DG | up | Col1a1 | 0.07464542 | 25 |
| DG | up | Serpinb6c | 0.0728637 | 13 |
| DG | up | Ptgs2 | 0.07153921 | 22 |
| DG | up | Cthrc1 | 0.06436908 | 5 |
| DG | up | Penk | 0.06412605 | 25 |
| DG | up | Sdc1 | 0.05415833 | 13 |
| DG | up | Vim | 0.05289245 | 16 |
| DG | up | Sema3a | 0.04922054 | 6 |
| DG | up | Gap43 | 0.04818002 | 6 |
| DG | up | Dcaf13 | 0.04802549 | 11 |
| DG | up | Adra2b | 0.04698996 | 15 |
| DG | up | Rtp2 | 0.04465939 | 11 |
| DG | up | Rps21 | 0.04366364 | 18 |
| DG | up | Bglap2 | 0.04094458 | 13 |
| DG | up | Timp1 | 0.04040203 | 26 |
| DG | up | Itpa | 0.03752927 | 7 |
| DG | up | Alpl | 0.03485765 | 11 |
| DG | up | Chat | 0.0346987 | 8 |
| DG | up | Anxa1 | 0.03383167 | 20 |
| DG | up | Leo1 | 0.03247015 | 7 |
| DG | up | Pole4 | 0.03190727 | 5 |
| DG | up | Rpl30 | 0.03179297 | 18 |
| DG | up | Casp1 | 0.03092232 | 8 |
| DG | up | Ctgf | 0.03062542 | 15 |
| DG | up | Kcnt1 | 0.03043408 | 7 |
| DG | up | Dck | 0.03019371 | 5 |
| DG | up | Cxcr3 | 0.02990036 | 16 |
| DG | up | Drd1a | 0.02903685 | 9 |
| DG | up | Kdf1 | 0.02880127 | 3 |
| DG | up | Thbs1 | 0.02800267 | 19 |
| DG | up | Syt2 | 0.02722409 | 8 |
| DG | up | Tnnc2 | 0.02637772 | 8 |
| DG | up | BC048507 | 0.02531489 | 4 |
| DG | up | Slc18a3 | 0.02519859 | 8 |
| DG | up | Tnnt2 | 0.02501864 | 8 |
| DG | up | Dtl | 0.02496971 | 4 |
| DG | up | Mrpl1 | 0.02414597 | 14 |
| DG | up | Sdad1 | 0.02344355 | 13 |
| DG | up | Kcna7 | 0.02309123 | 7 |
| DG | up | Serpind1 | 0.02300001 | 18 |
| DG | up | Lgi2 | 0.02289223 | 2 |
| DG | up | Elane | 0.02287794 | 14 |
| DG | up | Snai1 | 0.02194713 | 13 |
| DG | up | Pmch | 0.02170349 | 18 |
| DG | up | Cplx3 | 0.02160688 | 3 |
| DG | up | Dkc1 | 0.02126512 | 11 |
| DG | up | Sf3a2 | 0.02109588 | 4 |
| DG | up | Dgkk | 0.02069814 | 3 |
| DG | up | Pcp2 | 0.02044264 | 14 |
| DG | up | Efna5 | 0.02026292 | 5 |
| DG | up | Arhgef16 | 0.01993678 | 3 |
| DG | up | Lrrc3b | 0.01989899 | 2 |
| DG | up | Bmp7 | 0.01980947 | 14 |
| DG | up | Rrm2b | 0.01952999 | 5 |
| DG | up | Gbp5 | 0.01913823 | 4 |
| DG | up | Chrna4 | 0.01907211 | 6 |
| DG | up | Hs3st2 | 0.01891615 | 3 |
| DG | up | Ptgis | 0.01877946 | 5 |
| DG | up | Col1a2 | 0.01841145 | 17 |
| DG | up | Uqcrq | 0.01834121 | 10 |
| DG | up | Prf1 | 0.01831786 | 9 |
| DG | up | Zfp455 | 0.01807441 | 2 |
| DG | up | Rps23 | 0.01761859 | 16 |
| DG | up | Gcgr | 0.01739765 | 12 |
| DG | up | Ppp2r5e | 0.01686984 | 5 |
| DG | up | Rpl34 | 0.01680576 | 16 |
| DG | up | Prkg2 | 0.0165374 | 6 |
| DG | up | Zic4 | 0.01610044 | 5 |
| DG | up | Mc3r | 0.0160521 | 9 |
| DG | up | Rbp3 | 0.01584879 | 5 |
| DG | up | Vip | 0.0157294 | 6 |
| DG | up | Gucy1a3 | 0.01559085 | 5 |
| DG | up | Colec12 | 0.01518307 | 4 |
| DG | up | Ckap4 | 0.01498722 | 13 |
| DG | up | Cdh18 | 0.01498722 | 3 |
| DG | up | Stam2 | 0.01496203 | 4 |
| DG | up | Plcd3 | 0.01496203 | 2 |
| DG | up | Gabra1 | 0.01489021 | 4 |
| DG | up | Ifit1 | 0.01437277 | 6 |
| DG | up | Serpina3b | 0.01437021 | 11 |
| DG | up | Foxc1 | 0.01430025 | 3 |
| DG | up | Muc20 | 0.01411352 | 3 |
| DG | up | Dlx3 | 0.01405197 | 3 |
| DG | up | Sart1 | 0.01347579 | 4 |
| DG | up | Gadd45a | 0.01326531 | 3 |
| DG | up | Epha8 | 0.01323101 | 3 |
| DG | up | Lyz1 | 0.01320708 | 7 |
| DG | up | Plxnd1 | 0.01306217 | 6 |
| DG | up | Lypd2 | 0.01298686 | 6 |
| DG | up | Ppp1r3b | 0.01297884 | 4 |
| DG | up | Nlgn1 | 0.01279348 | 3 |
| DG | up | Atp5o | 0.01238806 | 11 |
| DG | up | Cd3e | 0.01224262 | 7 |
| DG | up | F9 | 0.01205222 | 9 |
| DG | up | Pde4c | 0.01188782 | 2 |
| DG | up | Ptger3 | 0.01186617 | 14 |
| DG | up | Iqcg | 0.01162761 | 3 |
| DG | up | Cyp2b10 | 0.01158522 | 8 |
| DG | up | Agpat9 | 0.01157837 | 2 |
| DG | up | Cabp5 | 0.01153145 | 4 |
| DG | up | Nr1i3 | 0.01150943 | 3 |
| DG | up | Rpl4 | 0.01118986 | 15 |
| DG | up | Mepe | 0.01113794 | 14 |
| DG | up | Dpp10 | 0.01107611 | 4 |
| DG | up | Fgfbp1 | 0.01101712 | 4 |
| DG | up | Kcnj13 | 0.01046678 | 3 |
| DG | up | Bgn | 0.0103734 | 17 |
| DG | up | Gtf2h5 | 0.01028102 | 5 |
| DG | up | Gpr6 | 0.01015526 | 3 |
| DG | up | Usp8 | 0.01001247 | 3 |
| DG | up | Rhoh | 0.01001247 | 3 |
| DG | up | Pip5kl1 | 0.01001247 | 3 |
| DG | up | Fnbp1l | 0.00999987 | 4 |
| DG | up | Lepr | 0.00987894 | 5 |
| DG | up | Ddx55 | 0.00968285 | 8 |
| DG | up | Cdyl2 | 0.00967467 | 3 |
| DG | up | Lhx3 | 0.00964718 | 5 |
| DG | up | Nos2 | 0.00953391 | 6 |
| DG | up | Slc22a7 | 0.00949793 | 5 |
| DG | up | Meis2 | 0.00946312 | 5 |
| DG | up | Slc22a2 | 0.00938876 | 5 |
| DG | up | Foxc2 | 0.00936449 | 6 |
| DG | up | Ptk7 | 0.00932411 | 3 |
| DG | up | Igfbp4 | 0.00930287 | 12 |
| DG | up | Gabrr1 | 0.00905805 | 6 |
| DG | up | Anpep | 0.00898585 | 7 |
| DG | up | Tcap | 0.00880537 | 7 |
| DG | up | Ntsr1 | 0.00854465 | 6 |
| DG | up | Rpl7a | 0.00836177 | 13 |
| DG | up | Gm4978 | 0.00836177 | 13 |
| DG | up | Lin28a | 0.00825119 | 4 |
| DG | up | Gm10639 | 0.00819228 | 5 |
| DG | up | Dner | 0.00807303 | 3 |
| DG | up | Klf5 | 0.00805796 | 4 |
| DG | up | Fmod | 0.00790263 | 12 |
| DG | up | Serpina1a | 0.00781055 | 17 |
| DG | up | Serpina1d | 0.00781055 | 17 |
| DG | up | Kif5b | 0.00770727 | 3 |
| DG | up | Satb2 | 0.00763799 | 3 |
| DG | up | Prg4 | 0.0075494 | 6 |
| DG | up | Oas1f | 0.00751314 | 9 |
| DG | up | Cds1 | 0.00746624 | 2 |
| DG | up | Ints3 | 0.00743779 | 3 |
| DG | up | Pycard | 0.00725859 | 7 |
| DG | up | Serpina1e | 0.00724728 | 16 |
| DG | up | Cox7c | 0.0071015 | 9 |
| DG | up | Fam180a | 0.00696571 | 4 |
| DG | up | Astn2 | 0.00678067 | 2 |
| DG | up | Rec8 | 0.0067008 | 3 |
| DG | up | Cyp17a1 | 0.00658341 | 3 |
| DG | up | Mettl7a2 | 0.00655876 | 4 |
| DG | up | Lypd5 | 0.00637052 | 5 |
| DG | up | Grb7 | 0.0062634 | 3 |
| DG | up | Mybpc1 | 0.00622552 | 6 |
| DG | up | Syt10 | 0.00616704 | 3 |
| DG | up | Cckbr | 0.00615729 | 6 |
| DG | up | Lama4 | 0.00600823 | 7 |
| DG | up | Slc22a18 | 0.00597358 | 5 |
| DG | up | Rdh12 | 0.00597241 | 4 |
| DG | up | Pink1 | 0.00594395 | 3 |
| DG | up | Nbn | 0.00585445 | 3 |
| DG | up | Manea | 0.00583795 | 2 |
| DG | up | Aldh1a2 | 0.00551869 | 3 |
| DG | up | Aldh3a1 | 0.0054828 | 3 |
| DG | up | Crabp2 | 0.00546855 | 3 |
| DG | up | Chrna2 | 0.00544865 | 4 |
| DG | up | Ep400 | 0.00528736 | 2 |
| DG | up | Plk2 | 0.00527093 | 2 |
| DG | up | Col5a1 | 0.00507044 | 15 |
| DG | up | Tll1 | 0.00505919 | 5 |
| DG | up | Klra1 | 0.00505022 | 4 |
| DG | up | Prrx2 | 0.00503142 | 4 |
| DG | up | Padi2 | 0.00501253 | 5 |
| DG | up | Hs3st6 | 0.00501253 | 3 |
| DG | up | Gpr97 | 0.00501253 | 2 |
| DG | up | Mapk13 | 0.00501253 | 2 |
| DG | up | Gpr88 | 0.00501253 | 2 |
| DG | up | Akap7 | 0.00501253 | 2 |
| DG | up | Erich6 | 0.00501253 | 2 |
| DG | up | Vsig8 | 0.00501253 | 2 |
| DG | up | Myo3b | 0.00501253 | 2 |
| DG | up | Vmn1r65 | 0.00501253 | 2 |
| DG | up | Tshz3 | 0.00501253 | 2 |
| DG | up | Ipo8 | 0.00501253 | 2 |
| DG | up | Car7 | 0.00501253 | 2 |
| DG | up | Zfp37 | 0.00501253 | 2 |
| DG | up | Fhod3 | 0.00501253 | 2 |
| DG | up | Knstrn | 0.00501253 | 2 |
| DG | up | Cnr2 | 0.00485449 | 13 |
| DG | up | Cbln1 | 0.00471633 | 3 |
| DG | up | Nr5a1 | 0.00464627 | 3 |
| DG | up | Slc6a12 | 0.00460367 | 3 |
| DG | up | Galnt7 | 0.00448849 | 2 |
| DG | up | Ly6g6e | 0.00448547 | 3 |
| DG | up | Cbln4 | 0.0044635 | 3 |
| DG | up | Gucy1b2 | 0.0042605 | 4 |
| DG | up | Wfikkn2 | 0.0041769 | 2 |
| DG | up | Rpl17 | 0.0041598 | 15 |
| DG | up | Tra2b | 0.00403517 | 2 |
| DG | up | Mrap | 0.00396945 | 3 |
| DG | up | H2-Q1 | 0.00380299 | 6 |
| DG | up | Slc16a12 | 0.00372966 | 2 |
| DG | up | Spon2 | 0.00346071 | 3 |
| DG | up | Gm16381 | 0.00326428 | 2 |
| DG | up | H2-Eb1 | 0.00318486 | 4 |
| DG | up | Sstr5 | 0.00312582 | 12 |
| DG | up | Slc22a6 | 0.00302405 | 5 |
| DG | up | Coch | 0.00300711 | 2 |
| DG | up | Myh2 | 0.002992 | 6 |
| DG | up | Slc28a1 | 0.0027845 | 3 |
| DG | up | Gm17669 | 0.00275999 | 11 |
| DG | up | Myh4 | 0.00267131 | 5 |
| DG | up | Sema3b | 0.00267061 | 3 |
| DG | up | Ppp5c | 0.0026616 | 2 |
| DG | up | Notum | 0.00263537 | 12 |
| DG | up | Mettl7a3 | 0.00256153 | 3 |
| DG | up | Cd22 | 0.00238986 | 4 |
| DG | up | Unc13c | 0.00237404 | 2 |
| DG | up | Slc6a20a | 0.00229691 | 3 |
| DG | up | Rs1 | 0.00224738 | 2 |
| DG | up | Rapsn | 0.00217881 | 3 |
| DG | up | Adamts2 | 0.00209109 | 9 |
| DG | up | Slc13a3 | 0.00204085 | 3 |
| DG | up | Taf13 | 0.00203968 | 2 |
| DG | up | Klf15 | 0.002039 | 2 |
| DG | up | Gabra3 | 0.00203472 | 3 |
| DG | up | Lzts3 | 0.00197003 | 2 |
| DG | up | Bmp3 | 0.00196855 | 3 |
| DG | up | Thbd | 0.0019474 | 3 |
| DG | up | Slc6a13 | 0.00194116 | 2 |
| DG | up | Slc13a4 | 0.00191769 | 2 |
| DG | up | Myl6b | 0.0018412 | 3 |
| DG | up | Cd74 | 0.00173014 | 3 |
| DG | up | Pou3f2 | 0.0017235 | 3 |
| DG | up | Fut9 | 0.00146594 | 2 |
| DG | up | Uts2r | 0.00146147 | 6 |
| DG | up | Vax2 | 0.00142741 | 2 |
| DG | up | Arid4b | 0.00129533 | 2 |
| DG | up | Mpzl2 | 0.00127273 | 3 |
| DG | up | Ptgds | 0.00119744 | 4 |
| DG | up | Cd7 | 0.0011792 | 5 |
| DG | up | Hist1h4h | 9.95E-04 | 2 |
| DG | up | Tshz2 | 9.90E-04 | 2 |
| DG | up | Ifi44 | 9.26E-04 | 4 |
| DG | up | Oas2 | 9.26E-04 | 4 |
| DG | up | Serpina1c | 9.01E-04 | 5 |
| DG | up | St6galnac1 | 8.89E-04 | 2 |
| DG | up | Cish | 8.02E-04 | 2 |
| DG | up | Sct | 7.29E-04 | 6 |
| DG | up | Haao | 6.72E-04 | 2 |
| DG | up | Stat5b | 6.10E-04 | 2 |
| DG | up | Hemt1 | 5.28E-04 | 3 |
| DG | up | Wbscr22 | 4.05E-04 | 5 |
| DG | up | Ephx2 | 3.96E-04 | 3 |
| DG | up | mt-Co3 | 3.62E-04 | 7 |
| DG | up | Coa6 | 3.44E-04 | 7 |
| DG | up | C1qtnf1 | 3.14E-04 | 2 |
| DG | up | Sema3f | 2.95E-04 | 2 |
| DG | up | Lmbr1 | 2.93E-04 | 2 |
| DG | up | Foxd1 | 2.93E-04 | 2 |
| DG | up | Slc22a1 | 2.28E-04 | 3 |
| DG | up | Gm4953 | 1.90E-04 | 5 |
| DG | up | Col9a2 | 1.33E-04 | 6 |
| DG | up | mt-Nd3 | 1.25E-04 | 4 |
| DG | up | Utf1 | 1.21E-04 | 3 |
| DG | up | Igfbp6 | 1.11E-04 | 2 |
| DG | up | Omd | 1.04E-04 | 2 |
| DG | up | Sphk1 | 8.95E-05 | 2 |
| DG | up | Hs6st2 | 7.56E-05 | 3 |
| DG | up | Srp54a | 6.18E-05 | 10 |
| DG | up | Wnt6 | 2.80E-05 | 2 |
| DG | up | mt-Nd4 | 3.15E-06 | 4 |
| DG | up | Utp20 | 0 | 3 |
| DG | up | Tomm22 | 0 | 1 |
| DG | up | Aff4 | 0 | 2 |
| DG | up | Olfr522 | 0 | 1 |
| DG | up | Lamc3 | 0 | 1 |
| DG | up | Arhgap1 | 0 | 1 |
| DG | up | Alox12e | 0 | 3 |
| DG | up | Arhgap25 | 0 | 1 |
| DG | up | Limk1 | 0 | 2 |
| DG | up | Eif3j1 | 0 | 2 |
| DG | up | Tas2r113 | 0 | 11 |
| DG | up | Tas2r109 | 0 | 11 |
| DG | up | Pex14 | 0 | 2 |
| DG | up | Rxfp1 | 0 | 5 |
| DG | up | Siglece | 0 | 3 |
| DG | up | Olfr1415 | 0 | 1 |
| DG | up | Nrtn | 0 | 1 |
| DG | up | Cdh7 | 0 | 1 |
| DG | up | Olfr173 | 0 | 1 |
| DG | up | Hrh4 | 0 | 11 |
| DG | up | Cyp4f18 | 0 | 3 |
| DG | up | Asap1 | 0 | 1 |
| DG | up | Kcnj12 | 0 | 1 |
| DG | up | Olfr671 | 0 | 1 |
| DG | up | Gm10081 | 0 | 1 |
| DG | up | Olfr1466 | 0 | 1 |
| DG | up | Folr4 | 0 | 3 |
| DG | up | Olfr319 | 0 | 1 |
| DG | up | Trappc2 | 0 | 3 |
| DG | up | Olfr1252 | 0 | 1 |
| DG | up | Olfr1229 | 0 | 1 |
| DG | up | Bglap3 | 0 | 2 |
| DG | up | Rapgef1 | 0 | 1 |
| DG | up | S100a11 | 0 | 2 |
| DG | up | Pinx1 | 0 | 4 |
| DG | up | Plch1 | 0 | 1 |
| DG | up | Dbt | 0 | 1 |
| DG | up | Akap8 | 0 | 1 |
| DG | up | Kansl1 | 0 | 1 |
| DG | up | Cux2 | 0 | 2 |
| DG | up | Dusp10 | 0 | 1 |
| DG | up | Tox4 | 0 | 1 |
| DG | up | Fat2 | 0 | 1 |
| DG | up | Ifi27l2a | 0 | 3 |
| DG | up | Rlbp1 | 0 | 2 |
| DG | up | Ttc39b | 0 | 1 |
| DG | up | Apobec3 | 0 | 5 |
| DG | up | Prr9 | 0 | 1 |
| DG | up | Adam33 | 0 | 1 |
| DG | up | Cdh20 | 0 | 1 |
| DG | up | Nudt4 | 0 | 6 |
| DG | up | Fap | 0 | 2 |
| DG | up | Tfap4 | 0 | 1 |
| DG | up | Tfcp2l1 | 0 | 2 |
| DG | up | Cd244 | 0 | 2 |
| DG | up | Coil | 0 | 1 |
| DG | up | Rasgef1b | 0 | 2 |
| DG | up | Olfr544 | 0 | 2 |
| DG | up | Mei1 | 0 | 1 |
| DG | up | Olfr97 | 0 | 1 |
| DG | up | Lrrc38 | 0 | 1 |
| DG | up | Pdcl3 | 0 | 1 |
| DG | up | Cdc42ep3 | 0 | 1 |
| DG | up | Clec2d | 0 | 1 |
| DG | up | Glis1 | 0 | 1 |
| DG | up | Sox13 | 0 | 1 |
| DG | up | Tes | 0 | 1 |
| DG | up | Gm4907 | 0 | 1 |
| DG | up | Kpna4 | 0 | 1 |
| DG | up | Dkkl1 | 0 | 1 |
| DG | up | Tmem215 | 0 | 1 |
| DG | up | Pkhd1 | 0 | 1 |
| DG | up | Ier5l | 0 | 1 |
| DG | up | Lrch3 | 0 | 1 |
| DG | up | Tgif2lx1 | 0 | 1 |
| DG | up | Elmo1 | 0 | 1 |
| DG | up | Aebp1 | 0 | 4 |
| DG | up | Crxos | 0 | 1 |
| DG | up | Sept1 | 0 | 1 |
| DG | up | Pitpnm3 | 0 | 1 |
| DG | up | Slc25a22 | 0 | 1 |
| DG | up | Gins3 | 0 | 1 |
| DG | up | Otud1 | 0 | 1 |
| DG | up | ENSMUSG00000079001 | 0 | 1 |
| DG | up | Lamc2 | 0 | 2 |
| DG | up | Sowahb | 0 | 1 |
| DG | up | Myo1h | 0 | 1 |
| DG | up | Capn11 | 0 | 1 |
| DG | up | Rbp4 | 0 | 2 |
| DG | up | Atp6ap1l | 0 | 1 |
| DG | up | Vmn1r70 | 0 | 1 |
| DG | up | Zmat3 | 0 | 2 |
| DG | up | Nlrc3 | 0 | 2 |
| DG | up | Dusp26 | 0 | 1 |
| DG | up | Rai14 | 0 | 1 |
| DG | up | Kcnh5 | 0 | 1 |
| DG | up | Arl4c | 0 | 2 |
| DG | up | Krt16 | 0 | 1 |
| DG | up | Tspo2 | 0 | 1 |
| DG | up | Vat1l | 0 | 1 |
| DG | up | Adrm1 | 0 | 2 |
| DG | up | Scai | 0 | 1 |
| DG | up | Brinp3 | 0 | 1 |
| DG | up | Mup19 | 0 | 1 |
| DG | up | Cobl | 0 | 1 |
| DG | up | Hspa12a | 0 | 1 |
| DG | up | Dmrt2 | 0 | 1 |
| DG | up | Nov | 0 | 1 |
| DG | up | Asgr1 | 0 | 1 |
| DG | up | Catsper4 | 0 | 1 |
| DG | up | Secisbp2l | 0 | 1 |
| DG | up | Casp14 | 0 | 1 |
| DG | up | Nfe2l3 | 0 | 1 |
| DG | up | E13 | 0 | 1 |
| DG | up | Vmn1r48 | 0 | 1 |
| DG | up | Sult2a8 | 0 | 1 |
| DG | up | Ctxn3 | 0 | 1 |
| DG | up | Atp10b | 0 | 1 |
| DG | up | Trim59 | 0 | 1 |
| DG | up | Has1 | 0 | 1 |
| DG | up | Mmel1 | 0 | 1 |
| DG | up | Neu2 | 0 | 1 |
| DG | down | Prkdc | 0.37402745 | 4 |
| DG | down | Fkbp4 | 0.36441122 | 4 |
| DG | down | Dntt | 0.34795437 | 5 |
| DG | down | Nes | 0.33786826 | 3 |
| DG | down | Avp | 0.33700498 | 7 |
| DG | down | Nr3c2 | 0.33530029 | 3 |
| DG | down | Fgf10 | 0.29727249 | 3 |
| DG | down | Drd5 | 0.29707579 | 4 |
| DG | down | Jun | 0.27299152 | 9 |
| DG | down | Srgap3 | 0.26160504 | 2 |
| DG | down | Slit1 | 0.25380278 | 5 |
| DG | down | Fst | 0.18043535 | 4 |
| DG | down | Sema5a | 0.17886179 | 5 |
| DG | down | Cd38 | 0.13961011 | 4 |
| DG | down | Cdh9 | 0.13571991 | 2 |
| DG | down | Vav3 | 0.13223402 | 4 |
| DG | down | Ptk2b | 0.12929452 | 7 |
| DG | down | Hist2h2be | 0.122738 | 4 |
| DG | down | Pkp3 | 0.12168896 | 4 |
| DG | down | Ntf3 | 0.11158099 | 3 |
| DG | down | Twist2 | 0.10739575 | 2 |
| DG | down | Zap70 | 0.10665268 | 4 |
| DG | down | Elmo2 | 0.10293732 | 3 |
| DG | down | Wnt9b | 0.09349593 | 3 |
| DG | down | Gdf11 | 0.09284028 | 3 |
| DG | down | Stab2 | 0.08589038 | 2 |
| DG | down | Nrn1 | 0.0848304 | 6 |
| DG | down | Evc2 | 0.08202203 | 2 |
| DG | down | Atoh7 | 0.07802255 | 2 |
| DG | down | Wbscr17 | 0.07108357 | 4 |
| DG | down | Prkg1 | 0.06460355 | 5 |
| DG | down | Mc4r | 0.06333596 | 4 |
| DG | down | Nrl | 0.06294256 | 2 |
| DG | down | Frzb | 0.06294256 | 2 |
| DG | down | Mta3 | 0.04825597 | 3 |
| DG | down | Map2k1 | 0.04799371 | 4 |
| DG | down | Hist1h3d | 0.04786258 | 3 |
| DG | down | Gjb3 | 0.04786258 | 4 |
| DG | down | Dusp19 | 0.04786258 | 3 |
| DG | down | Cdhr1 | 0.04786258 | 3 |
| DG | down | Smoc2 | 0.04760031 | 2 |
| DG | down | Hpca | 0.04760031 | 2 |
| DG | down | Dsp | 0.04707579 | 5 |
| DG | down | Ryr1 | 0.04685724 | 4 |
| DG | down | Cpne4 | 0.04631087 | 4 |
| DG | down | Auts2 | 0.04617974 | 3 |
| DG | down | Shisa6 | 0.04615788 | 4 |
| DG | down | Mdga1 | 0.03952487 | 4 |
| DG | down | Atf6 | 0.03614826 | 4 |
| DG | down | Atp2b3 | 0.03260775 | 3 |
| DG | down | Epha6 | 0.03212693 | 4 |
| DG | down | Atp6v1c2 | 0.0319958 | 3 |
| DG | down | Tbxa2r | 0.0319958 | 4 |
| DG | down | Nhlh2 | 0.0319958 | 2 |
| DG | down | Tbc1d8b | 0.0319958 | 2 |
| DG | down | Tjp3 | 0.03094676 | 4 |
| DG | down | Dpf3 | 0.01612903 | 2 |
| DG | down | Hsph1 | 0.01612903 | 3 |
| DG | down | Slc26a4 | 0.01612903 | 2 |
| DG | down | Rere | 0.01612903 | 2 |
| DG | down | Galnt3 | 0.01612903 | 2 |
| DG | down | Pcgf6 | 0.01612903 | 2 |
| DG | down | Nol9 | 0.01612903 | 2 |
| DG | down | Zbtb20 | 0.01612903 | 2 |
| DG | down | Gpr115 | 0.01612903 | 2 |
| DG | down | Hadha | 0.01612903 | 2 |
| DG | down | Sebox | 0.01612903 | 2 |
| DG | down | Bcat2 | 0.01612903 | 2 |
| DG | down | Zcwpw1 | 0.01494886 | 2 |
| DG | down | Creb3l3 | 0.00448029 | 3 |
| DG | down | Atf6b | 0.00448029 | 3 |
| DG | down | Bcas3 | 0.00413061 | 2 |
| DG | down | Nrp2 | 0.00360608 | 4 |
| DG | down | Rfx3 | 0.00321269 | 2 |
| DG | down | Cabp7 | 0.00118017 | 2 |
| DG | down | Pkp2 | 0 | 3 |
| DG | down | Atp6v1g2 | 0 | 2 |
| DG | down | Jph1 | 0 | 2 |
| DG | down | Hist1h2be | 0 | 1 |
| DG | down | Ghsr | 0 | 3 |
| DG | down | Robo3 | 0 | 2 |
| DG | down | Adra1d | 0 | 3 |
| DG | down | Adamts9 | 0 | 2 |
| DG | down | Adamts1 | 0 | 2 |
| DG | down | Il17rd | 0 | 1 |
| DG | down | Epha10 | 0 | 1 |
| DG | down | Lrp2 | 0 | 1 |
| DG | down | Cd109 | 0 | 2 |
| DG | down | Chordc1 | 0 | 2 |
| DG | down | Grpel2 | 0 | 1 |
| DG | down | Dmxl2 | 0 | 2 |
| DG | down | Grik4 | 0 | 3 |
| DG | down | Vwa3a | 0 | 1 |
| DG | down | Slc5a5 | 0 | 1 |
| DG | down | Egr3 | 0 | 1 |
| DG | down | Wfikkn1 | 0 | 2 |
| DG | down | Zfp608 | 0 | 1 |
| DG | down | Dmp1 | 0 | 1 |
| DG | down | Lrrc10b | 0 | 2 |
| DG | down | Mprip | 0 | 1 |
| DG | down | Traf3ip3 | 0 | 1 |
| DG | down | Clmp | 0 | 2 |
| DG | down | Nrros | 0 | 1 |
| DG | down | Ddn | 0 | 2 |
| DG | down | Papolg | 0 | 1 |
| DG | down | Mapkbp1 | 0 | 1 |
| DG | down | Tcta | 0 | 1 |
| DG | down | Dock11 | 0 | 1 |
| DG | down | Ndnl2 | 0 | 1 |
| DG | down | Lrrc46 | 0 | 1 |
| DG | down | Fbrs | 0 | 1 |
| DG | down | Kcnip2 | 0 | 1 |
| DG | down | Ankef1 | 0 | 1 |
| DG | down | Klk8 | 0 | 1 |
| DG | down | Six4 | 0 | 1 |
| DG | down | Syna | 0 | 1 |
| DG | down | Krt10 | 0 | 1 |
| DG | down | Khdc3 | 0 | 1 |
| DG | down | Idh3b | 0 | 1 |
| DG | down | Tead1 | 0 | 1 |
| DG | down | Pdlim5 | 0 | 1 |
| DG | down | Rala | 0 | 1 |
| DG | down | Orai2 | 0 | 2 |
| DG | down | Pcdhb9 | 0 | 1 |
| DG | down | Dnah1 | 0 | 1 |
| ACC | up | Acr | 0.66666667 | 3 |
| ACC | up | Prkcg | 0.64731183 | 6 |
| ACC | up | Entpd6 | 0.57142857 | 2 |
| ACC | up | Syngap1 | 0.53010753 | 7 |
| ACC | up | Cant1 | 0.52380952 | 3 |
| ACC | up | Grwd1 | 0.36031746 | 5 |
| ACC | up | Parp1 | 0.34285714 | 3 |
| ACC | up | Egln1 | 0.32258065 | 2 |
| ACC | up | Apex1 | 0.31904762 | 3 |
| ACC | up | Kcnab2 | 0.29677419 | 4 |
| ACC | up | Psmf1 | 0.29462366 | 4 |
| ACC | up | Sycp3 | 0.28571429 | 2 |
| ACC | up | Grin1 | 0.25913978 | 6 |
| ACC | up | Trim28 | 0.25714286 | 3 |
| ACC | up | Rrp8 | 0.24761905 | 4 |
| ACC | up | Rrp9 | 0.2031746 | 5 |
| ACC | up | Itgb7 | 0.18494624 | 4 |
| ACC | up | Mrpl1 | 0.13809524 | 3 |
| ACC | up | Rad23a | 0.13492063 | 4 |
| ACC | up | Jsrp1 | 0.13333333 | 2 |
| ACC | up | Cdc23 | 0.12473118 | 2 |
| ACC | up | Tbl3 | 0.07301587 | 5 |
| ACC | up | Chrnb4 | 0.06451613 | 2 |
| ACC | up | Rtn4rl2 | 0.06451613 | 2 |
| ACC | up | Map2k3 | 0.06451613 | 2 |
| ACC | up | Mri1 | 0.06451613 | 2 |
| ACC | up | Eef2k | 0.06451613 | 2 |
| ACC | up | Ddn | 0.06451613 | 2 |
| ACC | up | Kcnh3 | 0 | 1 |
| ACC | up | Rasal1 | 0 | 1 |
| ACC | up | Lrfn1 | 0 | 2 |
| ACC | up | Chrna2 | 0 | 1 |
| ACC | up | Cntn5 | 0 | 1 |
| ACC | up | Taok2 | 0 | 1 |
| ACC | up | Nfkbib | 0 | 1 |
| ACC | up | Lta | 0 | 1 |
| ACC | up | Elane | 0 | 1 |
| ACC | up | Mettl7a3 | 0 | 1 |
| ACC | up | Ptk2b | 0 | 1 |
| ACC | up | Parvg | 0 | 2 |
| ACC | up | Zfp57 | 0 | 1 |
| ACC | up | Wdr6 | 0 | 2 |
| ACC | up | Ppm1g | 0 | 1 |
| ACC | up | Srm | 0 | 1 |
| ACC | up | Fermt3 | 0 | 2 |
| ACC | up | Lamb3 | 0 | 1 |
| ACC | up | Nanos3 | 0 | 1 |
| ACC | up | Ppp1r1b | 0 | 1 |
| ACC | up | Map3k15 | 0 | 1 |
| ACC | up | Ubl7 | 0 | 2 |
| ACC | up | Tmem82 | 0 | 1 |
| ACC | up | Myo3b | 0 | 1 |
| ACC | up | Prss37 | 0 | 1 |
| ACC | up | Tbrg4 | 0 | 2 |
| ACC | up | Pdzd9 | 0 | 1 |
| ACC | up | Bzw2 | 0 | 1 |
| ACC | dowm | Pik3r1 | 0.56236559 | 5 |
| ACC | dowm | Kdr | 0.55913978 | 4 |
| ACC | dowm | Gpr17 | 0.42688172 | 4 |
| ACC | dowm | Prpf40a | 0.41111111 | 5 |
| ACC | dowm | Id2 | 0.40860215 | 3 |
| ACC | dowm | Nfil3 | 0.36129032 | 4 |
| ACC | dowm | Plau | 0.36129032 | 2 |
| ACC | dowm | Ssrp1 | 0.35555556 | 3 |
| ACC | dowm | Adam8 | 0.32258065 | 2 |
| ACC | dowm | Hnrnpc | 0.32222222 | 5 |
| ACC | dowm | Neu3 | 0.27956989 | 2 |
| ACC | dowm | St3gal4 | 0.23225806 | 3 |
| ACC | dowm | Bbs10 | 0.2 | 2 |
| ACC | dowm | Xpo1 | 0.2 | 3 |
| ACC | dowm | Fbxl3 | 0.12688172 | 5 |
| ACC | dowm | Gja1 | 0.12688172 | 4 |
| ACC | dowm | C1galt1 | 0.12473118 | 4 |
| ACC | dowm | Fcgr2b | 0.11935484 | 4 |
| ACC | dowm | Hnrnpm | 0.11111111 | 4 |
| ACC | dowm | Klrg1 | 0.07419355 | 3 |
| ACC | dowm | Cd274 | 0.06451613 | 3 |
| ACC | dowm | Ppargc1a | 0.06451613 | 3 |
| ACC | dowm | Arntl | 0.06021505 | 4 |
| ACC | dowm | Clock | 0.06021505 | 4 |
| ACC | dowm | Yes1 | 0.00430108 | 3 |
| ACC | dowm | Sirt4 | 0 | 1 |
| ACC | dowm | Casp4 | 0 | 1 |
| ACC | dowm | Glul | 0 | 1 |
| ACC | dowm | Nkapl | 0 | 1 |
| ACC | dowm | Elavl4 | 0 | 1 |
| ACC | dowm | Alyref2 | 0 | 2 |
| ACC | dowm | Emp2 | 0 | 1 |
| ACC | dowm | Afap1l2 | 0 | 1 |
| ACC | dowm | Cldn5 | 0 | 2 |
| ACC | dowm | Klhl24 | 0 | 1 |
| ACC | dowm | Lztfl1 | 0 | 1 |
| ACC | dowm | Gjd2 | 0 | 1 |
| ACC | dowm | Galnt1 | 0 | 2 |
| ACC | dowm | Tas1r3 | 0 | 1 |
| ACC | dowm | Nrg1 | 0 | 2 |
| ACC | dowm | Pkia | 0 | 1 |
| ACC | dowm | B3galt2 | 0 | 2 |
| ACC | dowm | C1galt1c1 | 0 | 2 |
